# Elucidation of the regio- and chemoselectivity of enzymatic allylic oxidations with *Pleurotus sapidus* – conversion of selected spirocyclic terpenoids and computational analysis

**DOI:** 10.3762/bjoc.9.262

**Published:** 2013-10-29

**Authors:** Verena Weidmann, Mathias Schaffrath, Holger Zorn, Julia Rehbein, Wolfgang Maison

**Affiliations:** 1 Department of Chemistry, University of Hamburg, Bundesstr. 45, 20146 Hamburg, Germany; 2 LGCR Chemistry, Sanofi-Aventis Deutschland GmbH, 65926 Frankfurt am Main, Germany; 3 Justus-Liebig-University Giessen, Institute of Food Chemistry and Food Biotechnology, Heinrich-Buff-Ring 58, 35392 Gießen; 4 Department of Chemistry, University of Hamburg, Martin-Luther-King-Platz 6, 20146 Hamburg, Germany

**Keywords:** allylic oxidation, CH-activation, chiral separation, enones, flavors, natural products, terpenes

## Abstract

Allylic oxidations of olefins to enones allow the efficient synthesis of value-added products from simple olefinic precursors like terpenes or terpenoids. Biocatalytic variants have a large potential for industrial applications, particularly in the pharmaceutical and food industry. Herein we report efficient biocatalytic allylic oxidations of spirocyclic terpenoids by a lyophilisate of the edible fungus *Pleurotus sapidus*. This ‘’mushroom catalysis’’ is operationally simple and allows the conversion of various unsaturated spirocyclic terpenoids. A number of new spirocyclic enones have thus been obtained with good regio- and chemoselectivity and chiral separation protocols for enantiomeric mixtures have been developed. The oxidations follow a radical mechanism and the regioselectivity of the reaction is mainly determined by bond-dissociation energies of the available allylic CH-bonds and steric accessibility of the oxidation site.

## Introduction

**Figure 1:**
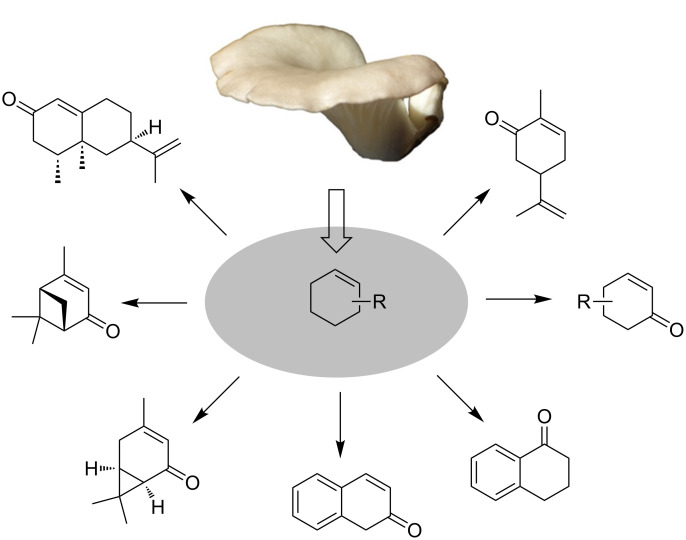
Selected biocatalytic allylic and benzylic oxidations with the lyophilisate of *Pleurotus sapidus* (PSA).

Selective oxidations of CH-bonds are attractive synthetic transformations with a broad spectrum of applications in academia and a high impact on the industrial chemical value chain as they convert relatively cheap precursors into value-added products [1–2]. Among these transformations, allylic oxidations are of high interest because the olefinic starting materials are readily available as cheap bulk chemicals and many interesting derivatives such as terpenes are available from renewable sources [3–5]. In addition, the resulting allyl alcohols [6–10] or α,β-unsaturated carbonyl compounds are attractive synthetic targets of high economic and scientific interest [11–17]. Allylic oxidations of olefins to enones have classically been performed with strong oxidants such as chromium or other metal-based reagents [18–19]. In addition, metal-free and biocatalytic methods have been reported [5]. Several of these biocatalytic protocols have been applied to the synthesis of fine chemicals [20–22], drugs [23], and food ingredients [24–26]. A particularly interesting biocatalytic system for allylic oxidation is the edible fungus *Pleurotus sapidus* (PSA), which is able to oxidize selected terpenes and fatty acids [27–31]. We have recently shown that the lyophilisate of PSA is able to affect allylic and benzylic oxidations in a broad range of olefinic substrates including simple cyclohexene derivatives and several functionalized terpenoids (Figure 1) [32].

Biocatalytic allylic oxidations with PSA may be performed with the lyophilisate from submerged cultures. Cyclic alkenes and particularly cyclohexene derivatives are the preferred substrates of PSA. A PSA-derived dioxygenase has been shown to be responsible for the allylic oxidation of valencene to nootkatone, and the same enzyme oxidizes unsaturated fatty acids [29,31]. It is thus likely that this dioxygenase is the major oxidant in other allylic oxidations with PSA-lyophilisate, too. However, since the lyophilisate is a mixture of enzymes, alternative oxidation pathways cannot be ruled out for other substrates. Reviewing the available examples for PSA-mediated conversions in the literature, the regio- and chemoselectivity of these oxidations seems to be determined by the radical mechanism of the reactions and would thus follow well-established rules for other radical-type allylic oxidations [33–36].

**Scheme 1:**
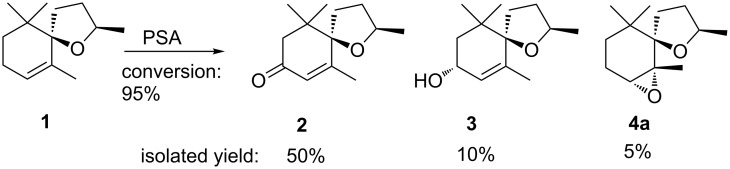
Biocatalytic allylic oxidation of theaspirane (**1**) with lyophilisates of PSA. Only one enantiomer of racemic compounds is shown.

A notable example of an allylic oxidation with PSA is the conversion of theaspirane (**1**), a spirocyclic flavor compound of tea, vanilla and different fruits [37] to the corresponding theaspirone (**2**) (Scheme 1) [32].

**Figure 2:**
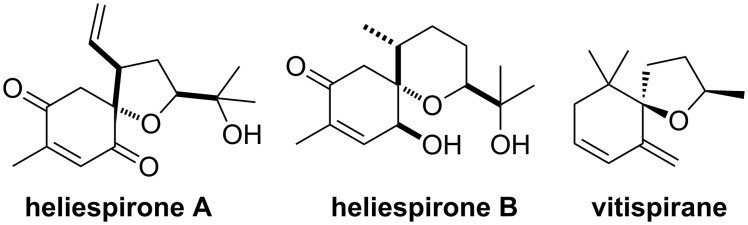
Selected bioactive terpenoids based on spiroether backbones [38–39].

The reaction is quite clean and gives the enone **2** in good yield along with minor amounts of the corresponding allyl alcohol **3** and the epoxide **4a**. This successful conversion of theaspirane (**1**) encouraged us to investigate the oxidation of other spirocyclic terpenoids. Many oxidized spiroethers are valuable flavor compounds or have other interesting biological properties such as phytotoxic activity. A few selected examples are depicted in Figure 2.

In this paper, we report biocatalytic allylic oxidations of spirocyclic model compounds and of the natural product vitispirane with the lyophilisate of PSA. A rationalization of the observed selectivity is provided by means of computational determination of bond-dissociation enthalpies and correlation with structural and electronic features. In addition, a short synthesis of vitispirane is presented.

## Results and Discussion

**Scheme 2:**
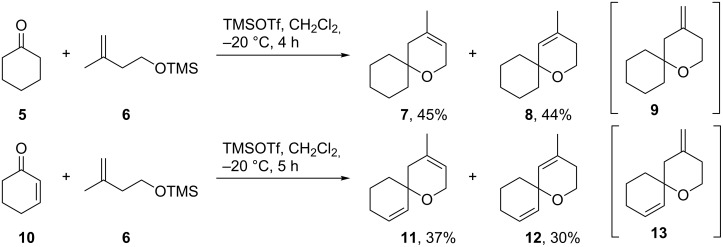
Intramolecular silyl modified Sakurai reaction to spiroethers **7**–**9** and **11**–**13**.

Allylic spiroethers, such as theaspirane (**1**) (Scheme 1) are suitable substrates for allylic oxidations with PSA. Due to their interesting properties as flavor compounds, we focused our attention to the oxidation of terpenoid spiroethers. As model compounds, unsaturated spiroethers **7**, **8**, **11** and **12** were synthesized by the intramolecular silyl-modified Sakurai reaction of precursors **5** and **10** and alkene **6** (Scheme 2). Two pairs of regioisomeric endocyclic derivatives **7**, **8** and **11**, **12** were obtained as the major products of cyclization in a 1:1 mixture. The third regioisomer **9** and **13** respectively, with an exocyclic double bond was observed in minor amounts only. Separation of the regioisomeric spiroethers by column chromatography proved to be difficult, and in both cases only the two major isomers with endocyclic double bonds were isolated in pure form. The regioisomeric spiroethers **7**, **8**, **11** and **12** are interesting model compounds for the allylic oxidation with PSA because they contain allylic positions with different stereoelectronic properties.

**Scheme 3:**
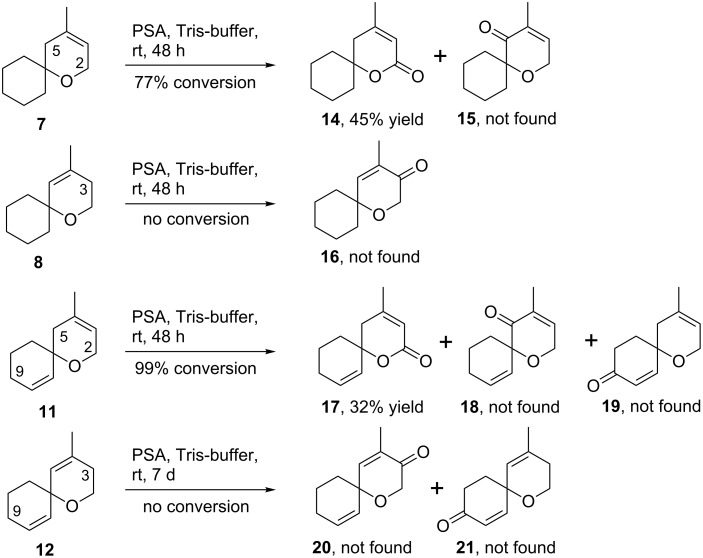
Biocatalytic allylic oxidation of spiroethers **7**, **8**, **11** and **12** with the lyophilisate of PSA. Conversion was measured by GC and yields are given after isolation of the oxidation products and purification by chromatography.

All four spiroethers **7**, **8**, **11** and **12** were submitted to identical conditions and were treated with PSA lyophilisate in Tris-buffer at room temperature (Scheme 3). The reactions were monitored by GC. The oxidation of allyl ether **7** may follow two alternative pathways either to the α,β-unsaturated lactone **14** or to the enone **15** (allylic oxidations at exocyclic positions are generally less favorable if radical mechanisms are operating) [35]. Due to sterical hindrance at position 5 and the strong activation for hydrogen abstraction in position 2, spiroether **7** was selectively converted to the spirolactone **14**. The alternative oxidation product **15** was not found by GC–MS. The regioisomeric spiroether **8** offers only one plausible path for allylic oxidation in position 3. However, this spiroether was not converted at all and the starting material **8** was recovered almost quantitatively from the reaction mixture. This finding may reflect the increased sterical hindrance of the allylic position in **8** compared to the site of oxidation in regioisomer **7** and a less stable radical intermediate.

Similar results were obtained for oxidation of spiroethers **11** and **12** which contain two separated double bonds. The allyl ether **11** allows three plausible oxidation products **17**–**19**. However, due to our experiences with oxidations of **7**, **8** and theaspirane (**1**), position 2 should be activated most strongly for hydrogen abstraction and should thus be privileged for oxidation. As a consequence, the α,β-unsaturated lactone **17** is indeed the major product along with some unidentified more complex oxidation products. Again, oxidation in 5-position of **11** to the enone **18** was not observed. In addition, the allylic hydrogens in 9-position are obviously also of low reactivity and the corresponding oxidation product **19** was also not detectable. In agreement with the oxidation of **8**, substrate **12** was not oxidized by PSA at all and the starting material was reisolated almost quantitatively.

Computational analysis (for full computational details see Supporting Information File 1) of relative radical stabilities and bond-dissociation enthalpies (BDH_298_) based on DFT (B3LYP/6-31+G** and B3LYP/6-31G* + PCM) and composite method (CBS-QB3) calculations enable a quantification and visual rationalization of the observed experimental results. Since PCM and gas phase results do not differ significantly neither in terms of geometry nor energy, we will restrict the discussion to gas phase values only unless otherwise stated (for details of the PCM results see Supporting Information File 1). The CBS-QB3 method has been used to obtain accurate energies and to evaluate the DFT-energies in terms of relative and absolute values. For this comparison CBS-QB3 has been applied to model structures **11A** and **11B** that contain key structural features of **11** and **12** (Table 1). According to the data summarized in Table 1 and Table 2 B3LYP/6-31+G** gives reasonable results and is a suitable method to predict at least relative BDH-values of C–H bonds of the spiro compounds at hand. In absolute terms, B3LYP underestimates the BDH systematically by 2.9–4.6 kcal/mol.

**Table 1:** Model substrates **11A** and **11B** that allowed a determination of accurate BDH_298_ values with the CBS-QB3 method.

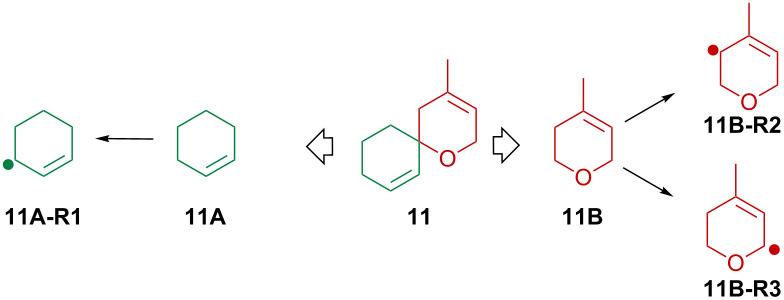			

entry	compd	CBS-QB3	

		BDH_298_ [kcal/mol]	Δ*G* [kcal/mol]

1	**11A-R1**	83.5	75.0
2	**11B-R2**	86.9	77.4
3	**11B-R3**	79.5	70.8

**Table 2:** Optimized geometries and BDH_298_ values of radicals derived from **11** and **12** for gas phase and PCM calculations.

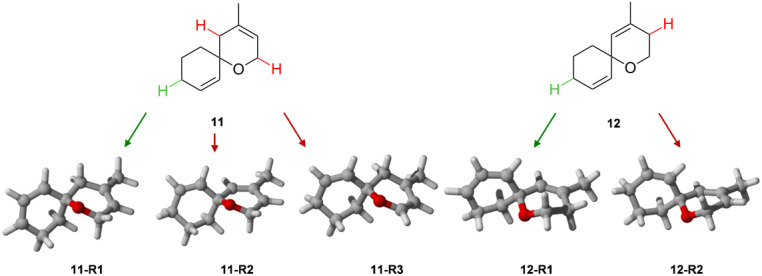							

entry	compd	B3LYP/6-31+G**			B3LYP/6-31G* + PCM		

		Δ*H*^a^ [kcal/mol]	Δ*G*^a^ [kcal/mol]	BDH_298_ [kcal/mol]	Δ*H*^a^ [kcal/mol]	Δ*G*^a^ [kcal/mol]	BDH_298_ [kcal/mol]

1	**11**	1.2	1.3	—	1.0	1.2	—
2	**11-R1**	−1.5	−0.2	80.9	−1.9	−0.3	81.6
3	**11-R2**	0.5	0.7	82.9	0.3	0.7	83.7
4	**11-R3**	−7.5	−6.4	74.9	−7.8	0.0	74.6
5	**12**	0.0	0.0	—	0.0	0.0	—
6	**12-R1**	−3.3	−2.1	80.3	3.4	2.0	80.1
7	**12-R2**	0.0	0.0	83.6	0.0	0.0	83.4

^a^Referring to **12** for closed-shell species and **12-R2** for radicals.

**Figure 3:**
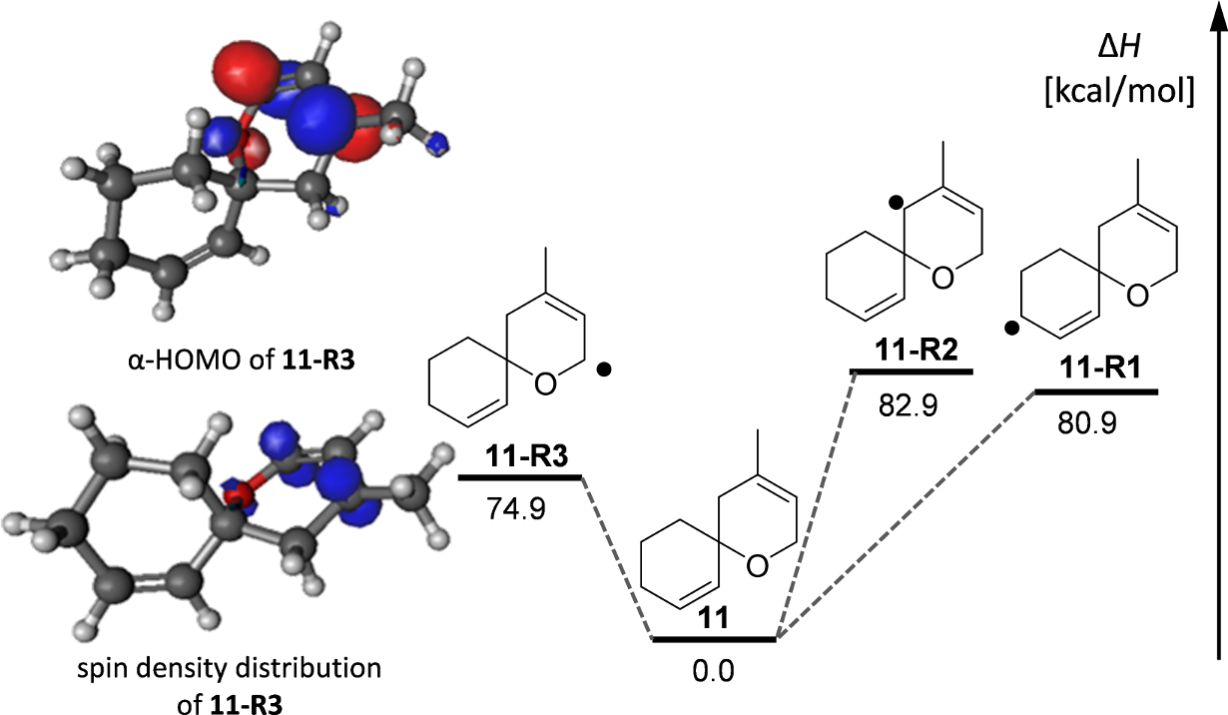
Bond-dissociation enthalpies for three allylic C–H bonds in **11**. Double stabilization of the radical in **11-R3** by the adjacent oxygen and hyperconjugation lead to a rather low BDH of 74.9 kcal/mol (B3LYP/6-31+G**).

The experimental observation as summarized in Scheme 3, e.g., the selective oxidation in 2-position of an allyl ether subunit may be rationalized by the particularly low BDH_298_ for the corresponding C–H bond compared to the other allylic C–H bonds (Table 2). Carbon-centered radicals adjacent to an oxygen atom are commonly known to be stabilized as they benefit from inductive effects as well as from orbital interactions of the p-type lone pair of the oxygen atom with the half-filled p-orbital of the mainly sp^2^ hybridized radical [40]. The picture of the SOMO and the mapped out spin density of **11-R3** illustrate the latter effect (Figure 3). In addition, the methyl group in 3-position of the allylic system in **11-R3** as well as in the model system **11B-R3** helps to stabilize the radical further, mainly by hyperconjugation. In total, both substituent effects result in a rather low C–H bond-dissociation enthalpy of 79.5 kcal/mol at CBS-QB3 level of theory (74.9 kcal/mol B3LYP/6-31+G**, Table 2).

Compared to the other potential allylic radicals like **11-R1** and **11-R2**, **11-R3** is up to 8 kcal/mol more stable. Within the subset of allylic radicals that differ only by their alkyl substitution pattern (**11-R1**, **11-R2**, **12-R1** and **12-R2**) the energetic differences are less pronounced. Equally, the reasons for the small variations in BDH_298_ values are more subtle and might be referred to additional steric strain imposed by a change in hybridization of the carbon atom from sp^3^ to sp^2^ upon radical formation and hyperconjugation effects (Table 2). Correlating the experimental observations with the computational results, one can deduce that a BHD_298_ of around 80 kcal/mol (CBS-QB3) seems to be a threshold that might be used as a guideline to decide whether an allylic oxidation of spiro compounds related to **11** and **12** with PSA takes places or not.

**Scheme 4:**
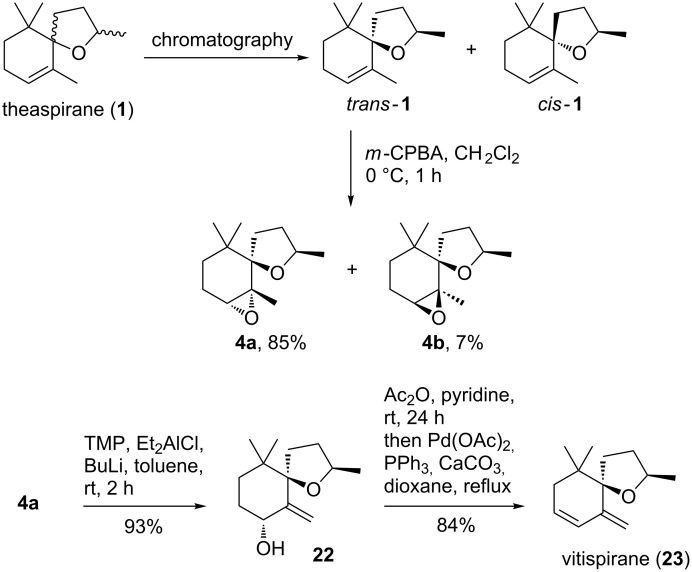
Improved 3-step synthesis of vitispirane (**23**) from theaspirane (**1**). Only one enantiomer of racemic compounds is shown.

As a further test on the substrate scope of the reaction, we were interested in the biocatalytic oxidation of vitispirane as a terpenoid with a conjugated double bond and a sterically hindered allylic position (Figure 2). Vitispirane is a flavor compound of vanilla and quince fruit and was identified in grape juice and wine [41–42]. Various syntheses of vitispirane have been reported in the literature [43–45]. To get sufficient quantity of the oxidation precursor, we focused on a strategy reported by Ohloff starting from commercially available theaspirane (**1**) [46]. As described by Ohloff and coworkers, the separation of vitispirane diastereoisomers is quite difficult. Therefore we decided to start with diastereomerically pure theaspirane (*trans-***1**) that was obtained by chromatographic separation of the commercial diastereomeric mixture of *cis*- and *trans*-theaspirane (**1**). As depicted in Scheme 4, *trans*-theaspirane (*trans*-**1**) was converted to the corresponding epoxides **4a** and **4b** following the literature protocol using *m*-chloroperbenzoic acid. The major epoxide **4a** was obtained in good yield and a diastereoselectivity of 11:1.

Isomerisation of epoxide **4a** to allyl alcohol **22** was reported with aluminium triisopropoxide at 140 °C [46]. However, in our hands this procedure gave only complex reaction mixtures containing minor amounts of the target allyl alcohol **22**. In an alternative protocol, we used Yamamoto´s conditions [47] for the isomerization of epoxides (TMP, *n*-BuLi, Et_2_AlCl) and obtained allyl alcohol **22** in excellent 93% yield. The conversion of allyl alcohol **22** to vitispirane (**23**) has been reported with POCl_3_ in pyridine. Again, this known protocol did not work satisfactory for us resulting in only minor amounts of the desired vitispirane (**23**). As a consequence, we decided to use an alternative method via acetylation and Pd-catalyzed elimination. This conversion gave vitispirane (**23**) in good yield [48]. Overall, this improved protocol gave racemic vitispirane (**23**) in three steps from theaspirane (*trans-***1**) in excellent 72% overall yield. It should be noted that the gas chromatographic separation of racemic vitispirane (**23**) has been reported by Schreier [45]. In addition, we have been able to separate racemic vitispirane by HPLC on a chiral stationary phase (see Supporting Information File 1).

**Scheme 5:**
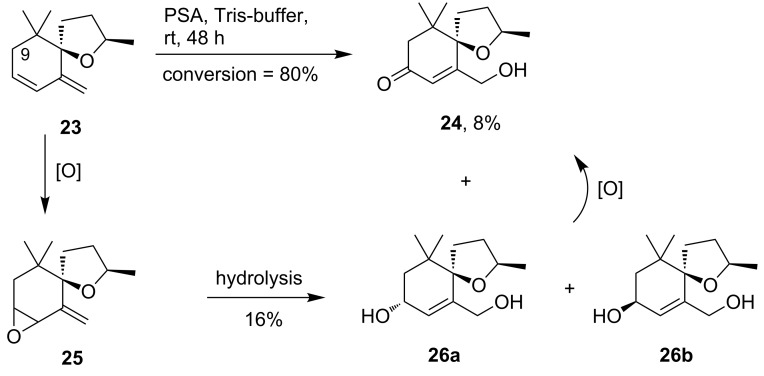
Oxidation of vitispirane (**23**) with PSA gave enone **24** and two diastereomeric allyl alcohols **26a** and **26b**. A putative intermediate is epoxide **25**, which upon hydrolysis would give allyl alcohols **26a** and **26b**. Oxidation of the latter might provide enone **24**. Only one enantiomer of racemic compounds is shown.

Vitispirane (**23**) is a challenging substrate for allylic oxidation with PSA. As outlined above, the oxidation with PSA is quite sensitive to steric and electronic factors and the allylic position 9 in vitispirane (**23**) is sterically hindered (Scheme 5).

Treatment of vitispirane (**23**) with PSA was indeed not a clean conversion giving a number of different oxidation products. From this mixture, however, three main products **24**, **26a** and **26b** were identified unambiguously. So far, we have never detected similar oxidation products with PSA and rationalize their formation by an initial epoxidation of the endocyclic double bond to give the allyl epoxide **25**, which might then be hydrolyzed to two diastereomeric alcohols **26a** and **26b**. The latter reaction is known for similar allyl epoxides under slightly acidic conditions [49]. However, participation of hydrolases from the PSA lyophilisate is also possible. A fraction of the resulting alcohols **26** would then finally be oxidized to give enone **24**. Support for an epoxide intermediate comes from oxidations of substrates containing similarly hindered allylic CH-bonds. For β-ionone, for example, we have previously observed epoxidation to be the major oxidation pathway.

It should be noted, that all three compounds **24**, **26a** and **26b** are new derivatives of vitispirane **24** with potentially interesting properties as flavors. The relative stereochemistry of **26a** and **26b** was evaluated after HPLC separation of the two diastereoisomers by 2D-NOESY NMR.

The compounds **24**, **26a** and **26b** were obtained as racemic mixtures. However, we found HPLC protocols for the separation of these terpenoids with commercial chiral stationary phases (see Supporting Information File 1). This allows the isolation of large quantities of the enantiomerically pure derivatives and their detailed olfactory analysis.

## Conclusion

The edible fungus PSA allows efficient allylic oxidations of terpenoid olefins. The oxidation protocols are quite simple, because the lyophilisate of PSA can be used as a catalyst. In this paper we have investigated the substrate scope of these biocatalytic oxidations with a special focus on spiroether derivatives due to their high relevance as flavor compounds. Several new spirocyclic model compounds and the natural product vitispirane (**23**) were synthesized and submitted to oxidation with PSA. The outcome of these oxidations was found to be dependent on steric and electronic factors of the substrate. The reactivity of most terpenoids towards allylic oxidation with PSA can thus be estimated using the same rules established for conventional radical oxidations: The reactivity is determined by bond-dissociation energies of the allylic CH-bonds. Correlating the experimental observations of this study with computational results we deduced a threshold BHD_298_ of around 80 kcal/mol as a guideline to decide whether an allylic oxidation with PSA takes places or not.

Allyl spiroethers **7** and **11** were oxidized to the corresponding α,β-unsaturated lactone derivatives **14** and **17**, whereas the close derivatives **8** and **12** containing slightly less reactive allylic C–H bonds were not converted by PSA at all. The natural product vitispirane (**23**) was oxidized by PSA, and three new vitispirane derivatives **24**, **26a** and **26b** were isolated. In this case, the oxidation pathway is not favoring products of allylic oxidation but most likely those of epoxidation with subsequent hydrolysis of the epoxide. HPLC protocols with chiral stationary phases allow the separation of racemic mixtures of oxidized vitispirane derivatives.

## Supporting Information

File 1 Computational details, experimental procedures, analytical data, NMR spectra and chromatograms of new compounds.
