# Supplementary material for: Elucidation of the regio- and chemoselectivity of enzymatic allylic oxidations with Pleurotus sapidus – conversion of selected spirocyclic terpenoids and computational analysis
Source: Beilstein J Org Chem. 2013 Oct 29;9:2233–41. doi: 10.3762/bjoc.9.262 (PMC3817473; doi:10.3762/bjoc.9.262)
Supplement: File 1 — Computational details, experimental procedures, analytical data, NMR spectra and chromatograms of new compounds. [file Beilstein_J_Org_Chem-09-2233-s001.pdf]

Supporting Information

for

Elucidation of the regio- and chemoselectivity of enzymatic allylic oxidations with *Pleurotus sapidus* – conversion of selected spirocyclic terpenoids and computational analysis

Verena Weidmann[1], Mathias Schaffrath[2], Holger Zorn[3], Julia Rehbein*[4] and Wolfgang Maison*[1]

Address: [1]Department of Chemistry, University of Hamburg, Bundesstr. 45, 20146 Hamburg, Germany, [2]LGCR Chemistry, Sanofi-Aventis Deutschland GmbH, 65926 Frankfurt am Main, Germany, [3]Justus-Liebig-University Giessen, Institute of Food Chemistry and Food Biotechnology, Heinrich-Buff-Ring 58, 35392 Gießen and [4]Department of Chemistry, University of Hamburg, Martin-Luther-King-Platz 6, 20146 Hamburg, Germany

Email: Wolfgang Maison* - maison@chemie.uni-hamburg.de, Julia Rehbein* - rehbein@chemie.uni-hamburg.de

*Corresponding author

Computational details, experimental procedures, analytical data, NMR spectra and chromatograms of new compounds.

Table of Contents

Computational details S2–27
Experimental procedures and analytical data for compounds **4-26** S28–35
NMR spectra of compounds **4-26** S36–49
HPLC analysis of compounds **1**, **2**, **4a**, **14**, **17**, **22**, **26a** and **26b** S50–62
References S63

Computational details

All calculations have been conducted using the Gaussian09 suite of programs [1-5]. Density functional theory calculations were initially carried out at the B3LYP/6-31+G** level of theory [6,7], as this level has previously provided adequate results in the calculation of BDEs of not highly activated bonds, e.g., strong bonds. Nevertheless, it has also been reported that the popular hybrid density functional B3LYP has (also) limitations concerning prediction of BDE values of weaker bonds. Therefore, a CBS-QB3 calculation [8-13] has been applied to smaller model systems (Table 1) of the allylic substructures in the compounds of interest to obtain accurate thermodynamic properties. Solvent effects were modeled using the implemented polarized continuum model (PCM) [14-16] applied as full optimizations and frequency analysis in the dielectric field (B3LYP/6-31G* + PCM) for substrate and product structures. As a rough model for the Tris-buffer used in the experiments we have chosen water as the solvent. Stationary points were confirmed as ground states by calculated harmonic vibrational frequencies [17]. Approximate free energies were obtained through thermochemical analysis of the frequency calculations, using the thermal correction to the Gibbs free energy as reported in Gaussian09. This takes into account zero-point effects, thermal enthalpy corrections, and entropy. All energies reported in this paper are enthalpies in kcal mol^{-1} at 298 K if not stated otherwise. Frequencies remained unscaled. Population analysis on B3LYP/6-31+G** level of theory has been done with the NBO program as implemented in Gaussian09 [18].

Results obtained at B3LYP/6-31+G** level of theory

Table 1   DFT results

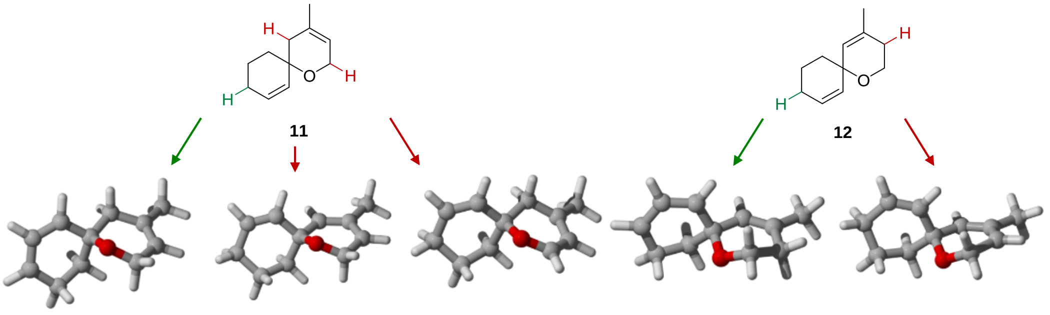

11-R1 11-R2 11-R3 12-R1 12-R2

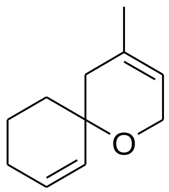	**11**	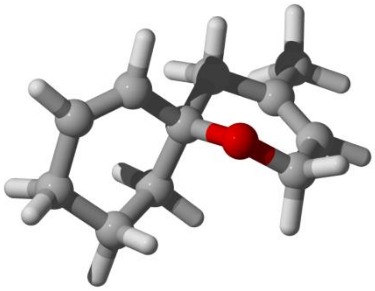

xyz-matrix

28

XYZ file generated by gabedit : coordinates in Angstrom

C	-2.1978710000	0.8769460000	0.9158210000
C	-3.2153830000	-0.1261930000	0.3554970000
C	-2.6770120000	-0.8397140000	-0.8584440000
C	-1.3877530000	-0.8313870000	-1.2168990000
C	-0.2917240000	-0.1404450000	-0.4332200000
C	-0.8011330000	0.2477120000	0.9732410000
O	0.0360540000	1.0362310000	-1.2176400000
C	1.1071200000	1.8352620000	-0.7235890000
C	2.2687960000	1.0443630000	-0.1935040000
C	2.2216250000	-0.2815440000	-0.0157030000
C	0.9600890000	-1.0427690000	-0.3620040000
C	3.3877580000	-1.0875170000	0.4921460000
H	-2.5051350000	1.2153290000	1.9121800000
H	-2.1661040000	1.7614740000	0.2682110000
H	-4.1547530000	0.3792260000	0.0959250000
H	-3.4814610000	-0.8685080000	1.1250530000
H	-3.3921210000	-1.3833710000	-1.4745470000
H	-1.0739330000	-1.3328120000	-2.1305860000
H	-0.8374680000	-0.6609720000	1.5892240000

H	-0.0869980000	0.9205950000	1.4583130000
H	0.7403730000	2.5332600000	0.0507770000
H	1.4202660000	2.4525770000	-1.5745710000
H	3.1668170000	1.6081900000	0.0560980000
H	1.0996230000	-1.5406830000	-1.3326010000
H	0.7911800000	-1.8456230000	0.3676840000
H	3.6700440000	-1.8668190000	-0.2286520000
H	3.1338580000	-1.6031830000	1.4278740000
H	4.2652530000	-0.4596370000	0.6739200000

thermodynamic data

Zero-point correction= 0.247266 (Hartree/Particle)
Thermal correction to Energy= 0.258291
Thermal correction to Enthalpy= 0.259235
Thermal correction to Gibbs Free Energy= 0.211116
Sum of electronic and zero-point Energies= -503.784513
Sum of electronic and thermal Energies= -503.773489
Sum of electronic and thermal Enthalpies= -503.772544
Sum of electronic and thermal Free Energies= -503.820663

	E (Thermal)	CV	S
	KCal/Mol	Cal/Mol-Kelvin	Cal/Mol-Kelvin
Total	162.080	44.664	101.275

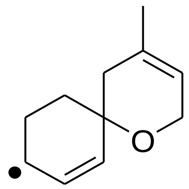	**11-R1**	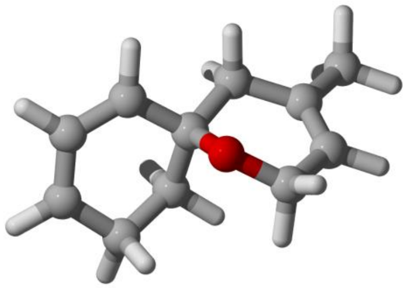

xyz-matrix

27
XYZ file generated by gabedit : coordinates in Angstrom

C	-2.2891160000	0.8946110000	0.9394210000
C	-3.2801610000	0.0282260000	0.2191770000
C	-2.8473980000	-0.9080070000	-0.7123570000
C	-1.5040530000	-1.0861140000	-1.0236970000
C	-0.4178750000	-0.2755980000	-0.3609330000
C	-0.9100090000	0.2194560000	1.0185660000
O	-0.1807960000	0.8460800000	-1.2591870000
C	0.8503110000	1.7460510000	-0.8620020000
C	2.0752480000	1.0694900000	-0.3164710000
C	2.1123050000	-0.2363040000	-0.0241560000
C	0.8849180000	-1.0939050000	-0.2457890000

C	3.3426950000	-0.9292280000	0.4988530000
H	-2.6475830000	1.1360270000	1.9478150000
H	-2.1977570000	1.8572160000	0.4107680000
H	-4.3422680000	0.1654360000	0.4029620000
H	-3.5866040000	-1.5142280000	-1.2323250000
H	-1.2099950000	-1.7806020000	-1.8059110000
H	-0.9802500000	-0.6529060000	1.6801530000
H	-0.1741540000	0.8950000000	1.4660470000
H	0.4631480000	2.4768680000	-0.1287640000
H	1.0977940000	2.3147470000	-1.7666910000
H	2.9484320000	1.7006230000	-0.1558570000
H	1.0207200000	-1.6811560000	-1.1656870000
H	0.7854140000	-1.8253090000	0.5675590000
H	3.6422420000	-1.7519870000	-0.1644240000
H	3.1566020000	-1.3740840000	1.4854480000
H	4.1881770000	-0.2403950000	0.5874870000

thermodynamic data

Zero-point correction= 0.233624 (Hartree/Particle)
Thermal correction to Energy= 0.244624
Thermal correction to Enthalpy= 0.245568
Thermal correction to Gibbs Free Energy= 0.196949
Sum of electronic and zero-point Energies= -503.157689
Sum of electronic and thermal Energies= -503.146689
Sum of electronic and thermal Enthalpies= -503.145745
Sum of electronic and thermal Free Energies= -503.194364

	E (Thermal)	CV	S
	KCal/Mol	Cal/Mol-Kelvin	Cal/Mol-Kelvin
Total	153.504	44.670	102.327

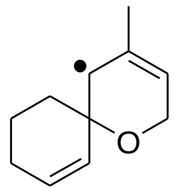	**11-R2**	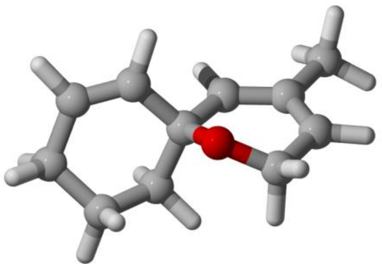

xyz-matrix

27
XYZ file generated by gabedit : coordinates in Angstrom

C	-2.4213050000	0.8355010000	0.7379220000
C	-3.2416250000	-0.2948740000	0.1018640000
C	-2.4669550000	-0.9913550000	-0.9875850000
C	-1.1440680000	-0.8755800000	-1.1538410000
C	-0.2397510000	-0.0653380000	-0.2417520000
C	-0.9982500000	0.3559600000	1.0451930000
O	0.1201650000	1.0966870000	-1.0288540000

C	1.2574410000	1.8360280000	-0.5876290000
C	2.4475360000	0.9727770000	-0.3086990000
C	2.2769750000	-0.3668990000	0.0289920000
C	0.9858460000	-0.8792680000	0.1055440000
C	3.4742870000	-1.2550480000	0.3005520000
H	-2.9077490000	1.1931540000	1.6529540000
H	-2.3690610000	1.6824210000	0.0434720000
H	-4.1816290000	0.0947890000	-0.3099190000
H	-3.5356290000	-1.0292400000	0.8688270000
H	-3.0313920000	-1.6131960000	-1.6813010000
H	-0.6479870000	-1.3677390000	-1.9871960000
H	-1.0372690000	-0.5063360000	1.7244580000
H	-0.4201560000	1.1307280000	1.5609550000
H	1.0011350000	2.4372270000	0.3058080000
H	1.4670080000	2.5488200000	-1.3930190000
H	3.4352120000	1.4260820000	-0.3318950000
H	0.8156370000	-1.9008940000	0.4361110000
H	3.2176340000	-2.0714770000	0.9826030000
H	4.2986030000	-0.6879830000	0.7445410000
H	3.8453430000	-1.7049510000	-0.6281060000

thermodynamic data

Zero-point correction= 0.233171 (Hartree/Particle)
Thermal correction to Energy= 0.244558
Thermal correction to Enthalpy= 0.245502
Thermal correction to Gibbs Free Energy= 0.195131
Sum of electronic and zero-point Energies= -503.154862
Sum of electronic and thermal Energies= -503.143474
Sum of electronic and thermal Enthalpies= -503.142530
Sum of electronic and thermal Free Energies= -503.192901

	E (Thermal)	CV	S
	KCal/Mol	Cal/Mol-Kelvin	Cal/Mol-Kelvin
Total	153.463	44.981	106.015

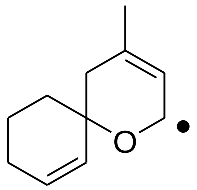	**11-R3**	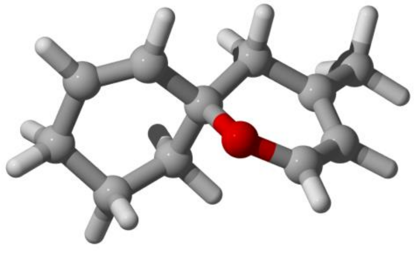

xyz-matrix

27
XYZ file generated by gabedit : coordinates in Angstrom

C	-2.1348250000	0.7613510000	1.0863610000
C	-3.1571460000	-0.1314270000	0.3701710000
C	-2.6508070000	-0.5814380000	-0.9759510000
C	-1.3712340000	-0.4927400000	-1.3581440000

C	-0.2607900000	0.0227650000	-0.4738350000
C	-0.7280770000	0.1601810000	0.9894400000
O	0.0464960000	1.3667970000	-0.9931800000
C	1.2361650000	1.9157060000	-0.6232140000
C	2.2957770000	1.1692870000	-0.1278860000
C	2.2321620000	-0.2174180000	-0.0445590000
C	0.9931040000	-0.8722170000	-0.5984200000
C	3.3595380000	-1.0732760000	0.4453370000
H	-2.4164420000	0.8974570000	2.1367550000
H	-2.1308000000	1.7544870000	0.6217260000
H	-4.1112880000	0.3964780000	0.2441120000
H	-3.3878320000	-1.0168780000	0.9839390000
H	-3.3798320000	-0.9977830000	-1.6698420000
H	-1.0777690000	-0.8040150000	-2.3589800000
H	-0.7237330000	-0.8389700000	1.4460210000
H	-0.0030530000	0.7660700000	1.5411170000
H	1.2649610000	2.9870800000	-0.7835230000
H	3.1898170000	1.7051930000	0.1806330000
H	1.1461720000	-1.1143380000	-1.6636940000
H	0.7885990000	-1.8277610000	-0.0989780000
H	3.0477980000	-1.7158840000	1.2820760000
H	4.2064990000	-0.4680700000	0.7839390000
H	3.7265420000	-1.7506420000	-0.3414190000

thermodynamic data

Zero-point correction= 0.233807 (Hartree/Particle)
Thermal correction to Energy= 0.244924
Thermal correction to Enthalpy= 0.245869
Thermal correction to Gibbs Free Energy= 0.196827
Sum of electronic and zero-point Energies= -503.167329
Sum of electronic and thermal Energies= -503.156211
Sum of electronic and thermal Enthalpies= -503.155267
Sum of electronic and thermal Free Energies= -503.204308

	E (Thermal)	CV	S
	KCal/Mol	Cal/Mol-Kelvin	Cal/Mol-Kelvin
Total	153.692	44.680	103.216

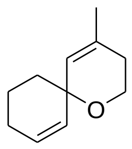	**12**	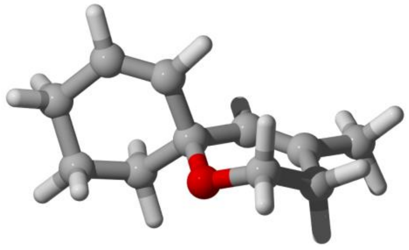

xyz-matrix
28
XYZ file generated by gabedit : coordinates in Angstrom

C	2.6320210000	0.1230680000	-1.1259420000
C	3.3384260000	-0.2421200000	0.1869260000
C	2.4171650000	-0.1116650000	1.3726180000
C	1.0861780000	0.0046700000	1.2765840000
C	0.3270960000	-0.0029510000	-0.0465860000
C	1.2376650000	-0.5119490000	-1.1829450000
O	-0.0296200000	1.3513170000	-0.4212660000
C	-1.1792130000	1.8694850000	0.2400690000
C	-2.4279840000	1.0681580000	-0.1237820000
C	-2.1673000000	-0.4156100000	0.0112700000
C	-0.9084620000	-0.8736530000	0.0634310000
C	-3.3687610000	-1.3219670000	0.0717950000
H	3.2314100000	-0.2030290000	-1.9839360000
H	2.5288740000	1.2118010000	-1.1976260000
H	4.2225050000	0.3908770000	0.3391010000
H	3.7199500000	-1.2749340000	0.1405190000
H	2.8765970000	-0.1138860000	2.3606630000
H	0.4896700000	0.1069820000	2.1806900000
H	1.3277490000	-1.6030750000	-1.0967510000
H	0.7409150000	-0.2971050000	-2.1344160000
H	-1.2607780000	2.9115460000	-0.0819980000
H	-1.0323760000	1.8641340000	1.3309370000
H	-2.7416630000	1.2987490000	-1.1523620000
H	-3.2616840000	1.3652250000	0.5283370000
H	-0.7202640000	-1.9391960000	0.1882560000
H	-3.0798870000	-2.3750450000	0.1396710000
H	-3.9972190000	-1.1975450000	-0.8203640000
H	-4.0010080000	-1.0822700000	0.9371050000

thermodynamic data

Zero-point correction= 0.247452 (Hartree/Particle)
Thermal correction to Energy= 0.258529
Thermal correction to Enthalpy= 0.259473
Thermal correction to Gibbs Free Energy= 0.211110
Sum of electronic and zero-point Energies= -503.786426
Sum of electronic and thermal Energies= -503.775349
Sum of electronic and thermal Enthalpies= -503.774405
Sum of electronic and thermal Free Energies= -503.822768

	E (Thermal)	CV	S
	KCal/Mol	Cal/Mol-Kelvin	Cal/Mol-Kelvin
Total	162.229	44.665	101.788

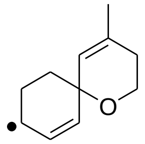	**12-R1**	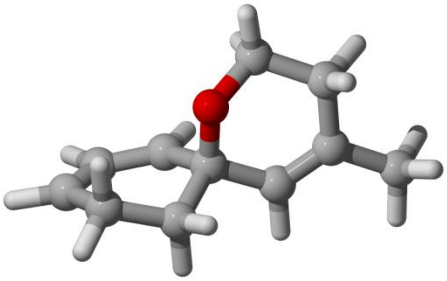

xyz-matrix

27
XYZ file generated by gabedit : coordinates in Angstrom

C	2.7460770000	-0.1312330000	-1.1370360000
C	3.3759040000	-0.0303550000	0.2208050000
C	2.5871520000	0.0188220000	1.3630070000
C	1.1955400000	-0.0173440000	1.3151770000
C	0.4495730000	-0.1124090000	-0.0042770000
C	1.3521250000	-0.7762810000	-1.0645580000
O	0.1802190000	1.2182090000	-0.5236880000
C	-0.9340270000	1.8685810000	0.0802700000
C	-2.2302450000	1.1145630000	-0.2130150000
C	-2.0632520000	-0.3654440000	0.0512770000
C	-0.8360670000	-0.8953020000	0.1561130000
C	-3.3197810000	-1.1870970000	0.1716460000
H	3.3884920000	-0.7023360000	-1.8189220000
H	2.6550190000	0.8756830000	-1.5734200000
H	4.4573530000	0.0385050000	0.3027170000
H	3.0722860000	0.1025820000	2.3338960000
H	0.6168700000	0.0527060000	2.2315430000
H	1.4602030000	-1.8360750000	-0.8027680000
H	0.8462860000	-0.7212190000	-2.0335240000
H	-0.9516760000	2.8783600000	-0.3395100000
H	-0.7839130000	1.9564720000	1.1669580000
H	-2.5325580000	1.2713650000	-1.2588200000
H	-3.0425830000	1.5190360000	0.4075670000
H	-0.7158040000	-1.9570720000	0.3677170000
H	-3.0983260000	-2.2467380000	0.3301620000
H	-3.9327660000	-1.0985940000	-0.7354510000
H	-3.9421060000	-0.8373780000	1.0061420000

thermodynamic data

Zero-point correction= 0.233799 (Hartree/Particle)
Thermal correction to Energy= 0.244858
Thermal correction to Enthalpy= 0.245803
Thermal correction to Gibbs Free Energy= 0.196913
Sum of electronic and zero-point Energies= -503.160545
Sum of electronic and thermal Energies= -503.149485
Sum of electronic and thermal Enthalpies= -503.148541
Sum of electronic and thermal Free Energies= -503.197431

E (Thermal)	CV	S
KCal/Mol	Cal/Mol-Kelvin	Cal/Mol-Kelvin

Total	153.651	44.674	102.897

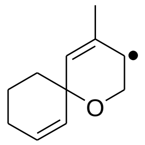	**12-R2**	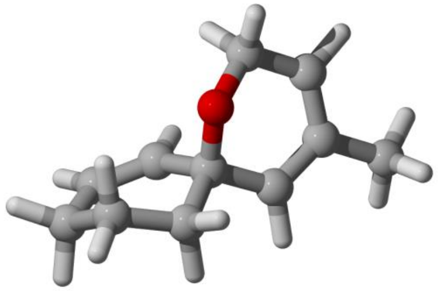

xyz-matrix

27
XYZ file generated by gabedit : coordinates in Angstrom

C	2.6259590000	0.0731780000	-1.0318610000
C	3.1937190000	-0.2166740000	0.3648300000
C	2.1563410000	-0.0246000000	1.4408420000
C	0.8420960000	0.0825330000	1.2017590000
C	0.2230650000	-0.0113210000	-0.1895410000
C	1.2439910000	-0.5685140000	-1.1985960000
O	-0.1176710000	1.3105100000	-0.6860340000
C	-1.2007350000	1.9548990000	-0.0255410000
C	-2.4007390000	1.0701450000	0.1281700000
C	-2.2848480000	-0.3116830000	0.0285920000
C	-1.0174030000	-0.8701940000	-0.1371790000
C	-3.5126710000	-1.1979850000	0.0865720000
H	3.3096650000	-0.2973100000	-1.8044110000
H	2.5334420000	1.1559220000	-1.1739970000
H	4.0592450000	0.4264350000	0.5715740000
H	3.5755880000	-1.2492330000	0.4146370000
H	2.5109690000	0.0256280000	2.4699370000
H	0.1534310000	0.2279270000	2.0313600000
H	1.3221000000	-1.6536810000	-1.0491470000
H	0.8483850000	-0.4018040000	-2.2055590000
H	-1.4394620000	2.8272510000	-0.6454730000
H	-0.8761870000	2.3468430000	0.9567230000
H	-3.3609130000	1.5386120000	0.3297040000
H	-0.8863920000	-1.9488570000	-0.1675430000
H	-3.2965250000	-2.1469860000	0.5874820000
H	-3.8738870000	-1.4321440000	-0.9219710000
H	-4.3305520000	-0.7089060000	0.6246730000

thermodynamic data

Zero-point correction= 0.233222 (Hartree/Particle)
Thermal correction to Energy= 0.244635
Thermal correction to Enthalpy= 0.245579
Thermal correction to Gibbs Free Energy= 0.194828
Sum of electronic and zero-point Energies= -503.155675
Sum of electronic and thermal Energies= -503.144262

Sum of electronic and thermal Enthalpies= -503.143318
Sum of electronic and thermal Free Energies= -503.194069

	E (Thermal)	CV	S
	KCal/Mol	Cal/Mol-Kelvin	Cal/Mol-Kelvin
Total	153.511	44.947	106.815

	H (hydrogen atom)	

xyz-matrix

--

thermodynamic data

Zero-point correction= 0.000000 (Hartree/Particle)
Thermal correction to Energy= 0.001416
Thermal correction to Enthalpy= 0.002360
Thermal correction to Gibbs Free Energy= -0.010654
Sum of electronic and zero-point Energies= -0.500273
Sum of electronic and thermal Energies= -0.498857
Sum of electronic and thermal Enthalpies= -0.497912
Sum of electronic and thermal Free Energies= -0.510927

	E (Thermal)	CV	S
	KCal/Mol	Cal/Mol-Kelvin	Cal/Mol-Kelvin
Total	0.889	2.981	27.39

Population Analysis: NBO: α-HOMO (equals SOMO[19]) with energies and spin density distribution

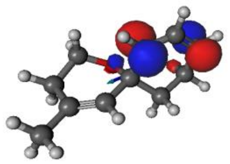	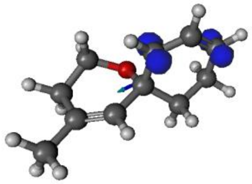	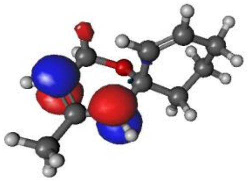	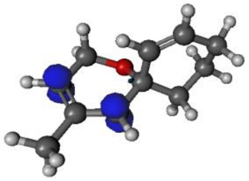		
**12-R1** α-HOMO<br>–0.18496	**12-R1** spin density	**12-R2** α-HOMO<br>–0.184580	**12-R2** α-HOMO<br>spin density		
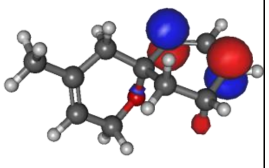	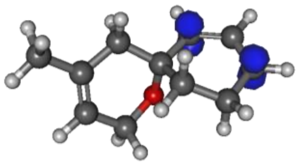	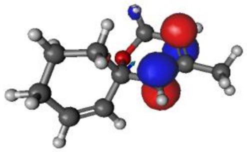	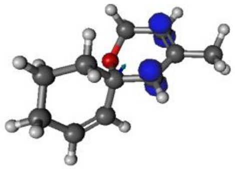	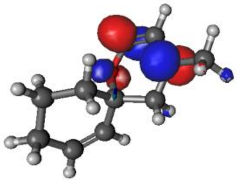	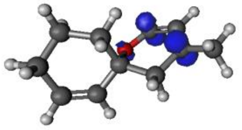
**11-R1**<br>α-HOMO	**11-R1** α-HOMO<br>spin density	**11-R2** α-HOMO<br>–0.185170	**11-R2** α-HOMO<br>spin density	**11-R3** α-HOMO<br>–0.154760	**11-R3** α-HOMO<br>spin density

Plotted β-LUMOs are similar in shape to α-HOMOs therefore, we used the α-HOMOs for further discussions of shape and energy of the SOMO.

1. **Results obtained at B3LYP/6-31G* PCM(solvent=water) level of theory**

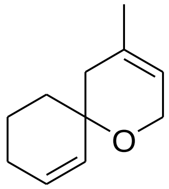	**11**	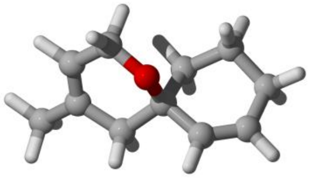

xyz-matrix

28
XYZ file generated by gabedit : coordinates in Angstrom

C	-2.1843060000	0.8851390000	0.9105740000
C	-3.2112880000	-0.1127410000	0.3591600000
C	-2.6801610000	-0.8447500000	-0.8467240000
C	-1.3928090000	-0.8494620000	-1.2076640000
C	-0.2916420000	-0.1512940000	-0.4357150000
C	-0.7935170000	0.2442250000	0.9700700000
O	0.0286190000	1.0226990000	-1.2309220000
C	1.0956010000	1.8286080000	-0.7360880000
C	2.2593080000	1.0451010000	-0.1997900000
C	2.2192090000	-0.2791580000	-0.0193770000
C	0.9627170000	-1.0486960000	-0.3676350000
C	3.3866860000	-1.0753890000	0.4996230000
H	-2.4882400000	1.2353100000	1.9038750000
H	-2.1437870000	1.7652670000	0.2564570000
H	-4.1477400000	0.3976050000	0.0965900000
H	-3.4836480000	-0.8451180000	1.1361760000
H	-3.4019090000	-1.3960190000	-1.4491810000
H	-1.0817240000	-1.3762850000	-2.1090950000
H	-0.8369390000	-0.6623480000	1.5887480000
H	-0.0730200000	0.9133500000	1.4517110000
H	0.7271430000	2.5301000000	0.0335750000
H	1.4107700000	2.4470620000	-1.5870410000
H	3.1504960000	1.6179820000	0.0540580000
H	1.1041060000	-1.5475790000	-1.3383870000
H	0.7984050000	-1.8541540000	0.3605390000
H	3.6783580000	-1.8586220000	-0.2141380000
H	3.1293150000	-1.5894370000	1.4360100000
H	4.2600100000	-0.4414070000	0.6845910000

thermodynamic data

Zero-point correction= 0.248546 (Hartree/Particle)
Thermal correction to Energy= 0.259530
Thermal correction to Enthalpy= 0.260474
Thermal correction to Gibbs Free Energy= 0.212434
Sum of electronic and zero-point Energies= -503.748051
Sum of electronic and thermal Energies= -503.737066
Sum of electronic and thermal Enthalpies= -503.736122
Sum of electronic and thermal Free Energies= -503.784163

	E (Thermal)	CV	S
	KCal/Mol	Cal/Mol-Kelvin	Cal/Mol-Kelvin
Total	162.857	44.404	101.109

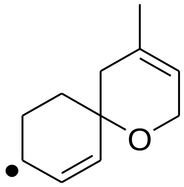	**11-R1**	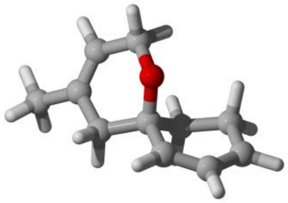

xyz-matrix

27
XYZ file generated by gabedit : coordinates in Angstrom

C	-2.2736380000	0.9042830000	0.9342360000
C	-3.2724690000	0.0452570000	0.2149940000
C	-2.8496110000	-0.9102810000	-0.7001480000
C	-1.5081790000	-1.1091780000	-1.0057850000
C	-0.4173810000	-0.2899380000	-0.3601180000
C	-0.9014650000	0.2159490000	1.0170240000
O	-0.1920100000	0.8264370000	-1.2711020000
C	0.8328380000	1.7362000000	-0.8754500000
C	2.0624170000	1.0707110000	-0.3267410000
C	2.1096400000	-0.2323530000	-0.0303920000
C	0.8890530000	-1.1009940000	-0.2492680000
C	3.3435600000	-0.9113000000	0.5016900000
H	-2.6320130000	1.1514140000	1.9415450000
H	-2.1703690000	1.8680190000	0.4087470000
H	-4.3332690000	0.1972620000	0.3964590000
H	-3.5952600000	-1.5196440000	-1.2087220000
H	-1.2193170000	-1.8358800000	-1.7609990000
H	-0.9783970000	-0.6522630000	1.6835790000
H	-0.1593090000	0.8878040000	1.4599990000
H	0.4425610000	2.4686290000	-0.1464070000
H	1.0788110000	2.3067150000	-1.7808410000
H	2.9275850000	1.7126880000	-0.1651150000
H	1.0259020000	-1.6908500000	-1.1682970000
H	0.7972760000	-1.8335150000	0.5642180000
H	3.6522210000	-1.7385280000	-0.1528560000
H	3.1568540000	-1.3516800000	1.4908540000
H	4.1839740000	-0.2149740000	0.5889050000

thermodynamic data

Zero-point correction= 0.234791 (Hartree/Particle)
Thermal correction to Energy= 0.245747
Thermal correction to Enthalpy= 0.246691
Thermal correction to Gibbs Free Energy= 0.198175
Sum of electronic and zero-point Energies= -503.121776
Sum of electronic and thermal Energies= -503.110820
Sum of electronic and thermal Enthalpies= -503.109876

Sum of electronic and thermal Free Energies= -503.158392

	E (Thermal)	CV	S
	KCal/Mol	Cal/Mol-Kelvin	Cal/Mol-Kelvin
Total	154.209	44.430	102.110

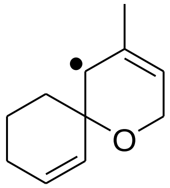	**11-R2**	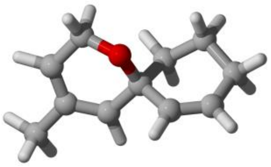

xyz-matrix

27
XYZ file generated by gabedit : coordinates in Angstrom

C	-2.4145420000	0.8406210000	0.7320800000
C	-3.2413440000	-0.2865390000	0.0998270000
C	-2.4686150000	-0.9972340000	-0.9812920000
C	-1.1460820000	-0.8895100000	-1.1459440000
C	-0.2394580000	-0.0723150000	-0.2411950000
C	-0.9955030000	0.3535600000	1.0443840000
O	0.1144500000	1.0879050000	-1.0364610000
C	1.2488880000	1.8330980000	-0.5910530000
C	2.4432260000	0.9741340000	-0.3159590000
C	2.2777170000	-0.3640380000	0.0243220000
C	0.9890520000	-0.8811600000	0.1048900000
C	3.4774930000	-1.2471900000	0.3011330000
H	-2.9018010000	1.2084880000	1.6425970000
H	-2.3528990000	1.6841080000	0.0331530000
H	-4.1786930000	0.1062670000	-0.3162820000
H	-3.5430480000	-1.0136470000	0.8708130000
H	-3.0369910000	-1.6274080000	-1.6650540000
H	-0.6496180000	-1.4034970000	-1.9672060000
H	-1.0418450000	-0.5079570000	1.7243490000
H	-0.4151500000	1.1268360000	1.5608580000
H	0.9912390000	2.4313880000	0.3030580000
H	1.4568630000	2.5522710000	-1.3922820000
H	3.4276900000	1.4341060000	-0.3357300000
H	0.8231410000	-1.9015560000	0.4417560000
H	3.2306060000	-2.0423020000	1.0124540000
H	4.3121590000	-0.6688570000	0.7110660000
H	3.8330750000	-1.7295800000	-0.6182930000

thermodynamic data

Zero-point correction= 0.234294 (Hartree/Particle)
Thermal correction to Energy= 0.245645
Thermal correction to Enthalpy= 0.246590
Thermal correction to Gibbs Free Energy= 0.196309
Sum of electronic and zero-point Energies= -503.118777

Sum of electronic and thermal Energies= -503.107426
Sum of electronic and thermal Enthalpies= -503.106482
Sum of electronic and thermal Free Energies= -503.156763

	E (Thermal)	CV	S
	KCal/Mol	Cal/Mol-Kelvin	Cal/Mol-Kelvin
Total	154.145	44.765	105.825

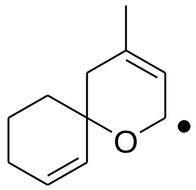	**11-R3**	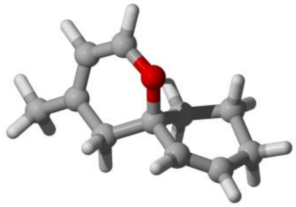

xyz-matrix

27
XYZ file generated by gabedit : coordinates in Angstrom

C	-2.1262160000	0.7701550000	1.0810640000
C	-3.1553380000	-0.1195030000	0.3715990000
C	-2.6521060000	-0.5877170000	-0.9691080000
C	-1.3737560000	-0.5100620000	-1.3535920000
C	-0.2610370000	0.0139620000	-0.4766070000
C	-0.7244310000	0.1579820000	0.9862230000
O	0.0387880000	1.3586010000	-1.0047900000
C	1.2300570000	1.9107720000	-0.6368920000
C	2.2916710000	1.1709290000	-0.1402070000
C	2.2333650000	-0.2150680000	-0.0523050000
C	0.9952310000	-0.8769720000	-0.6011020000
C	3.3587460000	-1.0633960000	0.4549020000
H	-2.4067580000	0.9162480000	2.1304850000
H	-2.1146290000	1.7608410000	0.6096890000
H	-4.1061750000	0.4141960000	0.2412700000
H	-3.3936840000	-0.9971680000	0.9936770000
H	-3.3854600000	-1.0134600000	-1.6533870000
H	-1.0799170000	-0.8457900000	-2.3472300000
H	-0.7280490000	-0.8397060000	1.4458630000
H	0.0055670000	0.7601230000	1.5364270000
H	1.2525690000	2.9840350000	-0.7912120000
H	3.1839640000	1.7107460000	0.1687100000
H	1.1453290000	-1.1252600000	-1.6657400000
H	0.7958380000	-1.8315220000	-0.0972680000
H	3.0431620000	-1.6929110000	1.3009120000
H	4.2061410000	-0.4534810000	0.7864340000
H	3.7271320000	-1.7565670000	-0.3178200000

thermodynamic data

Zero-point correction= 0.234903 (Hartree/Particle)

Thermal correction to Energy= 0.245981
Thermal correction to Enthalpy= 0.246925
Thermal correction to Gibbs Free Energy= 0.197979
Sum of electronic and zero-point Energies= -503.131411
Sum of electronic and thermal Energies= -503.120334
Sum of electronic and thermal Enthalpies= -503.119390
Sum of electronic and thermal Free Energies= -503.168336

	E (Thermal) KCal/Mol	CV Cal/Mol-Kelvin	S Cal/Mol-Kelvin
Total	154.355	44.474	103.015

	**H (hydrogen atom)**	

thermodynamic data

Zero-point correction= 0.000000 (Hartree/Particle)
Thermal correction to Energy= 0.001416
Thermal correction to Enthalpy= 0.002360
Thermal correction to Gibbs Free Energy= -0.010654
Sum of electronic and zero-point Energies= -0.500282
Sum of electronic and thermal Energies= -0.498865
Sum of electronic and thermal Enthalpies= -0.497921
Sum of electronic and thermal Free Energies= -0.510936

	E (Thermal) KCal/Mol	CV Cal/Mol-Kelvin	S Cal/Mol-Kelvin
Total	0.889	2.981	27.392

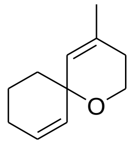	**12**	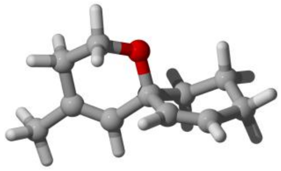

xyz-matrix

28
XYZ file generated by gabedit : coordinates in Angstrom

C	2.6344270000	0.1232160000	-1.1240760000
C	3.3364930000	-0.2488380000	0.1885990000
C	2.4146830000	-0.1079350000	1.3726740000
C	1.0857950000	0.0143710000	1.2745990000
C	0.3266620000	0.0034070000	-0.0479910000
C	1.2378570000	-0.5061660000	-1.1842570000
O	-0.0315460000	1.3576160000	-0.4233560000
C	-1.1848280000	1.8675890000	0.2423320000
C	-2.4300740000	1.0658150000	-0.1290790000
C	-2.1652080000	-0.4170470000	0.0086390000

C	-0.9063030000	-0.8704660000	0.0621390000
C	-3.3626070000	-1.3276400000	0.0750810000
H	3.2347320000	-0.2003470000	-1.9825240000
H	2.5371100000	1.2136540000	-1.1910110000
H	4.2284220000	0.3738010000	0.3404270000
H	3.7073360000	-1.2855030000	0.1402600000
H	2.8761030000	-0.1082450000	2.3602690000
H	0.4865090000	0.1224690000	2.1769740000
H	1.3254640000	-1.5978060000	-1.1025630000
H	0.7431530000	-0.2900480000	-2.1375750000
H	-1.2719060000	2.9126810000	-0.0703610000
H	-1.0405810000	1.8543170000	1.3329420000
H	-2.7394160000	1.2973440000	-1.1591580000
H	-3.2680880000	1.3636960000	0.5165730000
H	-0.7132950000	-1.9354150000	0.1886040000
H	-3.0700940000	-2.3802350000	0.1468760000
H	-3.9957050000	-1.2087390000	-0.8153300000
H	-3.9951020000	-1.0855480000	0.9402950000

thermodynamic data

Zero-point correction= 0.248741 (Hartree/Particle)
Thermal correction to Energy= 0.259782
Thermal correction to Enthalpy= 0.260726
Thermal correction to Gibbs Free Energy= 0.212411
Sum of electronic and zero-point Energies= -503.749751
Sum of electronic and thermal Energies= -503.738709
Sum of electronic and thermal Enthalpies= -503.737765
Sum of electronic and thermal Free Energies= -503.786081

	E (Thermal)	CV	S
	KCal/Mol	Cal/Mol-Kelvin	Cal/Mol-Kelvin
Total	163.016	44.407	101.689

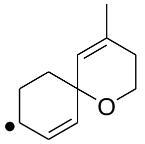	**12-R1**	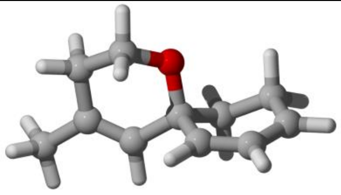

xyz-matrix

27
XYZ file generated by gabedit : coordinates in Angstrom

C	2.7487470000	-0.1407350000	-1.1365620000
C	3.3758020000	-0.0297380000	0.2225070000
C	2.5861670000	0.0296480000	1.3623300000
C	1.1950790000	-0.0040940000	1.3141990000
C	0.4487050000	-0.1071010000	-0.0039690000

C	1.3511100000	-0.7778810000	-1.0607270000
O	0.1795920000	1.2211710000	-0.5301810000
C	-0.9383400000	1.8672280000	0.0759330000
C	-2.2316590000	1.1124180000	-0.2237180000
C	-2.0622780000	-0.3656750000	0.0499510000
C	-0.8354270000	-0.8913740000	0.1603910000
C	-3.3161480000	-1.1892640000	0.1791040000
H	3.3897850000	-0.7223310000	-1.8112060000
H	2.6675510000	0.8609690000	-1.5885260000
H	4.4578030000	0.0348860000	0.3033920000
H	3.0706240000	0.1190230000	2.3338080000
H	0.6161200000	0.0725380000	2.2301600000
H	1.4543670000	-1.8377090000	-0.7968530000
H	0.8470720000	-0.7276480000	-2.0317420000
H	-0.9598170000	2.8801390000	-0.3376700000
H	-0.7922880000	1.9500140000	1.1631360000
H	-2.5275920000	1.2657590000	-1.2722270000
H	-3.0487500000	1.5208340000	0.3874350000
H	-0.7116800000	-1.9514730000	0.3802890000
H	-3.0925550000	-2.2478540000	0.3465640000
H	-3.9330500000	-1.1091670000	-0.7268870000
H	-3.9389440000	-0.8325830000	1.0110790000

thermodynamic data

Zero-point correction= 0.234920 (Hartree/Particle)
Thermal correction to Energy= 0.245956
Thermal correction to Enthalpy= 0.246900
Thermal correction to Gibbs Free Energy= 0.198035
Sum of electronic and zero-point Energies= -503.124201
Sum of electronic and thermal Energies= -503.113166
Sum of electronic and thermal Enthalpies= -503.112222
Sum of electronic and thermal Free Energies= -503.161086

	E (Thermal) KCal/Mol	CV Cal/Mol-Kelvin	S Cal/Mol-Kelvin
Total	154.340	44.466	102.845

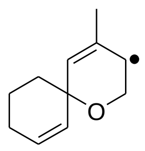

12-R2

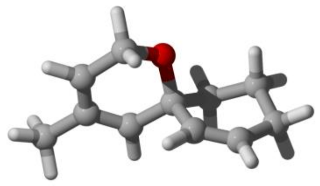

xyz-matrix

27
XYZ file generated by gabedit : coordinates in Angstrom

C	2.6278760000	0.0744290000	-1.0288880000
C	3.1911580000	-0.2212100000	0.3678040000
C	2.1517180000	-0.0230150000	1.4405640000
C	0.8399920000	0.0878060000	1.1978850000

C	0.2226280000	-0.0084330000	-0.1933720000
C	1.2461190000	-0.5659560000	-1.2004050000
O	-0.1190460000	1.3133060000	-0.6924190000
C	-1.2020670000	1.9542190000	-0.0249510000
C	-2.4016930000	1.0684730000	0.1257060000
C	-2.2834440000	-0.3120450000	0.0248940000
C	-1.0162970000	-0.8691840000	-0.1425280000
C	-3.5094170000	-1.2004910000	0.0884900000
H	3.3140620000	-0.2912380000	-1.8016760000
H	2.5370340000	1.1590040000	-1.1649680000
H	4.0618740000	0.4148050000	0.5767260000
H	3.5663390000	-1.2564010000	0.4146470000
H	2.5068480000	0.0280300000	2.4698800000
H	0.1475720000	0.2371580000	2.0244040000
H	1.3239480000	-1.6514050000	-1.0539300000
H	0.8544510000	-0.4011300000	-2.2102300000
H	-1.4437320000	2.8326060000	-0.6366740000
H	-0.8791590000	2.3424140000	0.9590790000
H	-3.3601510000	1.5371950000	0.3336680000
H	-0.8841230000	-1.9478250000	-0.1729290000
H	-3.2885040000	-2.1498720000	0.5879480000
H	-3.8770960000	-1.4391190000	-0.9176790000
H	-4.3268900000	-0.7121340000	0.6289580000

thermodynamic data

Zero-point correction= 0.234314 (Hartree/Particle)
Thermal correction to Energy= 0.245715
Thermal correction to Enthalpy= 0.246659
Thermal correction to Gibbs Free Energy= 0.195650
Sum of electronic and zero-point Energies= -503.119226
Sum of electronic and thermal Energies= -503.107825
Sum of electronic and thermal Enthalpies= -503.106881
Sum of electronic and thermal Free Energies= -503.157890

	E (Thermal)	CV	S
	KCal/Mol	Cal/Mol-Kelvin	Cal/Mol-Kelvin
Total	154.188	44.735	107.358

2. Results obtained with composite method CBS-QB3 for model system 11A and 11B

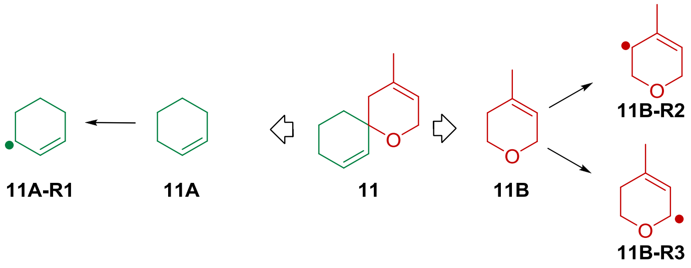

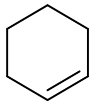	**11A**	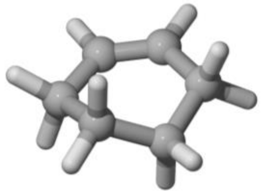

xyz-matrix

16
XYZ file generated by gabedit : coordinates in Angstrom

H	0.7296280000	-0.2739240000	-2.1308050000
H	3.2188580000	-0.1560800000	-1.9881000000
C	1.2316240000	-0.5035640000	-1.1882060000
H	2.4988170000	1.2285510000	-1.1694900000
C	2.6185590000	0.1423940000	-1.1234880000
H	1.3265160000	-1.5934330000	-1.1194090000
H	-0.0192030000	1.3490980000	-0.3991640000
C	0.3267990000	-0.0097600000	-0.0430230000
H	-0.9104980000	-0.8757630000	0.0574460000
H	3.7209330000	-1.2668120000	0.1129800000
C	3.3305300000	-0.2405080000	0.1790360000
H	4.2078770000	0.3962730000	0.3397070000
C	1.0876130000	-0.0254300000	1.2776560000
C	2.4130560000	-0.1387300000	1.3680890000
H	0.4922910000	0.0565970000	2.1822610000
H	2.8763990000	-0.1580660000	2.3519940000

thermodynamic data

Zero-point correction= 0.145723 (Hartree/Particle)
Thermal correction to Energy= 0.151240
Thermal correction to Enthalpy= 0.152185
Thermal correction to Gibbs Free Energy= 0.117076

Sum of electronic and zero-point Energies= -234.565778
Sum of electronic and thermal Energies= -234.560261
Sum of electronic and thermal Enthalpies= -234.559317
Sum of electronic and thermal Free Energies= -234.594426

	E (Thermal)	CV	S
	KCal/Mol	Cal/Mol-Kelvin	Cal/Mol-Kelvin
Total	94.905	21.515	73.893

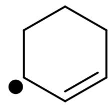	**11A-R1**	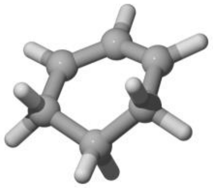

xyz-matrix

15
XYZ file generated by gabedit : coordinates in Angstrom

H	0.8364490000	-0.6892730000	-2.0391290000
H	1.4613520000	-1.8254240000	-0.8357820000
H	3.3773810000	-0.6456790000	-1.8386860000
C	1.3467880000	-0.7633680000	-1.0762580000
H	-0.8380330000	-0.8983840000	0.1457970000
C	2.7327700000	-0.1043350000	-1.1382380000
H	2.6242410000	0.9121310000	-1.5418890000
C	0.4485420000	-0.1183620000	-0.0033240000
H	0.1881070000	1.2191090000	-0.5011630000
C	3.3659780000	-0.0343810000	0.2177460000
H	4.4454780000	0.0347700000	0.2963250000
C	1.1959340000	-0.0498800000	1.3151630000
C	2.5833430000	-0.0125600000	1.3590980000
H	0.6199290000	-0.0026800000	2.2320770000
H	3.0709800000	0.0501170000	2.3282590000

thermodynamic data

Zero-point correction= 0.131905 (Hartree/Particle)
Thermal correction to Energy= 0.137418
Thermal correction to Enthalpy= 0.138362
Thermal correction to Gibbs Free Energy= 0.102714
Sum of electronic and zero-point Energies= -233.938449
Sum of electronic and thermal Energies= -233.932936
Sum of electronic and thermal Enthalpies= -233.931991

Sum of electronic and thermal Free Energies= -233.967640

	E (Thermal)	CV	S
	KCal/Mol	Cal/Mol-Kelvin	Cal/Mol-Kelvin
Total	86.231	21.571	75.028

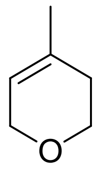	**11B**	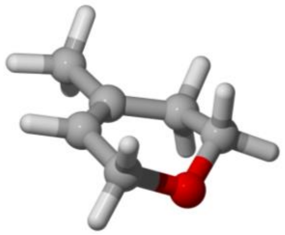

xyz-matrix

17
XYZ file generated by gabedit : coordinates in Angstrom

H	1.4126420000	2.4350590000	-1.5839850000
O	0.0241020000	1.0293970000	-1.2147570000
C	1.0969000000	1.8270020000	-0.7297110000
H	0.7376040000	2.5336760000	0.0382070000
H	-1.3910290000	-0.8396980000	-1.2083440000
C	2.2578840000	1.0419110000	-0.1948450000
H	3.1513210000	1.6088770000	0.0559650000
H	1.0993680000	-1.5365320000	-1.3357380000
C	-0.2927480000	-0.1468540000	-0.4311380000
C	0.9598490000	-1.0447220000	-0.3644460000
C	2.2165460000	-0.2781980000	-0.0181440000
H	-0.7956500000	0.2396950000	0.9760990000
H	3.6672200000	-1.8579260000	-0.2222020000
H	4.2565430000	-0.4477020000	0.6737710000
C	3.3827750000	-1.0776640000	0.4938730000
H	0.7962520000	-1.8490400000	0.3619140000
H	3.1288340000	-1.5891180000	1.4297260000

thermodynamic data

Zero-point correction= 0.149288 (Hartree/Particle)
Thermal correction to Energy= 0.156101
Thermal correction to Enthalpy= 0.157045
Thermal correction to Gibbs Free Energy= 0.118823
Sum of electronic and zero-point Energies= -309.790840
Sum of electronic and thermal Energies= -309.784026
Sum of electronic and thermal Enthalpies= -309.783082

Sum of electronic and thermal Free Energies= -309.821304

	E (Thermal)	CV	S
	KCal/Mol	Cal/Mol-Kelvin	Cal/Mol-Kelvin
Total	97.955	25.812	80.445

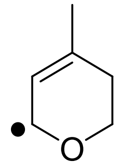	**11B-R3**	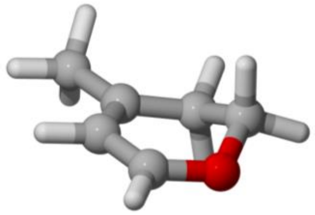

xyz-matrix

16
XYZ file generated by gabedit : coordinates in Angstrom

H	-2.1608480000	-1.8103500000	-0.0385580000
H	-2.0763180000	2.2389020000	-0.1342440000
O	-2.1995150000	0.2062140000	-0.1854030000
C	-1.4670710000	1.3470170000	-0.0851730000
H	-0.1480760000	-1.3271290000	-1.3606530000
C	-1.5259930000	-0.9804060000	0.2715880000
C	-0.1077240000	-1.0987460000	-0.2849810000
C	-0.0874800000	1.3448920000	0.0338120000
H	-1.4925020000	-0.9492100000	1.3674760000
H	0.4057650000	2.3039800000	0.1543950000
C	0.6578720000	0.1765490000	-0.0461220000
H	0.3860240000	-1.9539190000	0.1929690000
H	2.5859210000	-0.2692560000	-0.9057450000
C	2.1513510000	0.1395960000	0.0172700000
H	2.5724860000	1.1361140000	0.1696120000
H	2.5061100000	-0.5042530000	0.8337600000

thermodynamic data

Thermo
Zero-point correction= 0.135962 (Hartree/Particle)
Thermal correction to Energy= 0.142843
Thermal correction to Enthalpy= 0.143787
Thermal correction to Gibbs Free Energy= 0.104739
Sum of electronic and zero-point Energies= -309.171471
Sum of electronic and thermal Energies= -309.164590
Sum of electronic and thermal Enthalpies= -309.163646

Sum of electronic and thermal Free Energies= -309.202694

	E (Thermal)	CV	S
	KCal/Mol	Cal/Mol-Kelvin	Cal/Mol-Kelvin
Total	89.635	25.802	82.183

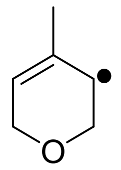	**11B-R2**	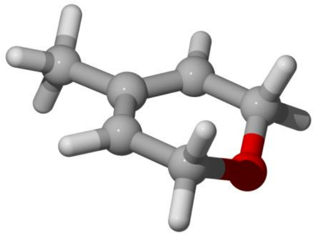

xyz-matrix

16
XYZ file generated by gabedit : coordinates in Angstrom

H	-1.8650990000	-2.0098550000	-0.5280490000
C	-1.4484150000	-1.1851800000	0.0563370000
O	-2.0110690000	-0.0000080000	-0.4908950000
H	-1.7957960000	-1.3224870000	1.0986380000
H	0.5637800000	-2.1487270000	0.0309510000
C	0.0502440000	-1.1935550000	0.0027040000
C	-1.4484100000	1.1851830000	0.0563160000
C	0.7578530000	0.0000020000	-0.0502740000
H	-1.8650640000	2.0098390000	-0.5281230000
C	0.0502420000	1.1935410000	0.0027270000
H	2.5756780000	-0.0001630000	-1.2184010000
H	-1.7958380000	1.3225450000	1.0985920000
H	2.7009790000	-0.8842010000	0.3035900000
C	2.2661830000	-0.0000010000	-0.1684230000
H	0.5637730000	2.1487160000	0.0309970000
H	2.7009590000	0.8843560000	0.3033130000

thermodynamic data

Zero-point correction= 0.134877 (Hartree/Particle)
Thermal correction to Energy= 0.142066
Thermal correction to Enthalpy= 0.143010
Thermal correction to Gibbs Free Energy= 0.102655
Sum of electronic and zero-point Energies= -309.158331

Sum of electronic and thermal Energies= -309.151142
Sum of electronic and thermal Enthalpies= -309.150197
Sum of electronic and thermal Free Energies= -309.190553

	E (Thermal)	CV	S
	KCal/Mol	Cal/Mol-Kelvin	Cal/Mol-Kelvin
Total	89.148	26.198	84.936

	**H (hydrogen atom)**	

thermodynamic data

Zero-point correction= 0.000000 (Hartree/Particle)
Thermal correction to Energy= 0.001416
Thermal correction to Enthalpy= 0.002360
Thermal correction to Gibbs Free Energy= -0.010654
Sum of electronic and zero-point Energies= -0.502156
Sum of electronic and thermal Energies= -0.500740
Sum of electronic and thermal Enthalpies= -0.499795
Sum of electronic and thermal Free Energies= -0.512810

	E (Thermal)	CV	S
	KCal/Mol	Cal/Mol-Kelvin	Cal/Mol-Kelvin
Total	0.889	2.981	27.392

Population Analysis: Spin densities and α-HOMOs of model system at CBS-QB3 level of theory.

**11A-R1**	**11B-R3**	**11B-R2**
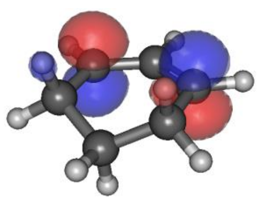	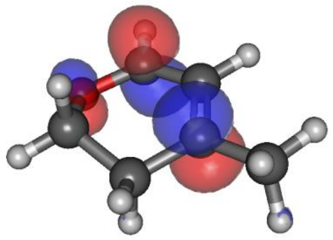	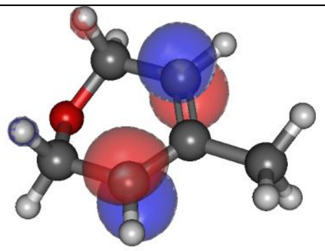
**α-HOMO**	**α-HOMO**	**α-HOMO**
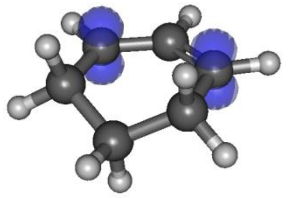	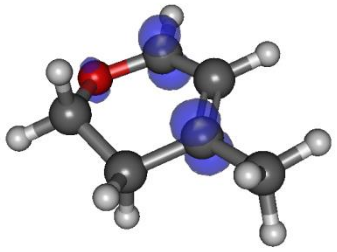	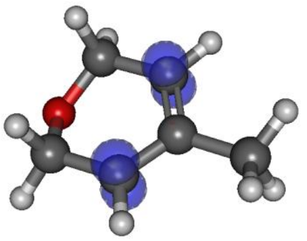
**spin density**	**spin density**	**spin density**

General Methods: The ^{1}H and ^{13}C NMR spectroscopic data were recorded with a 400 or 500 MHz spectrometer at ambient temperature. The samples were dissolved in $CDCl_3$. The coupling constant (*J*) is given in Hz. The chemical shifts δ are reported in ppm on a scale downfield from TMS, which was used as an internal standard, and the signal patterns are indicated as s (singlet), d (doublet), t (triplet), q (quartet), sext (sextet), m (multiplet), and br. (broad). Appropriate 1D and 2D NMR methods (e.g., COSY, HSQC and HMBC) were used for peak assignment. For HRMS *m*/*z* ratios are recorded in atomic mass units. Mass analysis was carried out using APCI or ESI mode. All of the reagents were reagent grade and used without further purification unless otherwise specified. Solvents for the reactions were distilled prior to use. All air or moisture-sensitive reactions were conducted under nitrogen or argon in flame or oven-dried glassware and were magnetically stirred. THF, toluene and diethyl ether were distilled from Na. Column chromatography was carried out with silica gel (40–63 mm) packed in glass columns. Technical grade ethyl acetate and petroleum ether, which were used for column chromatography, were distilled prior to use. R_f-values were detected by phosphomolybdic acid.

The filamentous fungus *Pleurotus sapidus* (PSA) was obtained from the German Collection of Microorganisms and Cell Cultures (DSMZ 8266), Braunschweig, Germany.
Production of biomass and lyophilisate were described previously by Fraatz et al. [20]. For all new oxidations with PSA, a parallel positive control experiment with the known substrate valencene and the same batch of PSA-lyophilisate was performed, confirming enzymatic activity of the lyophilisate used.

Epoxide 4a [8,21]**:** The diastereomeric mixture of *cis*- and *trans*-theaspirane **1** was separated by column chromatography (pentane/Et_2O, 95:5) in portions of 500 mg. A solution of theaspirane *trans*-**1** (3.89 g, 20.0 mmol) in 25 mL CH_2Cl_2 was added to a solution of *m*-CPBA

(5.80 g, 24.8 mmol) in 40 mL CH_2Cl_2 at 4 °C under continuous stirring. The solution was stirred for 60 min at 15 °C and subsequently quenched with 2 M aqueous NaOH (100 mL). The reaction mixture was washed with water (40 mL), dried with anhydrous $MgSO_4$, filtered and concentrated in vacuo. The residue was purified by column chromatography (pentane/Et_2O, 85:15) to give epoxide **4a** (3.58 g, 17.0 mmol, 85%) and epoxide **4b** (0.31 g, 1.46 mmol, 7%) as colorless crystals; R_f 0.49 (pentane/Et_2O, 8:2).

4a: ^{1}H NMR (400 MHz, $CDCl_3$) δ 4.07–3.99 (m, 1H, 2-H), 3.03 (br. s, 1H, 7-H), 2.13–2.05 (m, 1H, 4-H), 1.93–1.80 (m, 4H, 4-H, 3-H, 8-H,8-H), 1.34–1.25 (m, 2H, 9-H, 3-H), 0.98–0.92 (m, 1H, 9-H), 1.29 (s, 3H, 11-H), 1.22 (d, $^3J_{H,H}$ = 6.1 Hz, 3H, 14-H), 0.84 (s, 3H, 12-H), 0.76 (s, 3H, 13-H) ppm. **^{13}C NMR** (100 MHz, $CDCl_3$) δ 86.8 (C5), 77.2 (C2), 64.7 (C6), 62.8 (C7), 35.8 (C10), 34.4 (C3), 31.7 (C4), 30.0 (C9), 24.3 (C13), 22.1 (C8), 21.7 (C12), 20.8 (C14), 19.9 (C11) ppm. **GC–MS** *m*/*z* 210 (34, M^+), 154 (73, M-56), 153 (22, M-57), 139 (32, M-71), 126 (100, M-84), 125 (82, M-85), 111 (56, M-99), 85 (39, M-125), 69 (66, M-141), 55 (73, M-155), 43 (81, M-167).

4b: ^{1}H NMR (400 MHz, $CDCl_3$) δ 4.35–4.20 (m, 1H, 2-H), 2.95 (br. s, 1H, 7-H), 2.15–2.06 (m, 1H, 3-H), 2.01–1.95 (m, 1H, 8-H), 1.94–1.89 (m, 2H, 4-H), 1.84–1.75 (m, 1H, 8-H), 1.60–1.53 (m, 1H, 9-H), 1.52–1.46 (m, 1H, 3-H), 1.36 (s, 3H, 11-H), 1.23 (d, $^3J_{H,H}$ = 6.0 Hz, 3H, 14-H), 1.00–0.94 (m, 1H, 9-H), 0.94 (s, 3H, 12-H), 0.92 (s, 3H, 13-H) ppm. **^{13}C NMR** (100 MHz, $CDCl_3$) δ 86.9 (C5), 75.3 (C2), 61.8 (C7), 60.5 (C6), 35.6 (C10), 34.0 (C3), 30.2 (C9), 30.1 (C4), 23.8/23.6/23.6 (C11/C12/C13), 21.6/21.6 (C8/C14) ppm. **GC–MS** *m*/*z* 210 (34, M^+), 154 (73, M-56), 153 (22, M-57), 139 (32, M-71), 126 (95, M-84), 125 (41, M-85), 112 (68, M-99), 83 (39, M-125), 69 (66, M-141), 55 (73, M-155), 43 (100, M-167).

Allyl alcohol 22 [8,21]**:** To a magnetically stirred solution of tetramethylpiperidine (3.34 mL, 19.7 mmol) in 50 mL dry toluene was added *n*-BuLi (16.0 mL 1.3 M solution in hexanes, 20.8 mmol) dropwise at 0 °C whereby the solution turned yellow-orange. Stirring was

continued for 30 min. at 0 °C and subsequently diethylaluminium chloride (1 M solution, 20.0 mL, 20.0 mmol) was added. After an additional stirring for 40 min at 0 °C epoxide **4a** (1.03 g, 4.91 mmol) was added and the solution was stirred for 30 min at 0 °C and then 2 h at rt. The reaction mixture was quenchend with saturated aqueous $NaHCO_3$ (10 mL) and extracted three times with Et_2O (30 mL). The combined organic phases were washed with saturated aqueous $KHSO_4$ and with brine, dried over $MgSO_4$, filtered and concentrated in vacuo. The residue was coevaporated three times with MeOH to give allyl alcohol **22** (0.96 g, 4.57 mmol, 93%) as colorless oil; R_f = 0.12 (pentane/Et_2O, 9:1). **^{1}H NMR** (400 MHz, $CDCl_3$) δ 5.17 (s, 1H, 11-H), 4.94 (s, 1H, 11-H), 4.50–4.47 (m, 1H, 7-H), 3.95–3.87 (m, 1H, 2-H), 2.09–1.83/1.50–1.33 (2 m, 8H, 3-H/4-H/8-H/9-H), 1.17 (d, $^3J_{H,H}$ = 6.1 Hz, 3H, 14-H), 0.85/0.77 (2 s, 6H, 12-H/13-H) ppm. **^{13}C NMR** (100 MHz, $CDCl_3$) δ 152.3 (C6), 106.7 (C11), 90.2 (C5), 73.5 (C2), 71.0 (C7), 37.8 (C10), 34.7 (C9), 33.3/28.6 (C3/C4), 32.3 (C8), 23.7/23.6 (C12/C13), 21.3 (C14) ppm. **GC–MS** *m*/*z* 192 (51, M-8), 177 (40, M-33), 163 (24, M-47), 153 (63, M-57), 149 (22, M-61), 141 (40, M-69), 136 (29, M-74), 121 (46, M-89), 111 (35, M-99), 93 (76, M-117), 85 (98, M-125), 77 (44, M-133), 55 (100, M-155).

Vitispirane 23 [8,21]**:** To a solution of pyridine (50 mL, 620 mmol) and acetanhydride (25 mL, 264 mmol) was added the allylic alcohol **22** (1.05 g, 4.99 mmol). The resulting solution was stirred at rt for 24 h. The reaction was monitored by TLC. The crude product was concentrated in vacuo and dissolved in dioxane (12 mL). To the resulting solution was added $Pd(OAc)_2$ (15.7 mg, 0.07 mmol), PPh_3 (184 mg, 0.70 mmol) and $CaCO_3$ (700 mg, 6.99 mmol) and the resulting heterogeneous mixture was stirred under reflux under nitrogen for 6.5 h. Then the mixture was cooled to rt, diluted with Et_2O (5 mL) and filtrated. The filtrate was washed successively with saturated aqueous $NaHCO_3$ (30 mL) and brine, dried over $MgSO_4$, filtered and concentrated in vacuo. The residue was purified by column chromatography (pentane/CH_2Cl_2, 4:1) to give vitispirane (**23**) (0.64 g, 3.32 mmol, 84%) as colorless oil; R_f = 0.25 (pentane/CH_2Cl_2, 9:1). **^{1}H NMR** (400 MHz, $CDCl_3$) δ 6.07–6.04 (m, 1H, 7-H), 5.61–

5.57 (m, 1H, 8-H), 5.06 (s, 1H, 11-H), 4.85 (s, 1H, 11-H), 4.32–4.28 (m, 1H, 2-H), 2.16–1.94/1.62–1.36 (2m, 6H, 3-H/4-H/9-H), 1.22 (d, $^{3}J_{H,H}$ = 6.1 Hz, 3H, 14-H), 0.95/0.88 (2s, 6H, 12-H/13-H) ppm. **^{13}C NMR** (100 MHz, $CDCl_3$) δ 149.8 (C6), 128.7 (C7), 127.3 (C8), 109.2 (C11), 89.1 (C5), 76.0 (C2), 41.3 (C9), 36.3 (C4), 32.9 (C3), 23.7/23.4 (C12/C13), 21.7 (C14). **GC–MS** *m*/*z* 192 (86, M^+), 177 (60, M-CH_3), 163 (12, M-29), 149 (29, M-43), 136 (44, M-56), 135 (31, M-57), 126 (17, M-66), 121 (60, M-71), 119 (34, M-73), 107 (33, M-85), 93 (100, M-99), 91 (56, M-102), 77 (41, M-115), 65 (28, M-127), 55 (37, M-137) ppm.

Spiroethers 7 and 8 [22]: To a flame-dried round-bottom flask was added dry CH_2Cl_2 (40 mL) and cooled to −20 °C. Cyclohexanone (**5**) (2.20 mL, 20.0 mmol) and TMS-ether **6** (3.79 g, 23.9 mmol) were added and stirred for 5 min at −20 °C. The solution was treated with TMSOTf (725 µL, 4.00 mmol) and stirred at −20 °C for 4 h under N_2. The reaction was monitored by TLC. The reaction mixture was diluted with saturated aqueous $NaHCO_3$ solution (10 mL) and the aqueous layer was extracted with CH_2Cl_2 (2 × 10 mL). The combined organic layers were dried over $MgSO_4$, filtered and concentrated in vacuo. The resulting bright yellow oil was purified by column chromatography (pentane/CH_2Cl_2, 4:1→1:1) to give spiroether **7** (1.50 g, 9.02 mmol, 45%) and **8** (1.47 g, 8.84 mmol, 44%) as colorless oils; spiroether **7**: R_f = 0.34 (pentane/CH_2Cl_2, 2:1). **^{1}H NMR** (400 MHz, $CDCl_3$) δ 5.35 (m, 1H, 3-H), 4.06–4.04 (m, 2H, 2-H), 1.83 (br. s, 2H, 5-H), 1.66 (br. s, 3H, 12-H), 1.72–1.24 (m, 10H, 7-11-H) ppm. **^{13}C NMR** (100 MHz, $CDCl_3$) δ 130.3 (C4), 118.7 (C3), 70.9 (C6), 60.4 (C2), 40.5 (C5), 35.0 (C7/C11), 26.3 (C9), 23.5 (C12), 22.1 (C8/C10) ppm. **GC–MS** *m*/*z* 166 (17, M^+), 148 (10, M-18), 123 (14, M-43), 110 (14, M-56), 99 (21, M-67), 95 (28, M-71), 81 (25, M-85), 68 (100, M-98), 55 (58, M-111).

Spiroether 8: R_f = 0.4 (pentane/CH_2Cl_2, 2:1). **^{1}H NMR** (400 MHz, $CDCl_3$) δ 5.34–5.32 (m, 1H, 5-H), 3.76 (t, $^{3}J_{H,H}$ = 5.6 Hz, 2H, 2-H), 1.96–1.93 (m, 2H, 3-H), 1.68 (m, 3H, 12-H), 1.66–1.21 (m, 10H, 7-11-H) ppm. **^{13}C NMR** (100 MHz, $CDCl_3$) δ 130.9 (C4), 128.7 (C5),

72.4 (C6), 58.8 (C2), 35.9 (C7/C11), 30.3 (C3), 25.8 (C9), 23.5 (C12), 21.9 (C8/C10) ppm. **GC–MS** *m*/*z* 166 (12, M^+), 151 (10, M-15), 137 (5, M-29), 123 (100, M-43), 110 (10, M-56), 79 (9, M-47), 67 (8, M-99), 55 (9, M-111).

Spiroethers 11 and 12: To a flame-dried round-bottom flask was added dry CH_2Cl_2 (20 mL) and cooled to −20 °C. Cyclohexenon **10** (0.96 g, 9.99 mmol) and TMS-ether **6** (1.58 g, 10 mmol) were added and stirred for 5 min at −20 °C. The solution was treated with TMSOTf (362 µL, 2.00 mmol) and stirred at −20 °C for 5 h under N_2. The reaction was monitored by TLC. The reaction mixture was diluted with saturated aqueous $NaHCO_3$ solution (10 mL) and the aqueous layer was extracted with CH_2Cl_2 (2 × 10 mL). The combined organic layers were dried over $MgSO_4$, filtered and concentrated in vacuo. The resulting bright yellow oil was purified by column chromatography (pentane/Et_2O, 100:1.5→100:4) to give spirother **11** (0.61 g, 3.70 mmol, 37%) and **12** (0.49 g, 3.00 mmol, 30%) as colorless oils; Spiroether **11**: R_f = 0.38 (pentane/Et_2O, 50:1). **^{1}H NMR** (400 MHz, $CDCl_3$) δ 5.86 (td, $^3J_{H,H}$ = 10.0 Hz, $^3J_{H,H}$ = 3.6 Hz, 1H, 8-H), 5.72 (d, $^3J_{H,H}$ = 10.0 Hz, 1H, 7-H), 5.43 (br.s, 1H, 3-H), 4.17–4.05 (m, 2H, 2-H), 2.11–1.88 (m, 4H, 5-H/9-H), 1.84–1.78 (m, 2H, 10-H/11-H), 1.69 (s, 3H, 12-H), 1.63–1.52 (m, 1H, 10-H/11-H) ppm. **^{13}C NMR** (100 MHz, $CDCl_3$) δ 131.1 (C8), 130.4 (C4), 130.2 (C7), 119.3 (C3), 69.2 (C6), 61.3 (C2), 40-4 (C5), 33.9 (C11), 25.8 (C9), 23.6 (C12), 18.9 (C10) ppm. **GC–MS** *m*/*z* 164 (11, M^+), 149 (12, M-CH_3), 136 (82, M-25), 121 (100, M-43), 105 (11, M-59), 91 (34, M-73), 79 (16, M-85), 77 (17, M-87), 67 (8, M-97), 55 (11, M-109).

Spiroether 12: R_f = 0.30 (pentane/Et_2O, 50:1). **^{1}H NMR** (400 MHz, $CDCl_3$) δ 5.82 (td, $^3J_{H,H}$ = 10.0 Hz, $^3J_{H,H}$ = 3.8 Hz, 1H, 8-H), 5.59 (d, $^3J_{H,H}$ = 10.0 Hz, 1H, 7-H), 5.30–5.29 (m, 1H, 5-H), 3.86–3.81 (m, 1H, 2-H), 3.78–3.72 (m, 1H, 2-H), 2.10–1.89 (m, 4H, 3-H/9-H), 1.85–1.72 (m, 2H, 10-H/11-H), 1.70 (s, 3H, 12-H), 1.65–1.58 (m, 1H, 10-H), 1.56–1.50 (m, 1H, 11-H) ppm. **^{13}C NMR** (100 MHz, $CDCl_3$) δ 131.6 (C4), 130.4 (C7), 129.9 (C8), 127.4 (C5), 70.9

(C6), 59.5 (C2), 34.1 (C11), 30.0 (C3), 25.3 (C9), 23.5 (C12), 18.9 (C10) ppm. **GC–MS** *m*/*z* 164 (11, M^+), 149 (12, M-CH_3), 136 (82, M-25), 121 (100, M-43), 105 (11, M-59), 91 (34, M-73), 79 (16, M-85), 77 (17, M-87), 67 (8, M-97), 55 (11, M-109). HRMS: calcd. for $C_{11}H_{16}O$ $[M+H]^+$ 165.1274; found 165.1269.

General Procedure for biocatalytic oxidations with PSA: 600 mg PSA lyophilisate were dissolved in 30 mL Tris HCl buffer. The dried biomass was rehydrated by stirring at 900 rpm for 10 min. The substrate was added and the solution was stirred at 900 rpm at rt. The reaction progress was controlled by GC-FID. After 24 h another 400 mg of PSA lyophilisate and 20 mL buffer were added. After 48 h the reaction was stopped by adding 50 mL of Et_2O and further stirring of the reaction mixture for 30 min. The solution was filtered and the aqueous phase was extracted with Et_2O three times. The combined organic phases were dried with $MgSO_4$, filtered and concentrated in vacuo. The crude product was analyzed with GC–MS and if necessary GCO and if required purified by column chromatography.

Physical and spectral data of the products:

Biotransforamtion of vitispirane 23; according to the general procedure vitispirane **23** (192 mg, 1.00 mmol) was treated with 1 g PSA-lyophilisate for 48 h. The crude product was purified by column chromatography (Et_2O, then EtOAc) to afford the enone **24** (18.0 mg, 8%) and the allylic alcohol **26** (36.2 mg, 16%). The diastereomeric mixture **26** was separated via HPLC (conditions: Waters Sunfire 5 µm 250*4.6 mm, flow: 1 mL/min, UV detection at 202 nm, gradient 10–90% MeCN in H_2O (0.05% TFA)); enone **24**: R_f = 0.25 (Et_2O). **^{1}H NMR** (400 MHz, $CDCl_3$) δ 5.94 (d, $^4J_{H,H}$ = 1.2 Hz, 1H, 7-H), 4.54 (dd, $^2J_{H,H}$ = 15.2 Hz, $^4J_{H,H}$ = 1.2 Hz, 1H, 11-H), 4.25–4.18 (m,, 1H, 2-H), 4.16 (dd, $^2J_{H,H}$ = 15.2 Hz, $^4J_{H,H}$ = 1.2 Hz, 1H, 11-H), 2.44–2.37 (m, 2H, 4-H/9-H), 2.25 (dd, $^2J_{H,H}$ = 17.4 Hz, $^4J_{H,H}$ = 1.2 Hz, 1H, 9-H), 2.10–2.03 (m, 1H, 3-H), 1.93–1.85 (m, 1H, 4-H), 1.60–1.49 (m, 1H, 3-H), 1.31 (d, $^3J_{H,H}$ = 5.8 Hz, 3H, 14-H), 1.04/0.98 (2s, 6H, 12-H/13-H) ppm. **^{13}C NMR** (100 MHz, $CDCl_3$) δ 198.7 (C8), 167.3 (C6), 123.1 (C7), 89.1 (C5), 78.3 (C2), 62.6 (C11), 50.3 (C9), 41.2 (C10), 34.3

(C3), 33.4 (C4), 24.3/23.1 (C12/C13), 20.7 (C14) ppm. **GC–MS** *m*/*z* 224 (1, M^+), 168 (100, M-56), 135 (15, M-89), 121 (21, M-103), 112 (33, M-112), 97 (27, M-127), 84 (21, M-140), 67 (21, M-157), 55 (53, M-169). HRMS (APCI): calcd. for $C_{13}H_{20}O_3$ $[M+H]^+$ 225.1485; found 225.1478.

Allylic alcohol 26a: R_f = 0.1 (EtOAc). **^{1}H NMR** (600 MHz, $CDCl_3$) δ 5.67 (s, 1H, 7-H), 4.34 (d, $^2J_{H,H}$ = 12.0 Hz, 1H, 11-H), 4.25–4.20 (m, 1H, 2-H), 4.19–4.15 (m, 1H, 8-H), 3.92 (d, $^2J_{H,H}$ = 12.0 Hz, 1H, 11-H), 2.16–2.11 (m, 1H, 4-H), 2.03–1.99 (m, 1H, 3-H), 1.92–1.86 (m, 1H, 9-H), 1.80–1.74 (m, 1H, 4-H), 1.72–1.69 (m, 1H, 9-H), 1.51–1.44 (m, 1H, 3-H), 1.30 (d, $^3J_{H,H}$ = 5.0 Hz, 3H, 14-H), 1.12 (s, 3H, 12-H), 0.89 (s, 3H, 13-H) ppm. **^{13}C NMR** (150 MHz, $CDCl_3$) δ 144.73 (C6), 125.60 (C7), 89.66 (C5), 77.28 (C2), 64.57 (C11), 64.10 (C8), 42.85 (C9), 35.63 (C10), 34.34 (C3), 33.20 (C4), 24.54 (C13), 23.98 (C12), 20.74 (C14) ppm. **GC–MS** *m*/*z* 208 (6, M-18), 170 (34, M-56), 161 (21, M-65), 152 (100, M-74), 126 (72, M-100), 119 (24, M-107), 111 (30, M-115), 105 (44, M-121), 96 (44, M-130), 91 (47, M-135), 77 (32, M-149), 55 (58, M-171). HRMS (ESI): calcd. for $C_{13}H_{22}O_3$ $[M+Na]^+$ 249.1461; found 249.1467.

Allylic alcohol 26b: R_f = 0.1 (EtOAc). **^{1}H NMR** (600 MHz, $CDCl_3$) δ 5.64 (s, 1H, 7-H), 4.37–4.31 (m, 2H, 11-H, 8-H), 4.20 (m, 1H, 2-H), 3.87 (d, $^2J_{H,H}$ = 12.0 Hz, 1H, 11-H), 2.22–2.17 (m, 1H, 4-H), 2.02–1.97 (m, 1H, 3-H), 1.92–1.89 (m, 1H, 4-H), 1.89–1.86 (m, 1H, 9-H), 1.53–1.49 (m, 1H, 3-H), 1.49–1.46 (m, 1H, 9-H), 1.30 (d, $^3J_{H,H}$ = 5.0 Hz, 3H, 14-H), 0.99 (s, 3H, 12-H), 0.92 (s, 3H, 13-H) ppm. **^{13}C NMR** (150 MHz, $CDCl_3$) δ 144.36 (C6), 127.62 (C7), 89.78 (C5), 77.42 (C2), 65.77 (C8), 64.76 (C11), 44.75 (C9), 39.59 (C10), 35.23 (C4), 34.27 (C3), 24.60 (C13), 21.89 (C12), 20.70 (C14) ppm. **GC–MS** *m*/*z* 208 (6, M-18), 170 (34, M-56), 161 (21, M-65), 152 (100, M-74), 126 (72, M-100), 119 (24, M-107), 111 (30, M-115), 105 (44, M-121), 96 (44, M-130), 91 (47, M-135), 77 (32, M-149), 55 (58, M-171). HRMS (ESI): calcd. for $C_{13}H_{22}O_3$ $[M+Na]^+$ 249.1461; found 249.1467

Biotransformation of spiroether 7; according to the general procedure, spiroether **7** (150 mg, 0.90 mmol) was treated with 600 mg PSA lyophilisate for 48 h. The crude product was purified by column chromatography (pentane/Et_2O, 4:1 → 1:1) to afford spirolactone **14** (72.9 mg, 45%) as a colorless oil; R_f = 0.49 (pentane/Et_2O, 4:1). **^{1}H NMR** (400 MHz, $CDCl_3$): δ 5.79–5.78 (m, 1H, 3-H), 2.30 (s, 2H, 5-H), 1.94 (s, 3H, 12-H), 1.96–1.30 (m, 10H, 7-11-H) ppm. **^{13}C NMR** (100 MHz, $CDCl_3$) δ 164.9 (C2), 154.9 (C4), 116.1 (C3), 80.6 (C6), 40.0 (C5), 36.4 (C7/C11), 25.5 (C9), 23.4 (C12), 21.7 (C8/C10) ppm. **GC–MS** *m*/*z* 180 (18, M^+), 152 (7, M-28), 137 (19, M-43), 124 (29, M-56), 109 (14, M-71), 96 (10, M-84), 82 (100, M-98), 55 (22, M-125). HRMS (APCI): calcd. for $C_{11}H_{16}O_2$ $[M+H]^+$ 181.1223; found 181.1218.

Biotransformation of spiroether 8; according to the general procedure spiroether **8** (125 mg, 0.75 mmol) was treated with 600 mg PSA lyophilisate for 48 h. No conversion was observed.

Biotransformation of spirodiene 11; according to the general procedure spiroether **11** (200 mg, 1.21 mmol) was treated with 800 mg PSA lyophilisate for 48 h. The crude product was purified by column chromatography (pentane/Et_2O, 3:2) to afford spirolactone **17** (69.0 mg, 0.39 mmol, 32%) as a colorless oil; R_f = 0.3 (pentane/Et_2O, 1:1). **^{1}H NMR** (400 MHz, $CDCl_3$) δ 5.93–5.89 (m, 1H, 8-H), 5.83–5.82 (m, 1H, 3-H), 5.78–5.76 (m, 1H, 7-H), 2.44 (d, $^4J_{H,H}$ =17.8 Hz, 1H, 5-H), 2.33 (d, $^2J_{H,H}$ =17.8 Hz, 1H, 5-H), 2.11–1.56 (m, 6H, 9-11-H), 1.95 (s, 3H, 12-H) ppm. **^{13}C NMR** (100 MHz, $CDCl_3$): δ = 164.7 (C2), 154.6 (C4), 132.9 (C8), 127.6 (C7), 116.5 (C3), 78.1 (C6), 40.1 (C5), 34.7 (C11), 25.1 (C9), 23.4 (C12), 18.3 (C10) ppm. **GC–MS** *m*/*z* 178 (2, M^+), 150 (9, M-28), 91 (6, M-87), 82 (100, M-96), 79 (12, M-99), 39 (18, M-139). HRMS (APCI): calcd. for $C_{11}H_{16}O_2$ $[M+H]^+$ 118.1223; found 118.1218.

Biotransformation of spirodiene 12; according to the general procedure spiroether **12** (100 mg, 0.60 mmol) was treated with 1 g PSA lyophilisate for 7 d. No conversion was observed.

NMR spectra

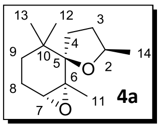

$C_{13}H_{22}O_2$

M = 210.31 g mol^{-1}

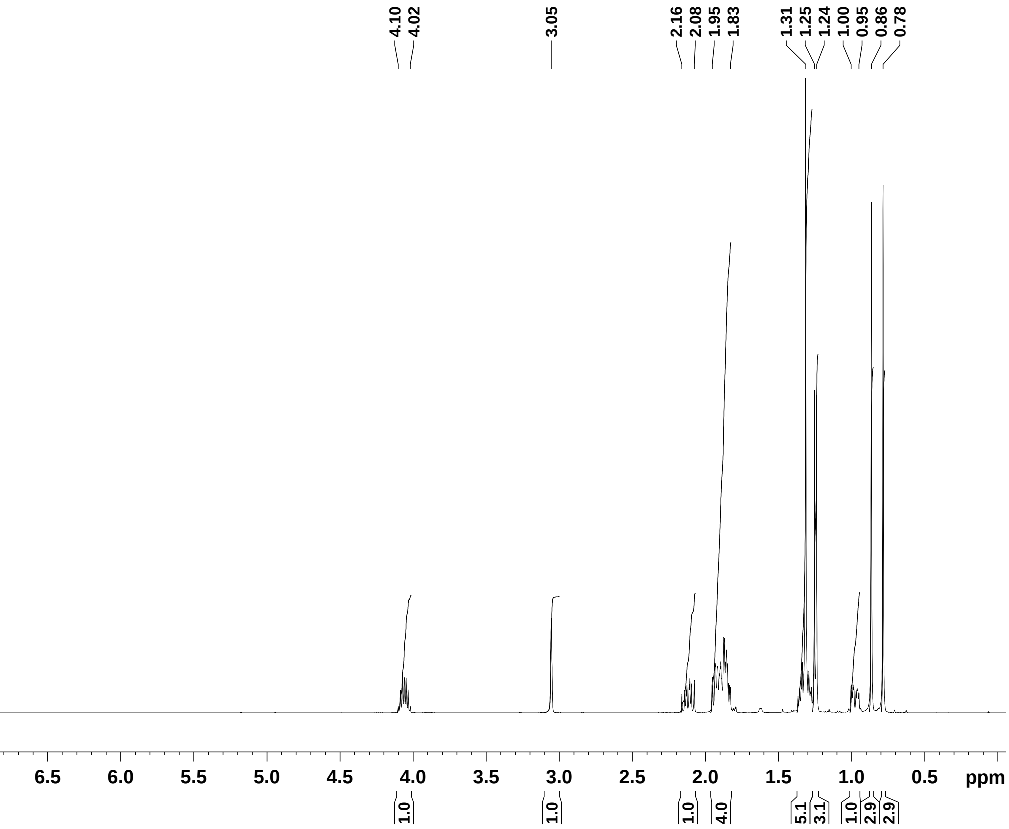

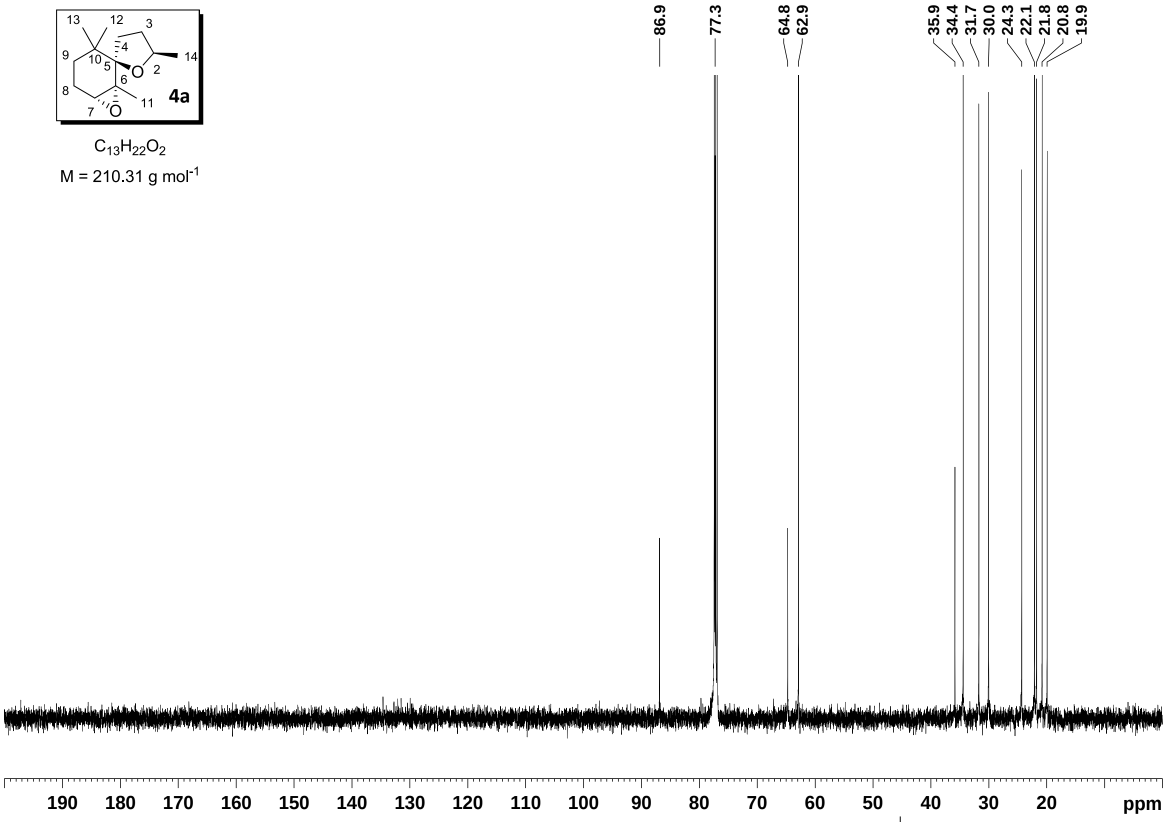

$C_{13}H_{22}O_2$
M = 210.31 g mol^{-1}

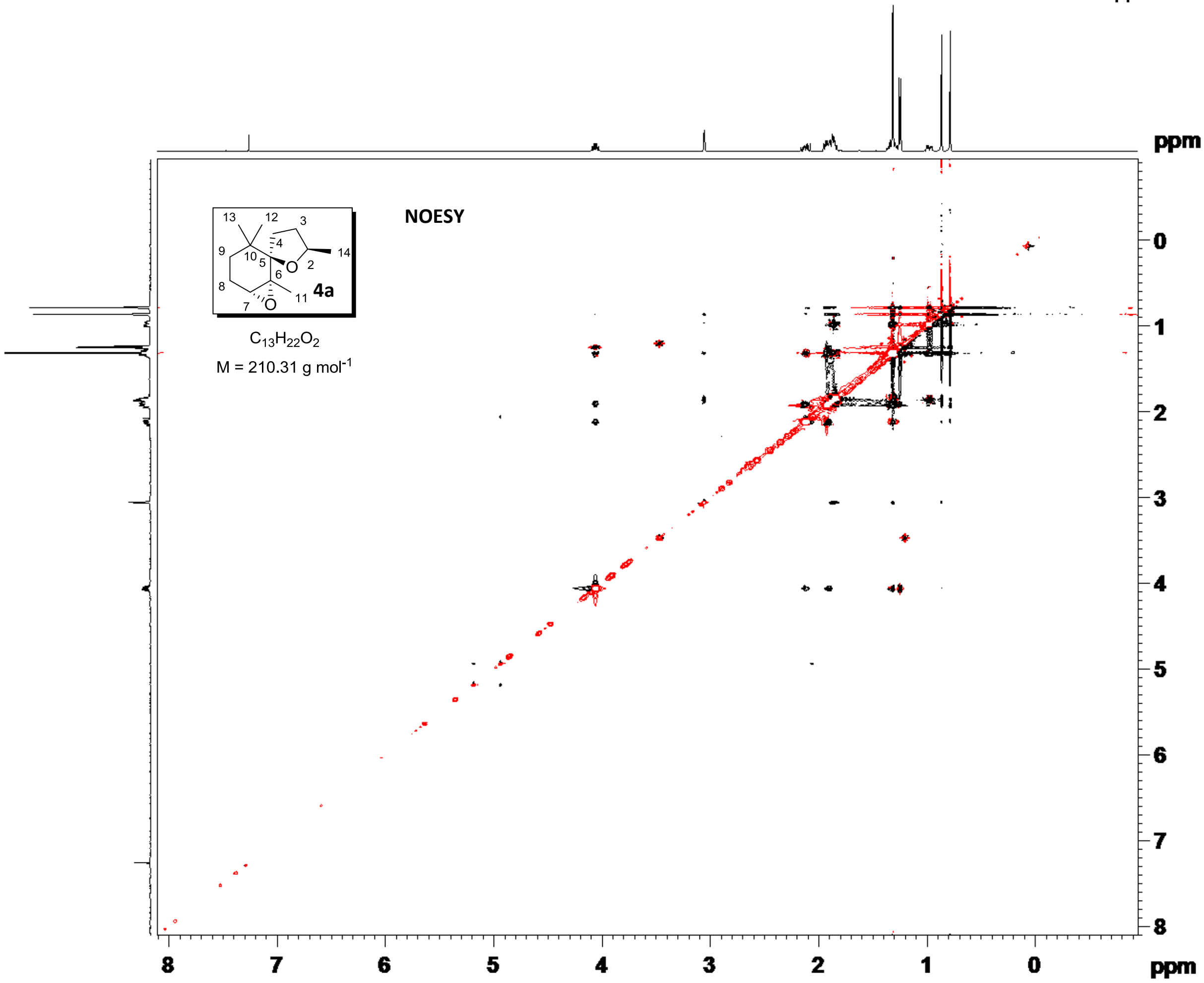

$C_{13}H_{22}O_2$
M = 210.31 g mol^{-1}

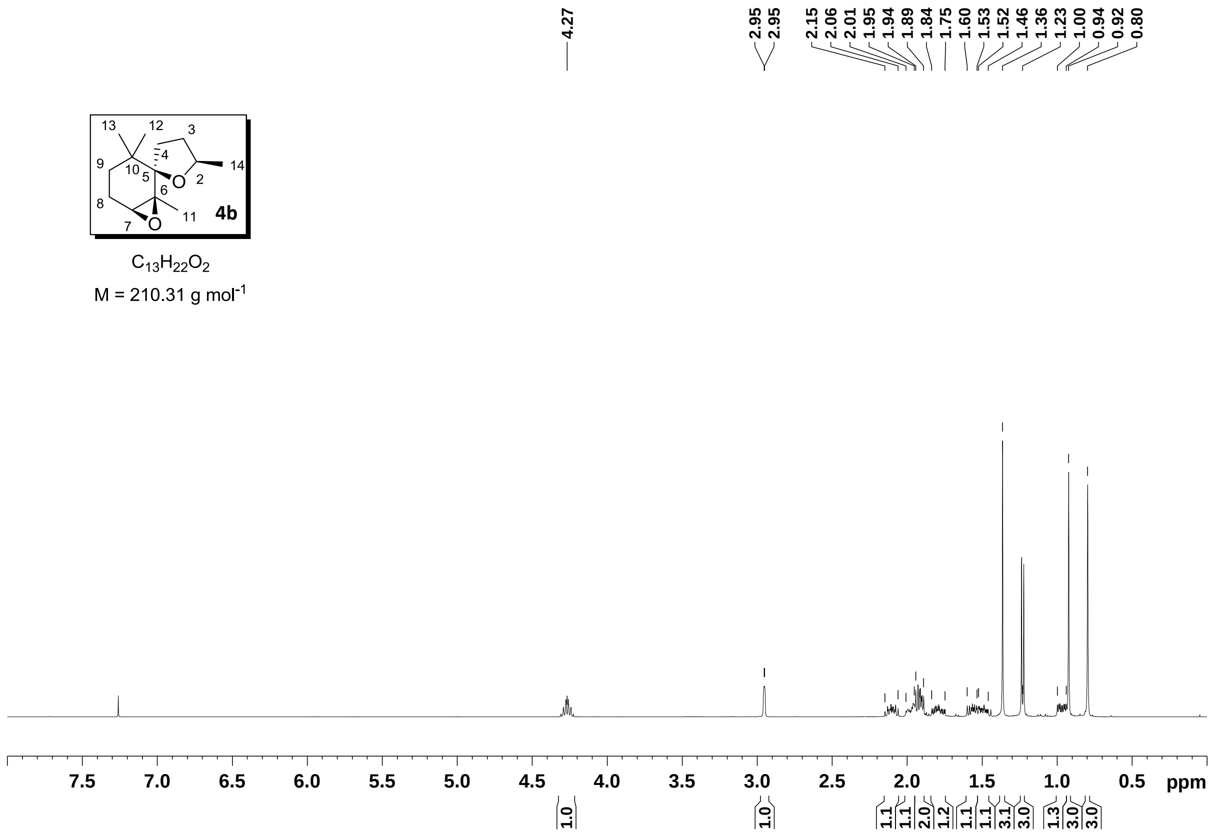

$C_{13}H_{22}O_2$

M = 210.31 g mol^{-1}

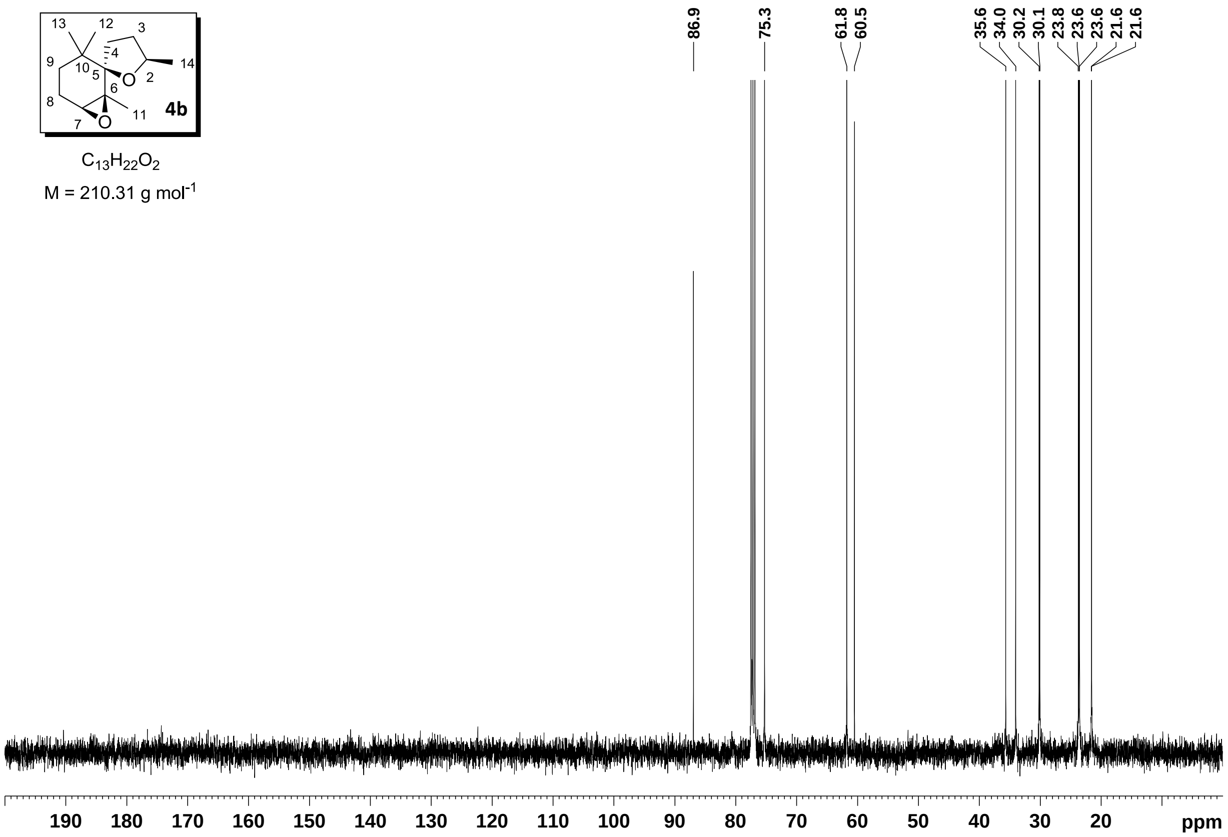

$C_{13}H_{22}O_2$

M = 210.31 g mol^{-1}

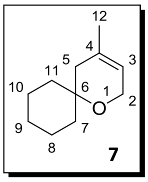

$C_{11}H_{18}O$

M = 166.26 g mol^{-1}

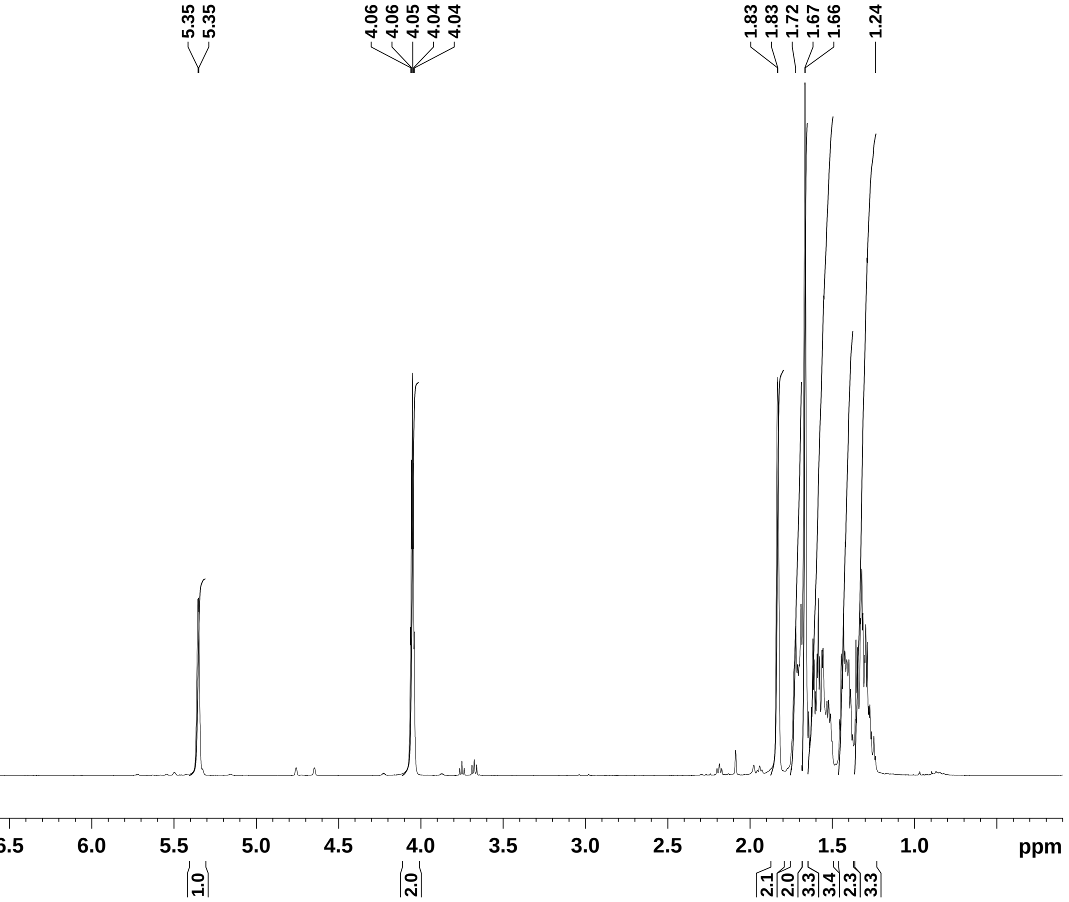

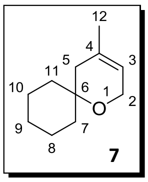

$C_{11}H_{18}O$

M = 166.26 g mol^{-1}

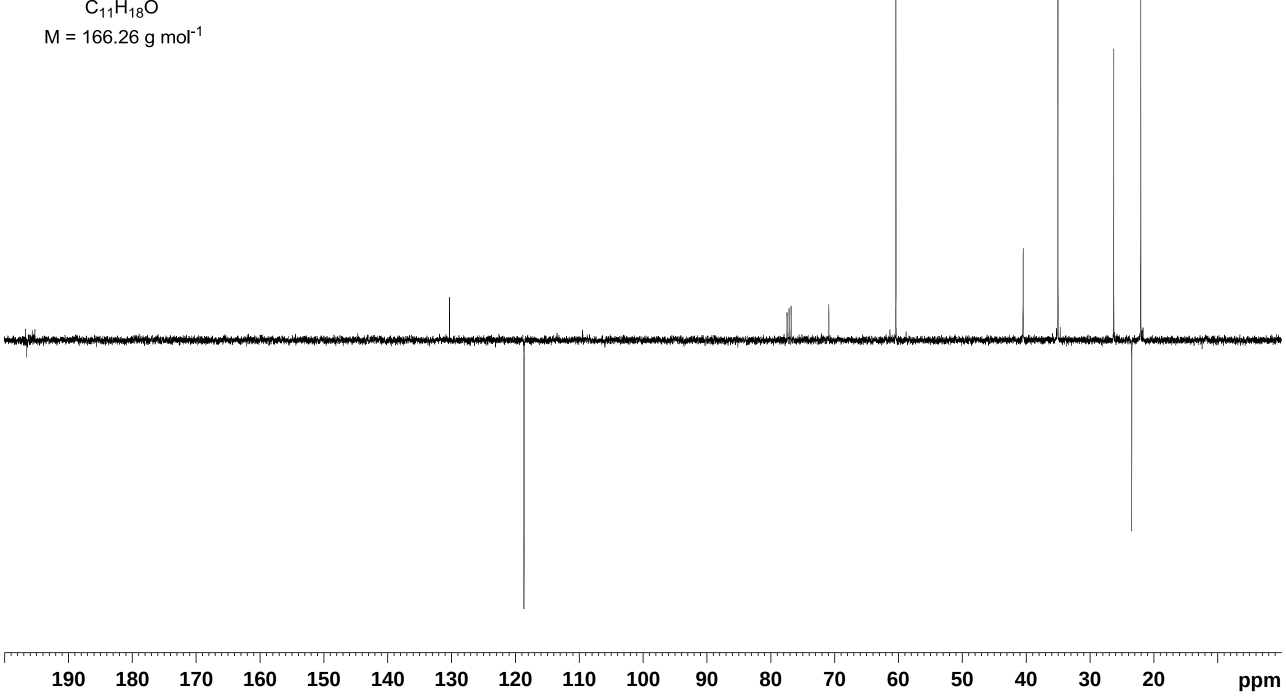

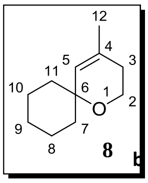

$C_{11}H_{18}O$
M = 166.26 g mol^{-1}

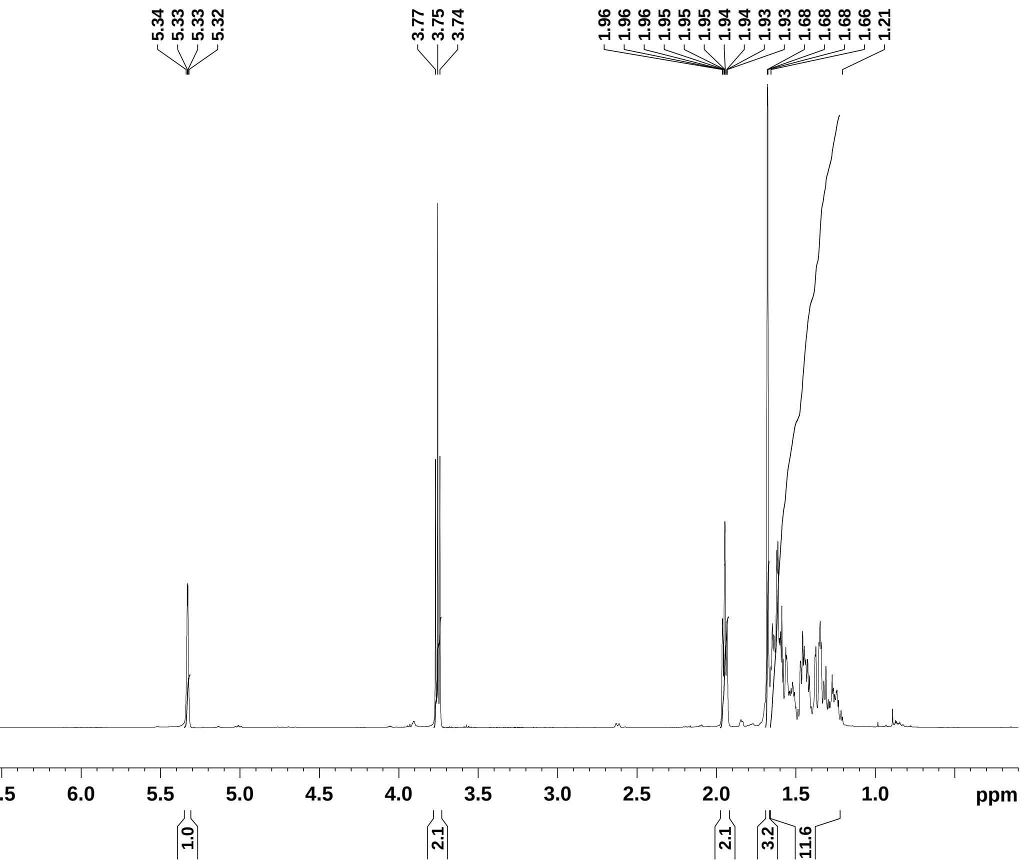

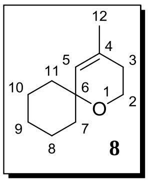

$C_{11}H_{18}O$
M = 166.26 g mol^{-1}

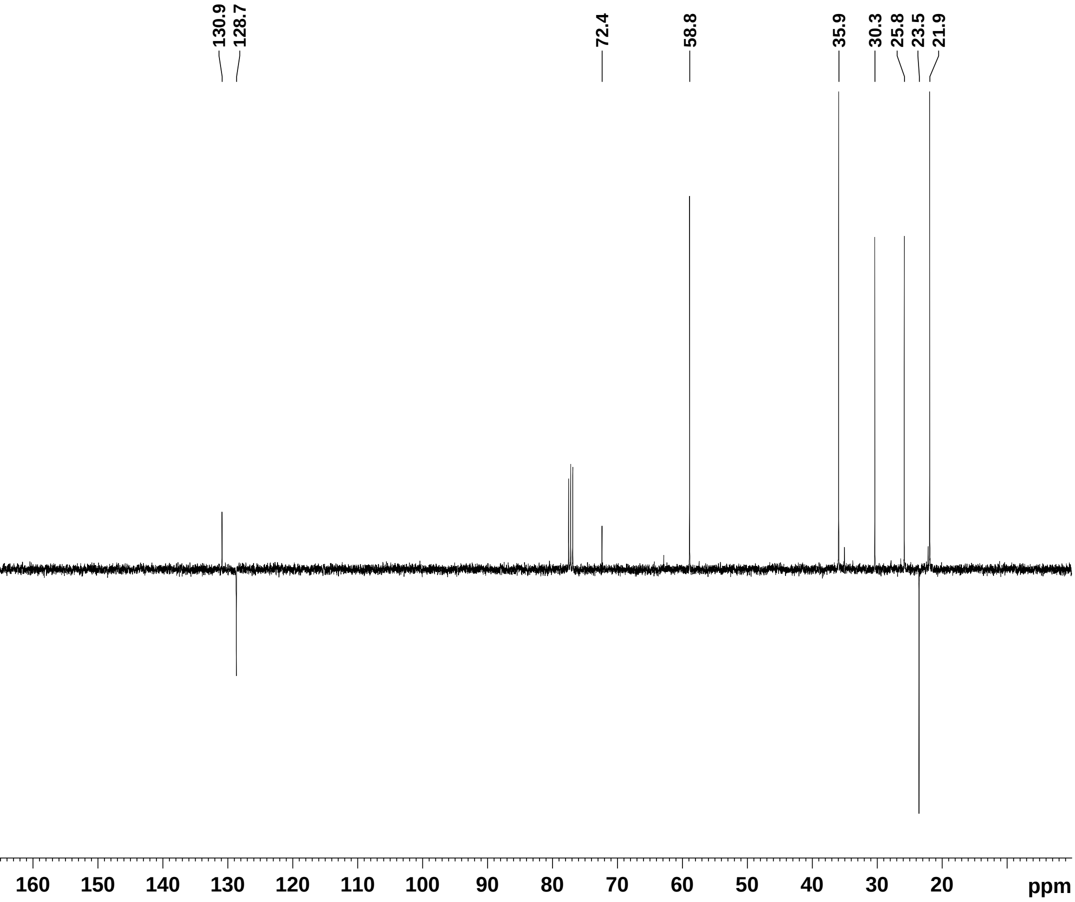

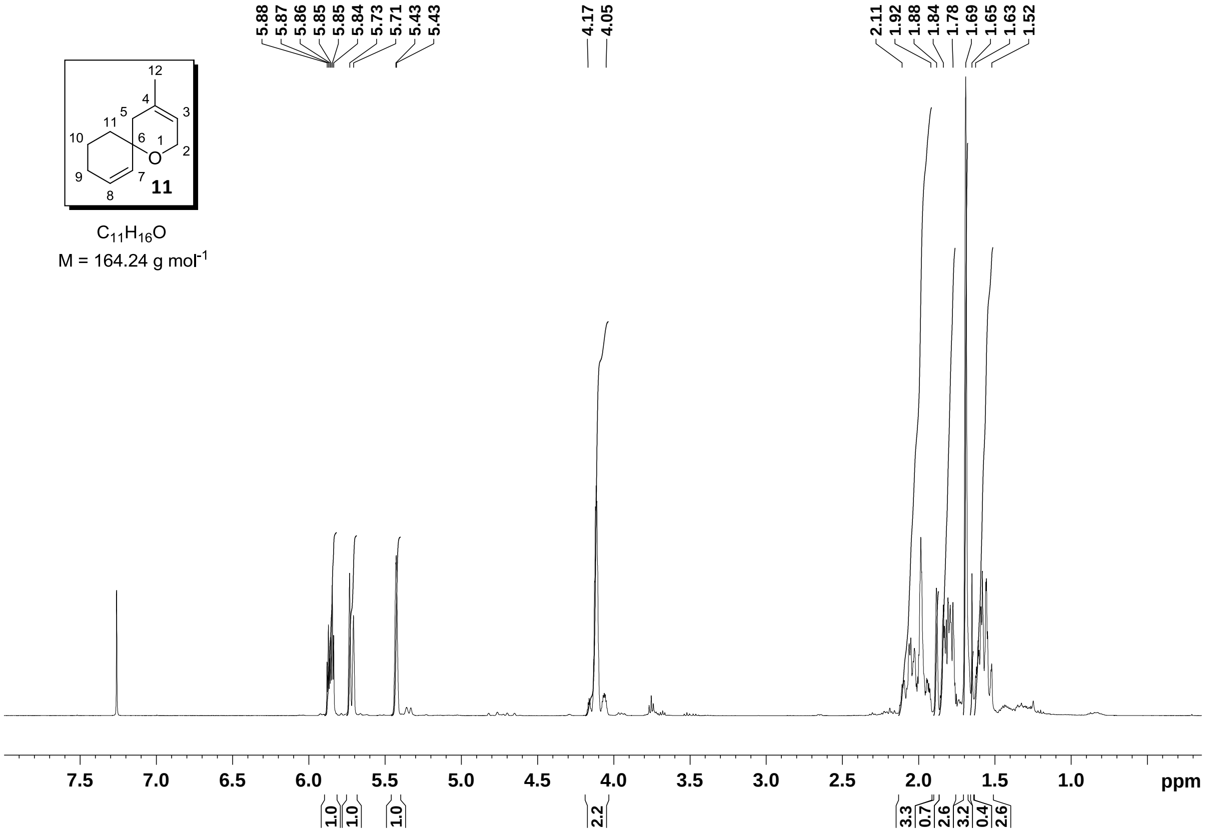

$C_{11}H_{16}O$

M = 164.24 g mol^{-1}

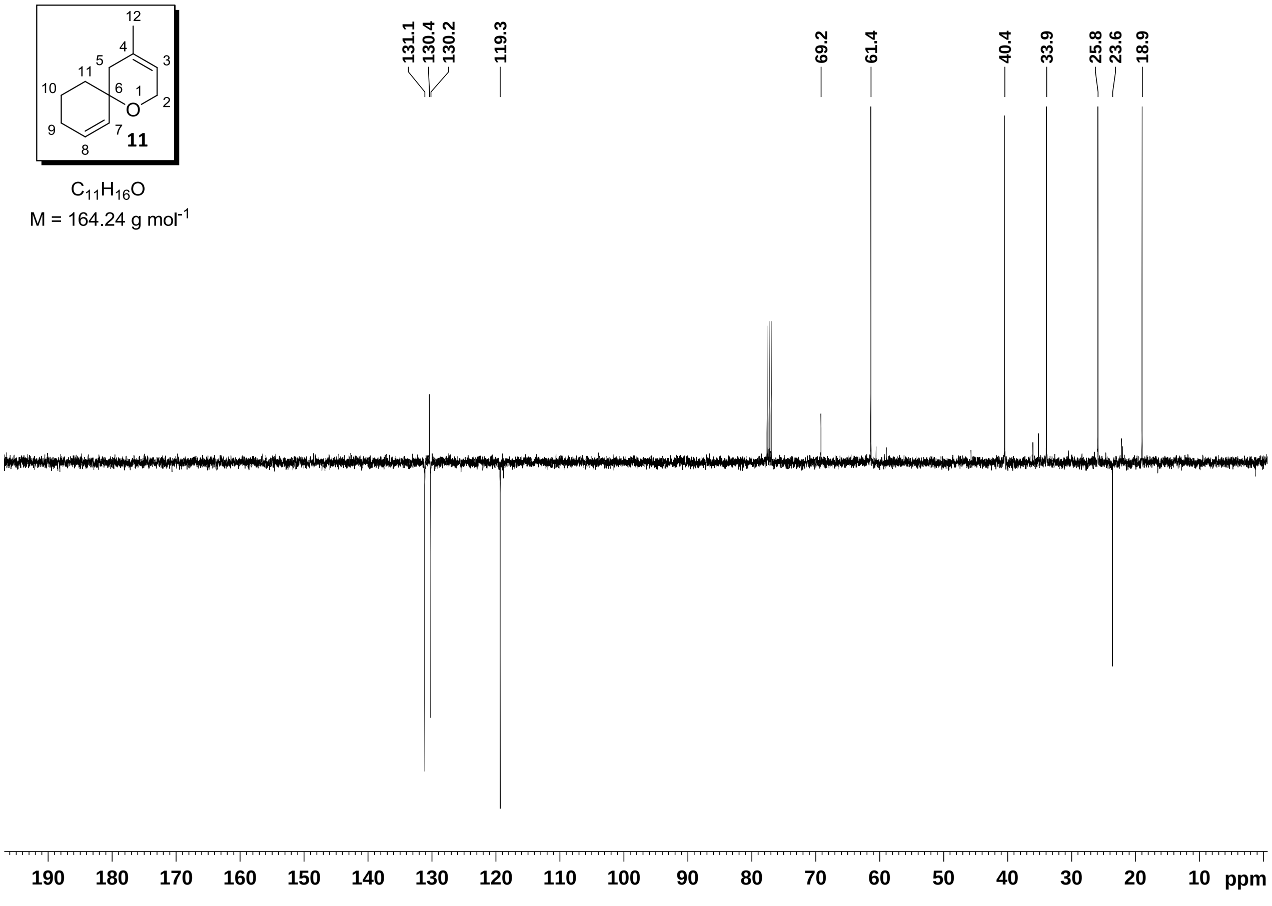

$C_{11}H_{16}O$

M = 164.24 g mol^{-1}

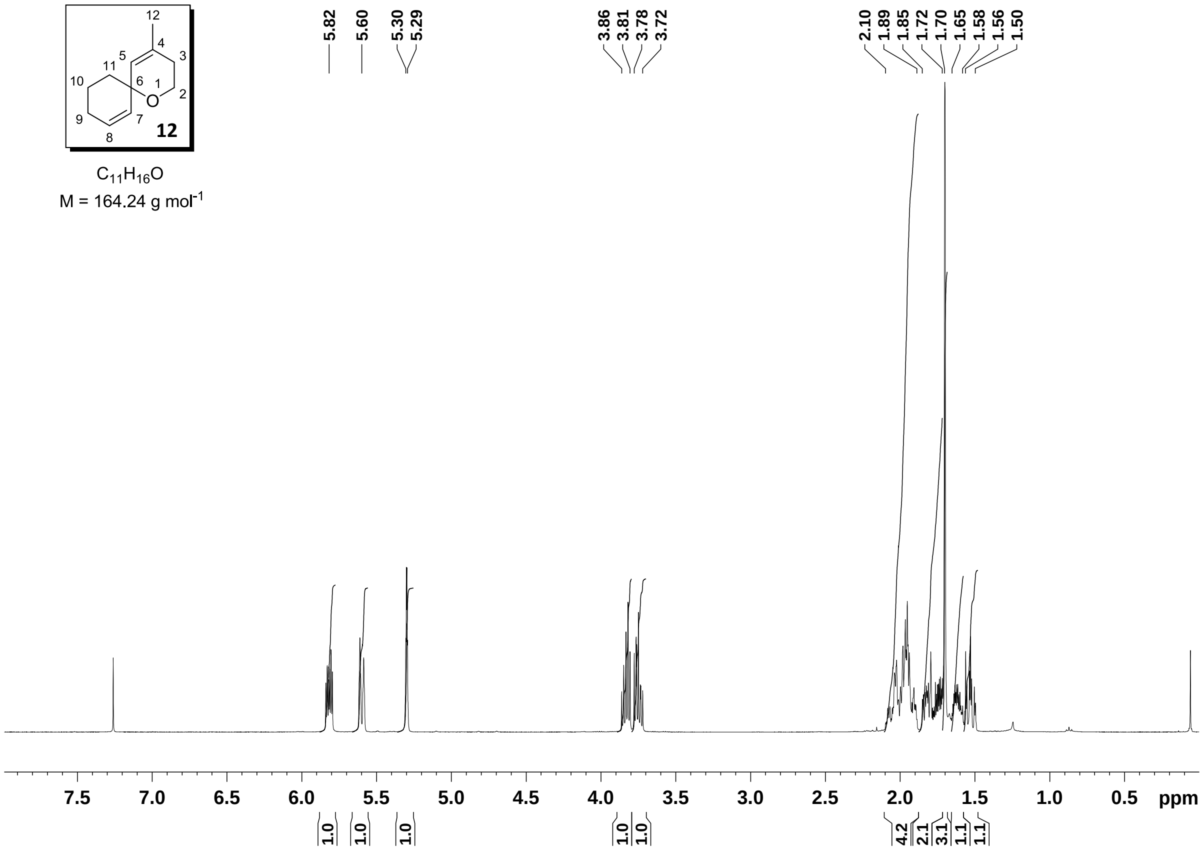
12
4
3
5
11
10
6
1
2
9
7
8
12
$C_{11}H_{16}O$
M = 164.24 g mol-1
5.82
5.60
5.30
5.29
3.86
3.81
3.78
3.72
2.10
1.89
1.85
1.72
1.70
1.65
1.58
1.56
1.50
7.5
7.0
6.5
6.0
5.5
5.0
4.5
4.0
3.5
3.0
2.5
2.0
1.5
1.0
0.5
ppm
1.0
1.0
1.0
1.0
1.0
4.2
2.1
3.1
1.1
1.1

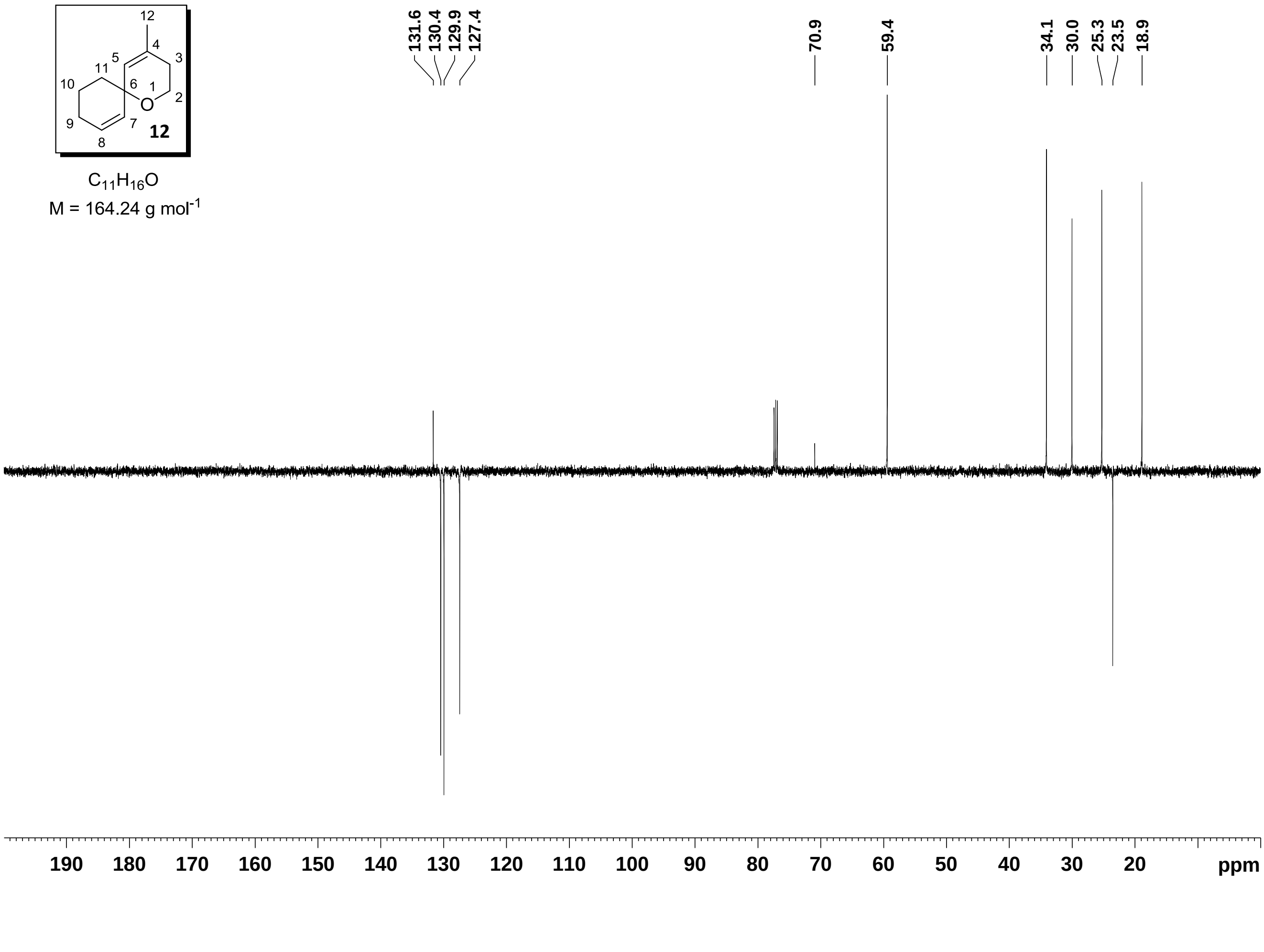
12
4
3
5
11
10
6
1
2
9
7
8
12
$C_{11}H_{16}O$
M = 164.24 g mol-1
131.6
130.4
129.9
127.4
70.9
59.4
34.1
30.0
25.3
23.5
18.9
190
180
170
160
150
140
130
120
110
100
90
80
70
60
50
40
30
20
ppm

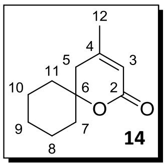

14

$C_{11}H_{16}O_2$

M = 180.24 g mol^{-1}

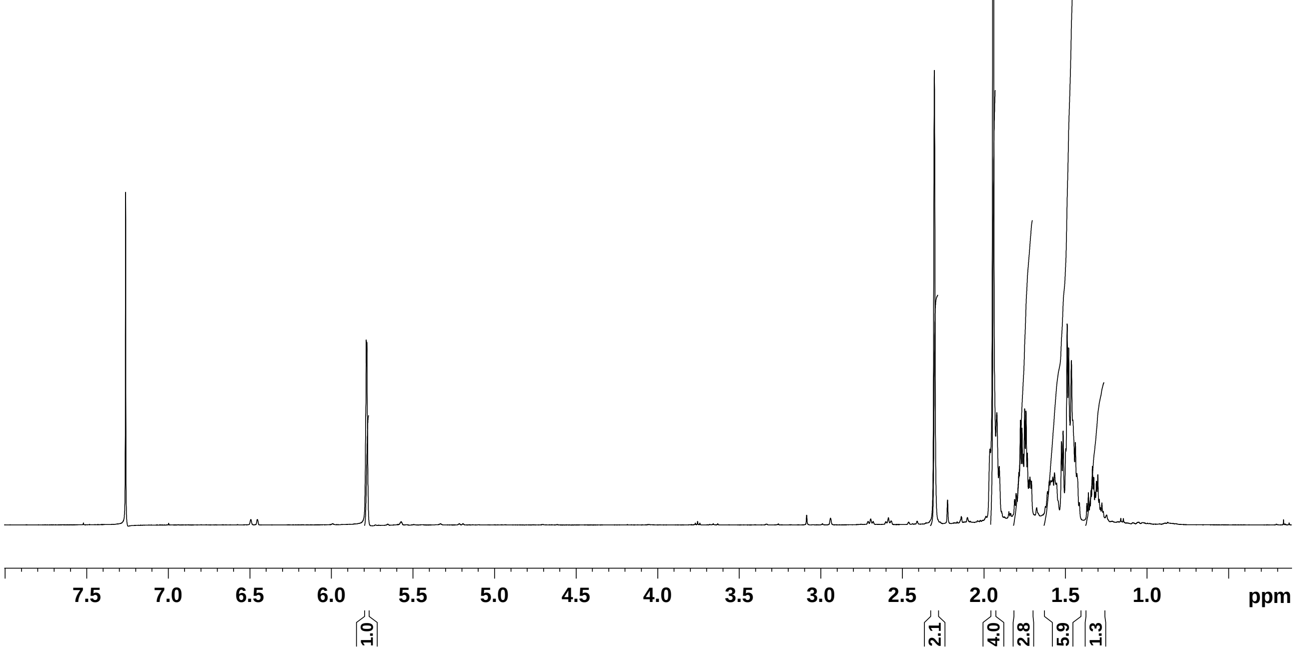

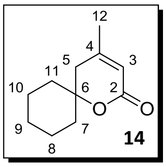

14

$C_{11}H_{16}O_2$

M = 180.24 g mol^{-1}

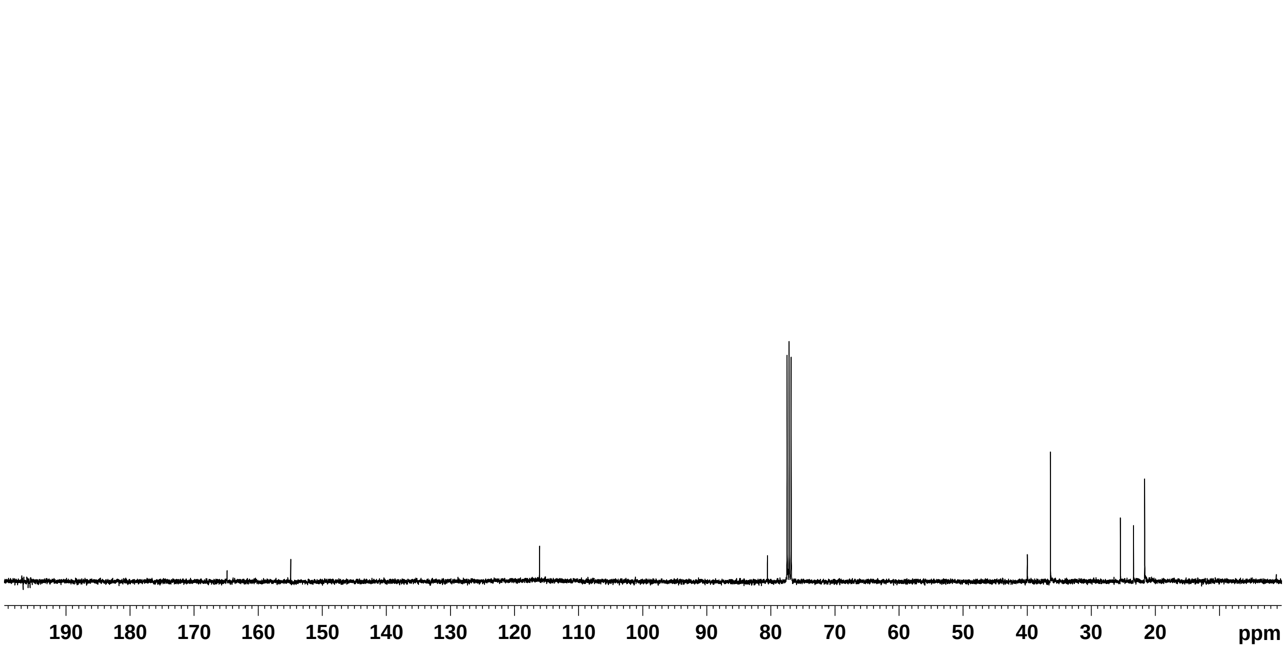

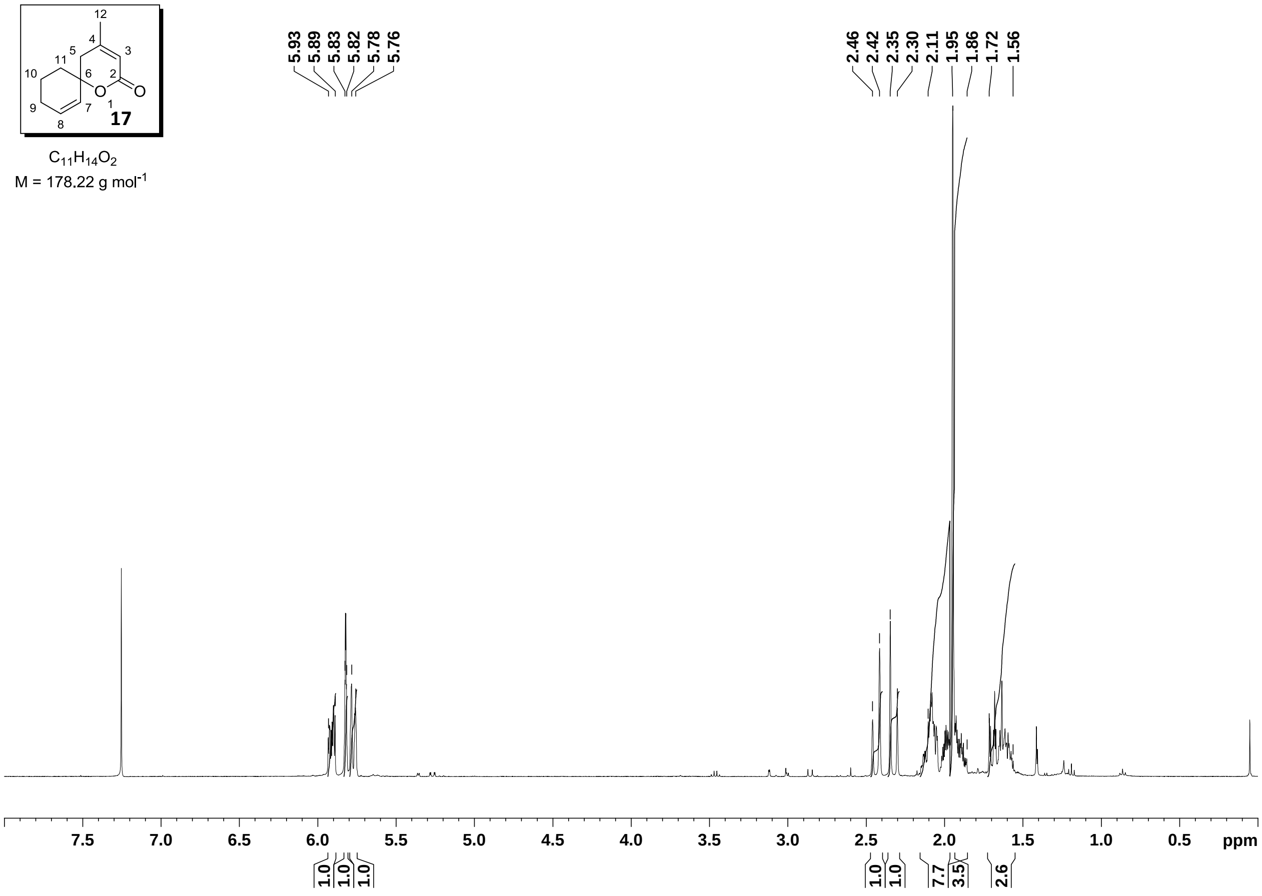
17
$C_{11}H_{14}O_2$
M = 178.22 g mol^{-1}
5.93 5.89 5.83 5.82 5.78 5.76
2.46 2.42 2.35 2.30 2.11 1.95 1.86 1.72 1.56
7.5 7.0 6.5 6.0 5.5 5.0 4.5 4.0 3.5 3.0 2.5 2.0 1.5 1.0 0.5 ppm
1.0 1.0 1.0 1.0 1.0 7.7 3.5 2.6

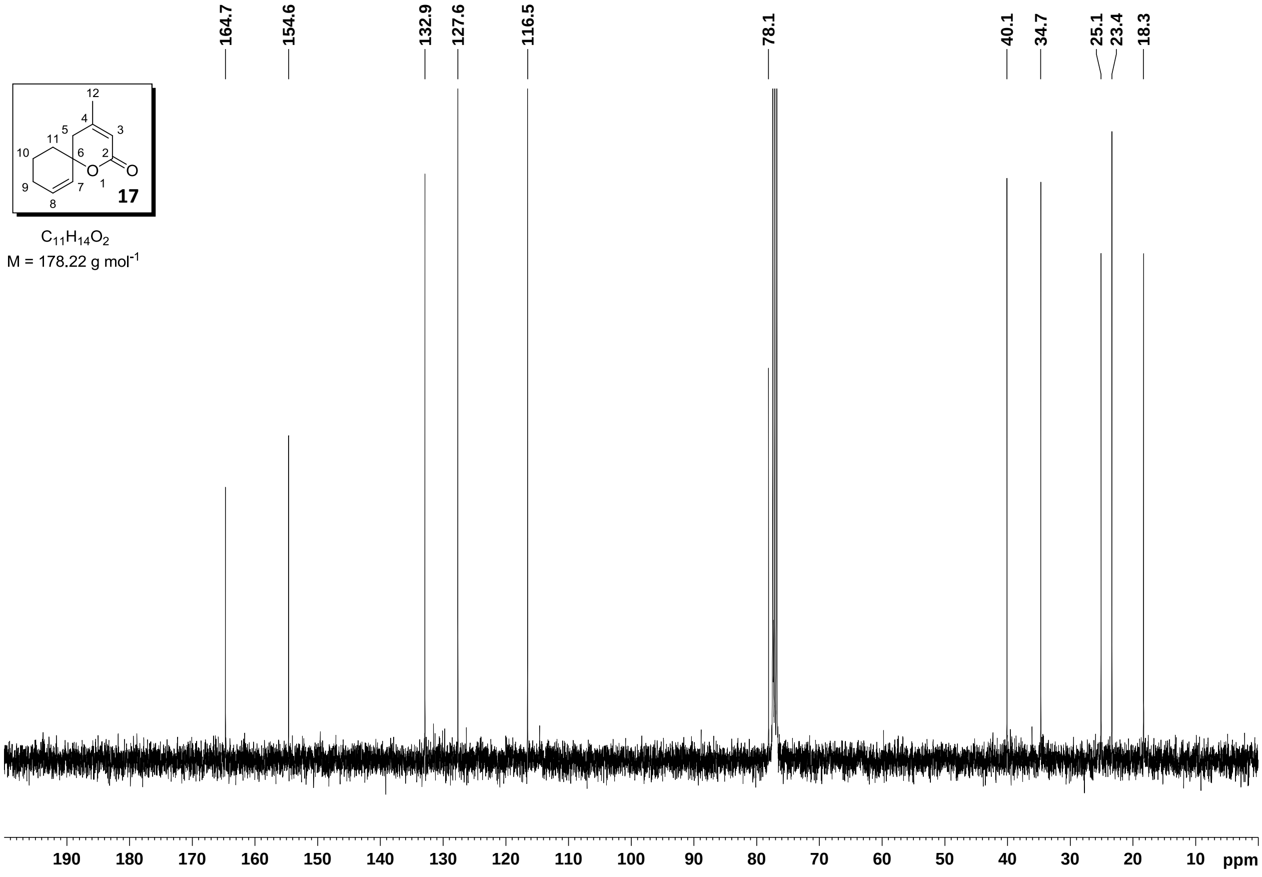
17
$C_{11}H_{14}O_2$
M = 178.22 g mol^{-1}
164.7 154.6 132.9 127.6 116.5 78.1 40.1 34.7 25.1 23.4 18.3
190 180 170 160 150 140 130 120 110 100 90 80 70 60 50 40 30 20 10 ppm

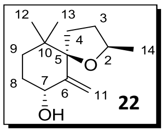

$C_{13}H_{22}O_2$

M = 210.31 g mol^{-1}

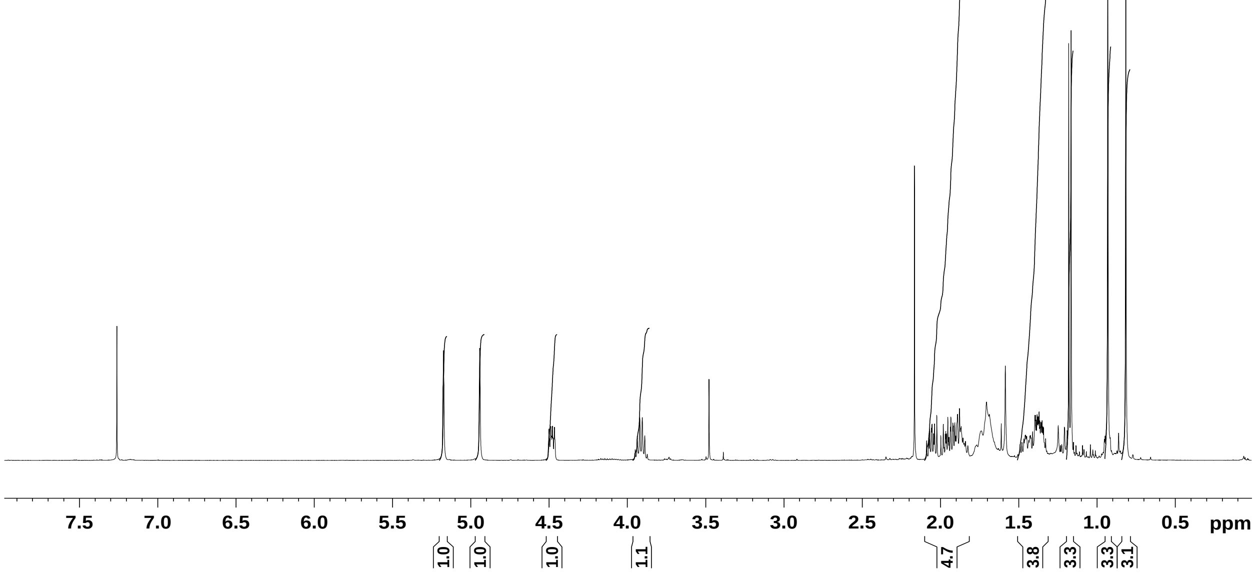

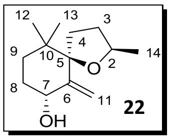

$C_{13}H_{22}O_2$

M = 210.31 g mol^{-1}

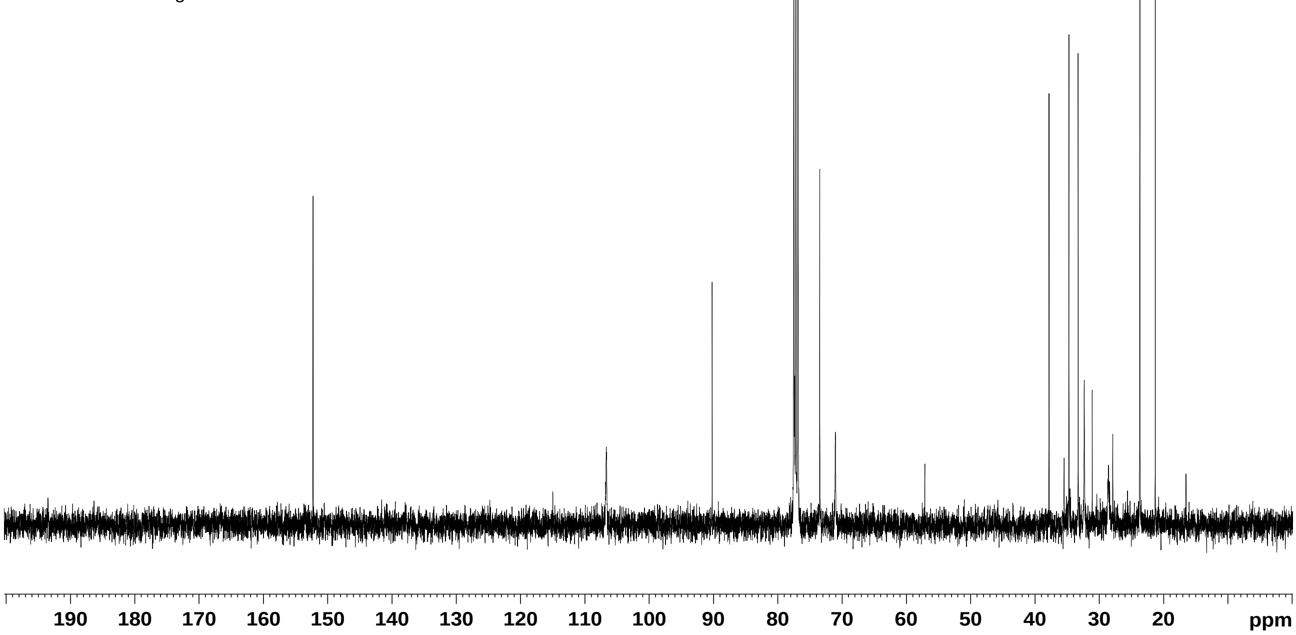

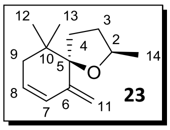

$C_{13}H_{20}O$

M = 192.30 g mol^{-1}

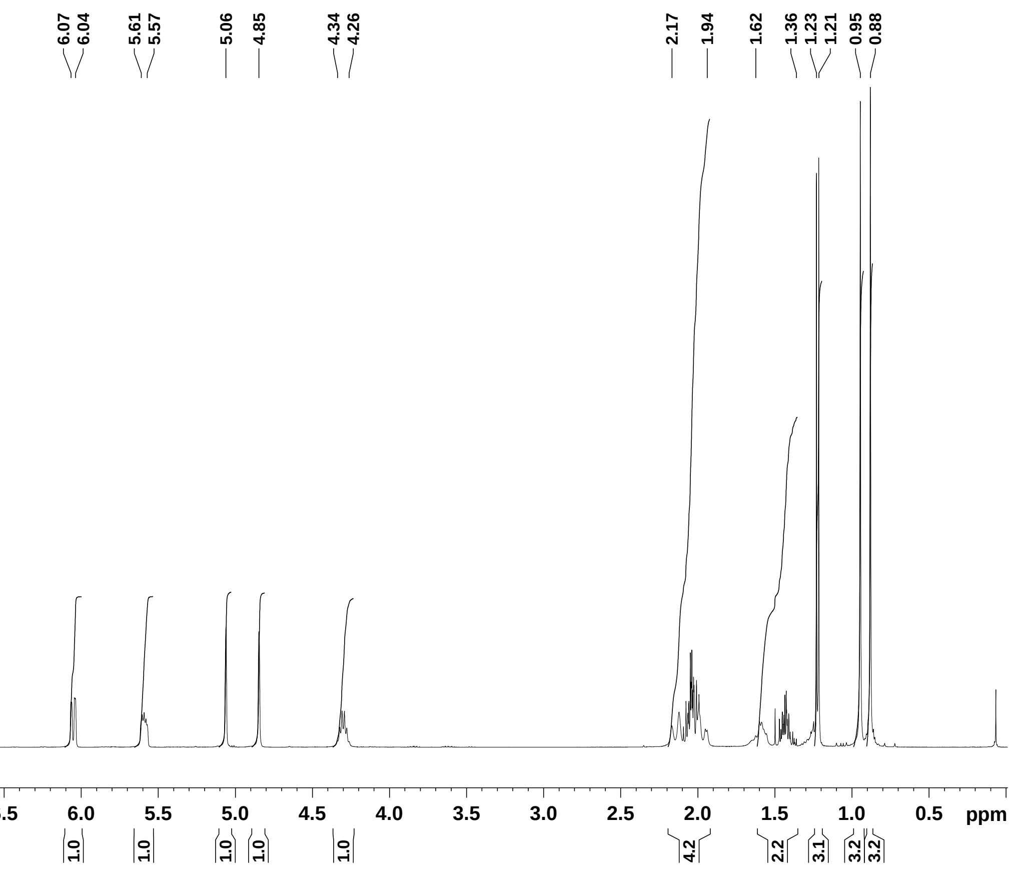

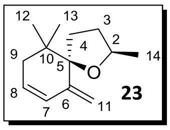

$C_{13}H_{20}O$

M = 192.30 g mol^{-1}

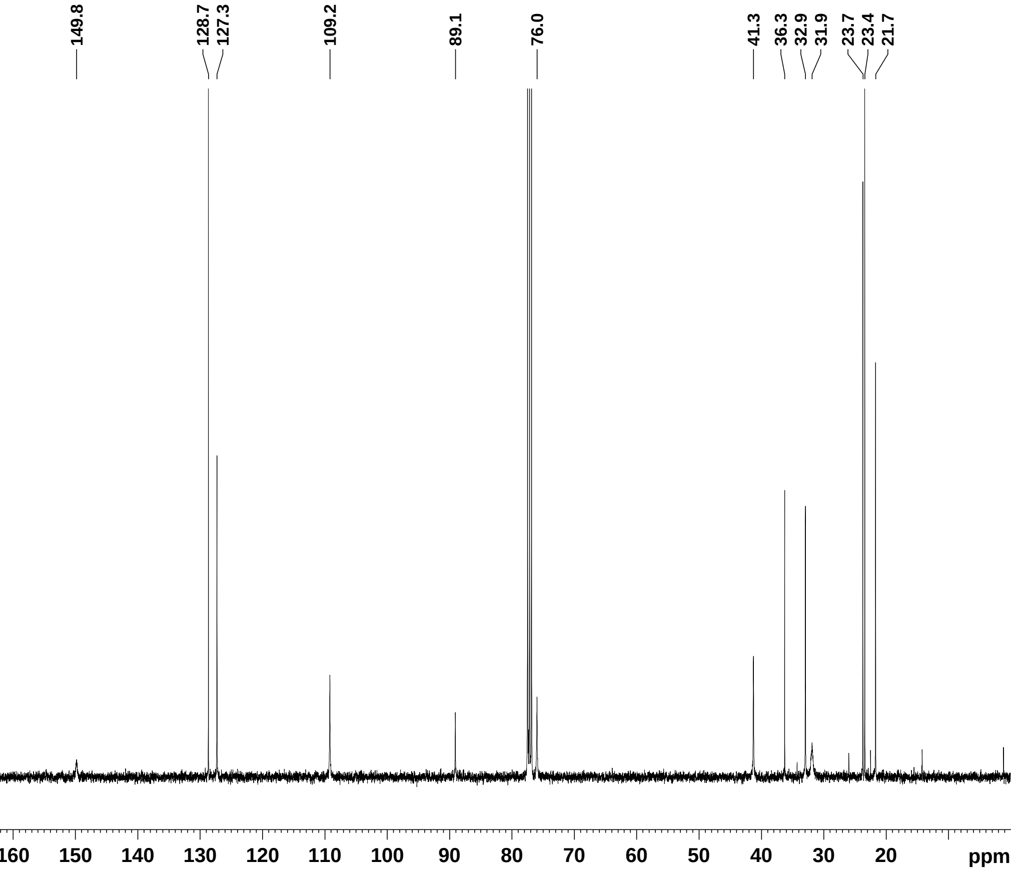

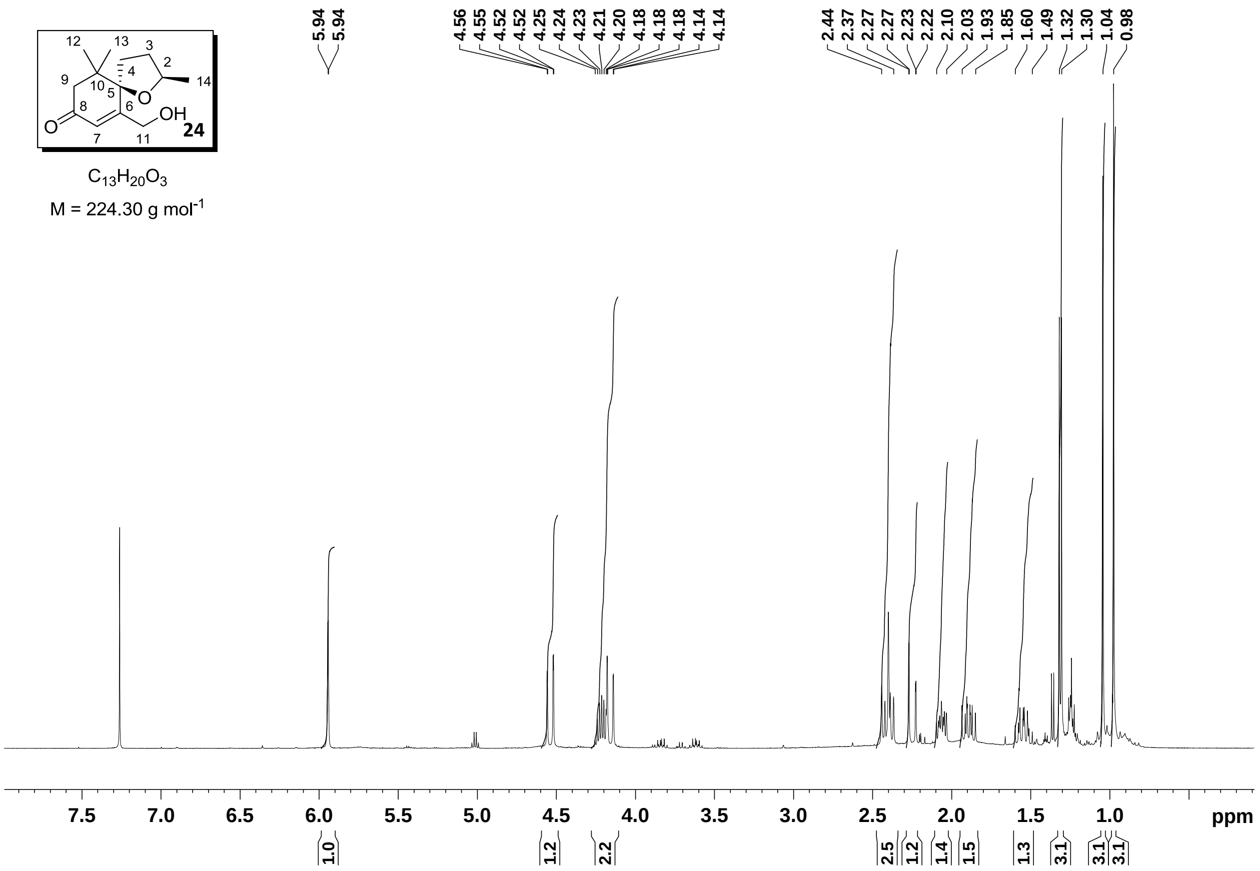
12
13
3
2
4
9
10
5
14
8
6
O
OH
O
7
11
24
C13H20O3
M = 224.30 g mol-1
5.94
5.94
4.56
4.55
4.52
4.52
4.25
4.24
4.23
4.21
4.20
4.18
4.18
4.18
4.14
4.14
2.44
2.37
2.27
2.27
2.23
2.22
2.10
2.03
1.93
1.85
1.60
1.49
1.32
1.30
1.04
0.98
7.5
7.0
6.5
6.0
5.5
5.0
4.5
4.0
3.5
3.0
2.5
2.0
1.5
1.0
ppm
1.0
1.2
2.2
2.5
1.2
1.4
1.5
1.3
3.1
3.1
3.1

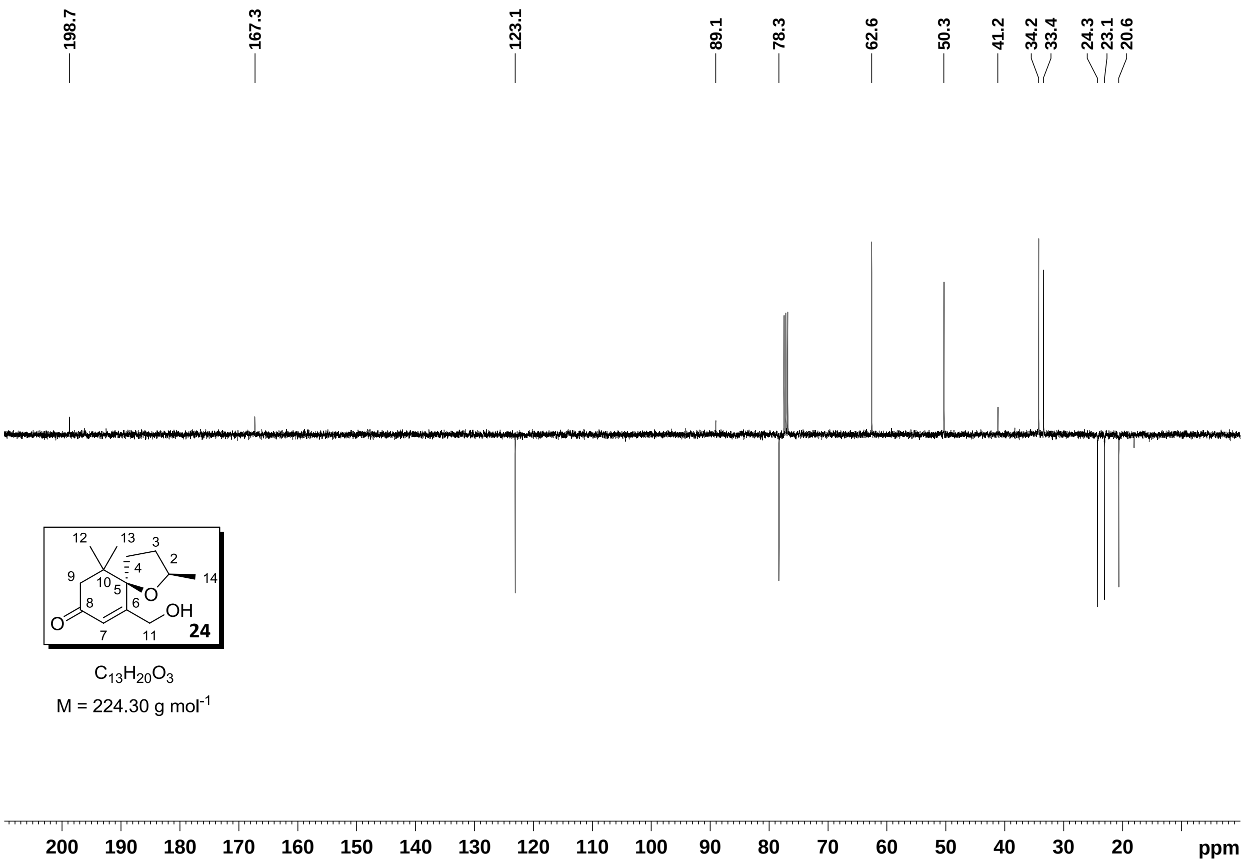
198.7
167.3
123.1
89.1
78.3
62.6
50.3
41.2
34.2
33.4
24.3
23.1
20.6
12
13
3
2
4
9
10
5
14
8
6
O
OH
O
7
11
24
C13H20O3
M = 224.30 g mol-1
200
190
180
170
160
150
140
130
120
110
100
90
80
70
60
50
40
30
20
ppm

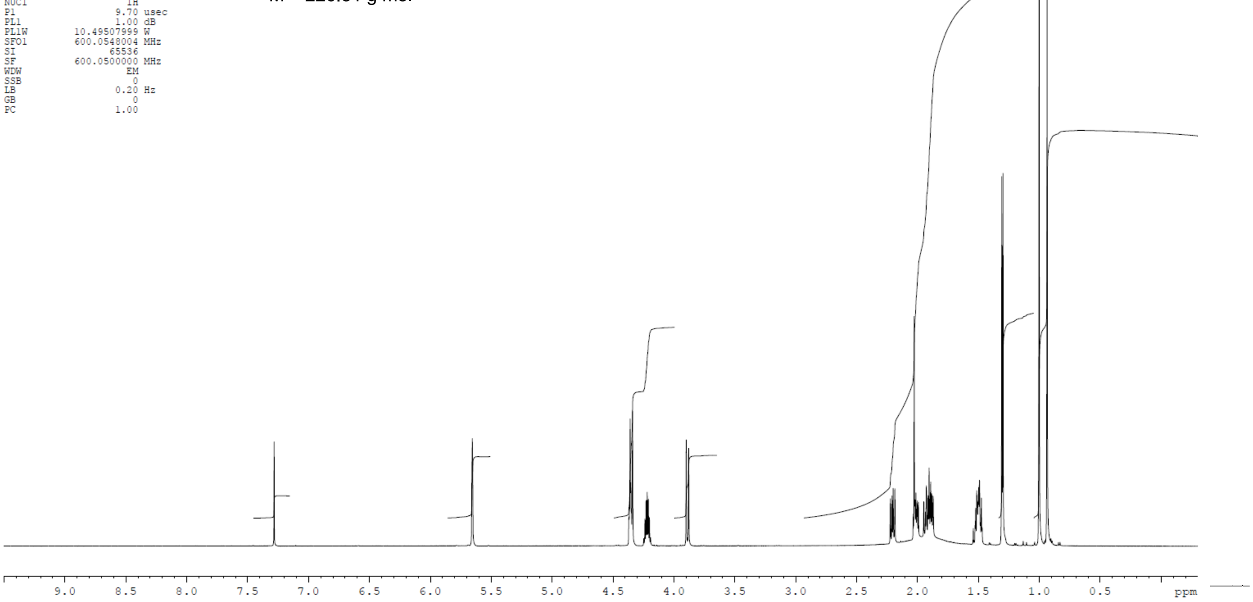

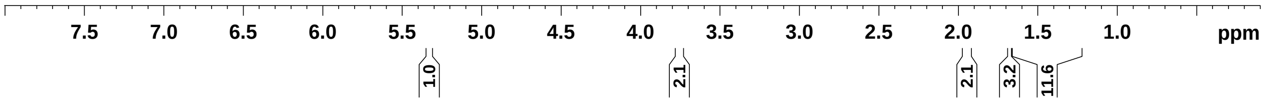

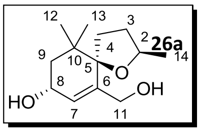

$C_{13}H_{22}O_3$

M = 226.31 g mol^{-1}

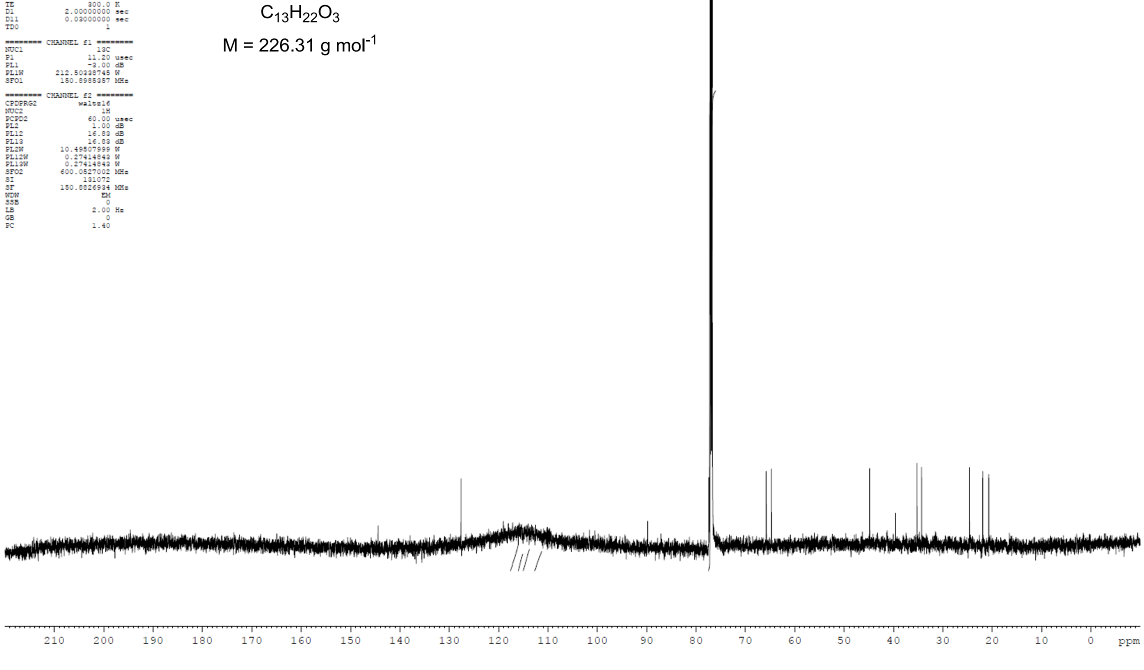

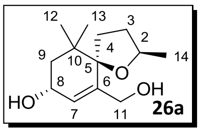

$C_{13}H_{22}O_3$

M = 226.31 g mol^{-1}

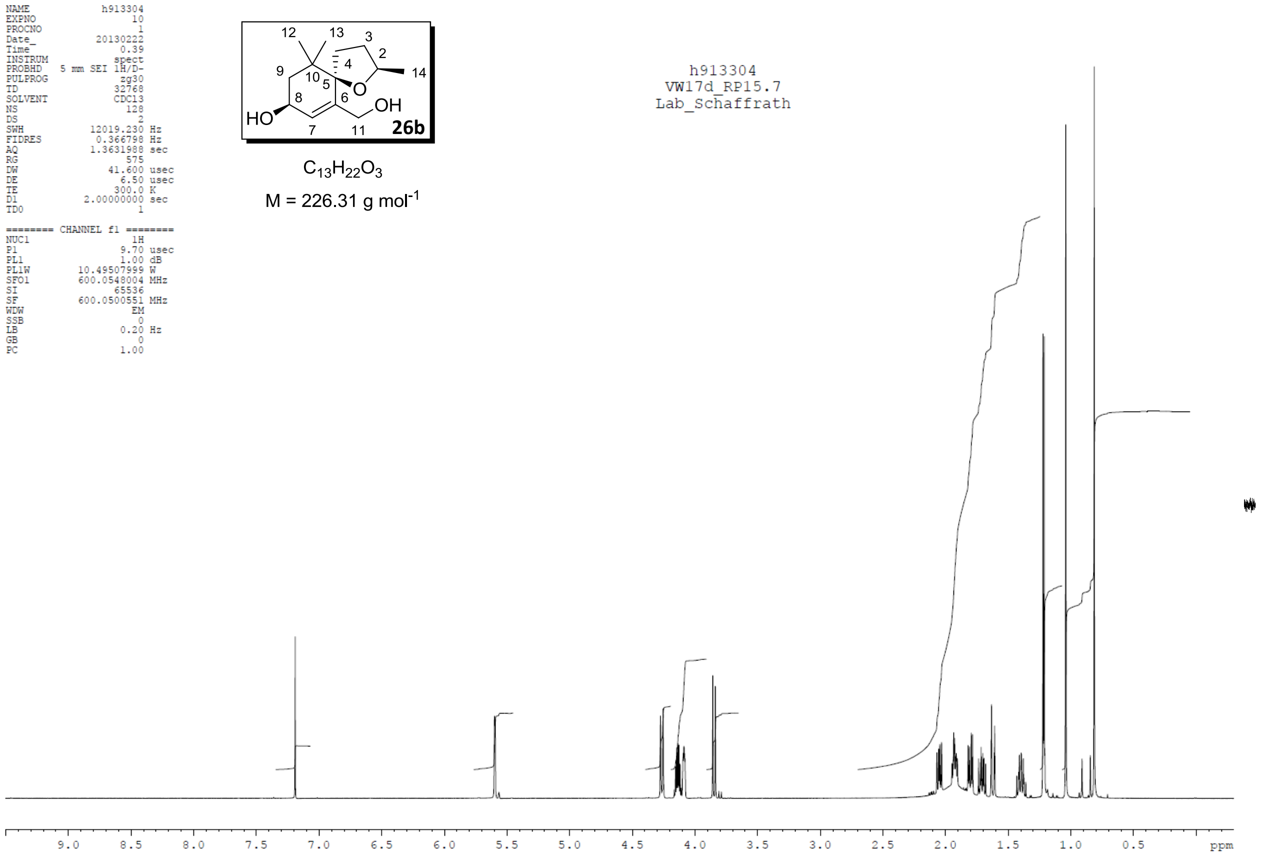
NAME h913304
EXPNO 10
PROCNO 1
Date_ 20130222
Time 0.39
INSTRUM spect
PROBHD 5 mm SEI 1H/D-
PULPROG zg30
TD 32768
SOLVENT CDCl3
NS 128
DS 2
SWH 12019.230 Hz
FIDRES 0.366798 Hz
AQ 1.3631988 sec
RG 575
DW 41.600 usec
DE 6.50 usec
TE 300.0 K
D1 2.00000000 sec
TD0 1
======== CHANNEL f1 ========
NUC1 1H
P1 9.70 usec
PL1 1.00 dB
PL1W 10.49507999 W
SFO1 600.0548004 MHz
SI 65536
SF 600.0500551 MHz
WDW EM
SSB 0
LB 0.20 Hz
GB 0
PC 1.00
12
13
3
2
4
9
10
5
14
O
8
6
OH
HO
7
11
26b
$C_{13}H_{22}O_3$
M = 226.31 g mol^{-1}
h913304
VW17d_RP15.7
Lab_Schaffrath
9.0
8.5
8.0
7.5
7.0
6.5
6.0
5.5
5.0
4.5
4.0
3.5
3.0
2.5
2.0
1.5
1.0
0.5
ppm

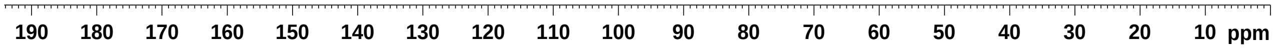
190
180
170
160
150
140
130
120
110
100
90
80
70
60
50
40
30
20
10
ppm

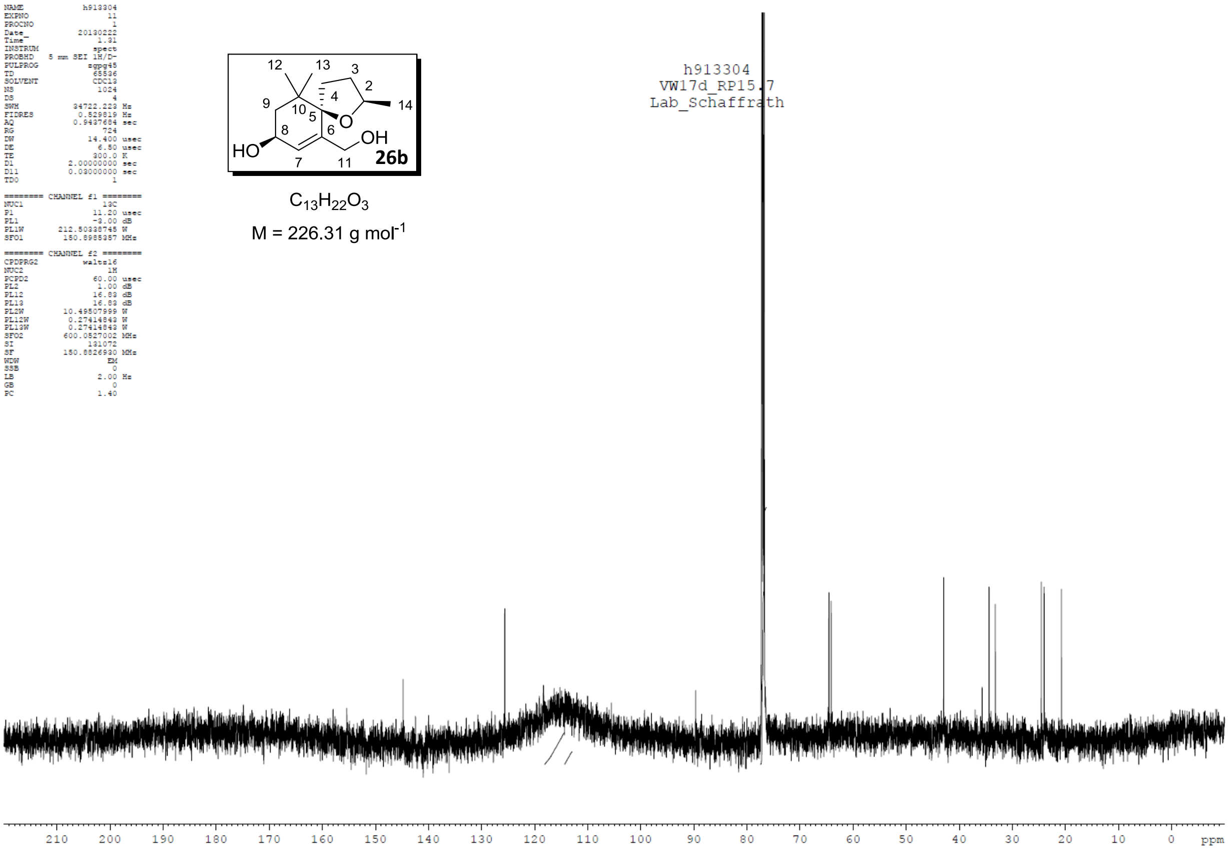
NAME h913304
EXPNO 11
PROCNO 1
Date_ 20130222
Time 1.31
INSTRUM spect
PROBHD 5 mm SEI 1H/D-
PULPROG zgpg45
TD 65536
SOLVENT CDCl3
NS 1024
DS 4
SWH 34722.223 Hz
FIDRES 0.529819 Hz
AQ 0.9437684 sec
RG 724
DW 14.400 usec
DE 6.50 usec
TE 300.0 K
D1 2.00000000 sec
D11 0.03000000 sec
TD0 1
======== CHANNEL f1 ========
NUC1 13C
P1 11.20 usec
PL1 -3.00 dB
PL1W 212.50338745 W
SFO1 150.8985357 MHz
======== CHANNEL f2 ========
CPDPRG2 waltz16
NUC2 1H
PCPD2 60.00 usec
PL2 1.00 dB
PL12 16.83 dB
PL13 16.83 dB
PL2W 10.49507999 W
PL12W 0.27414843 W
PL13W 0.27414843 W
SFO2 600.0527002 MHz
SI 131072
SF 150.8826930 MHz
WDW EM
SSB 0
LB 2.00 Hz
GB 0
PC 1.40
12
13
3
2
4
9
10
5
14
O
8
6
OH
HO
7
11
26b
$C_{13}H_{22}O_3$
M = 226.31 g mol^{-1}
h913304
VW17d_RP15.7
Lab_Schaffrath
210
200
190
180
170
160
150
140
130
120
110
100
90
80
70
60
50
40
30
20
10
0
ppm

HPLC analysis, Methods

Quality control of the compounds: All measurements were performed on an analytical Waters H-class UPLC system with a quadrupol mass and a PDA detector. For the reversed phase method, a Waters Sunfire C18 phase was used as stationary phase. Mobil phases were H_2O/MeCN gradients with 0.05% TFA as modifier.

Separation of enantiomeric mixtures: The measurements were performed with analytical Waters Alliance HPLC systems with PDA and chiral detectors. For the normal phase method development several commercial and non-commercial (not listed) amylose and cellulose derivatized chiral stationary phases (CSP) were available. Amylose: Daicel Chiralpak AD-H, AS-H, IA, IB, IF, AY, Phenomenex Lux Amylose-2; Cellulose: Daicel Chiralcel OD-H, OJ-H, IC, OZ-H, Phenomenex Lux Cellulose-4. As mobile phase different mixtures of MeOH, EtOH, *iso*-PrOH, MeCN and heptane were used. Modifier was TFA or diethylamine (DEA).

Separation of the diastereomers: The reversed phase method development was performed with an analytical Waters H-class UPLC system with a quadrupol mass and a PDA detector. The preparative separation was carried out by a preparative Jasco HPLC with an UV detector. Mobil phase: MeCN/H_2O gradient, flow rate 30 mL/min, stationary phase: Waters Sunfire C18.

Chromatographic data

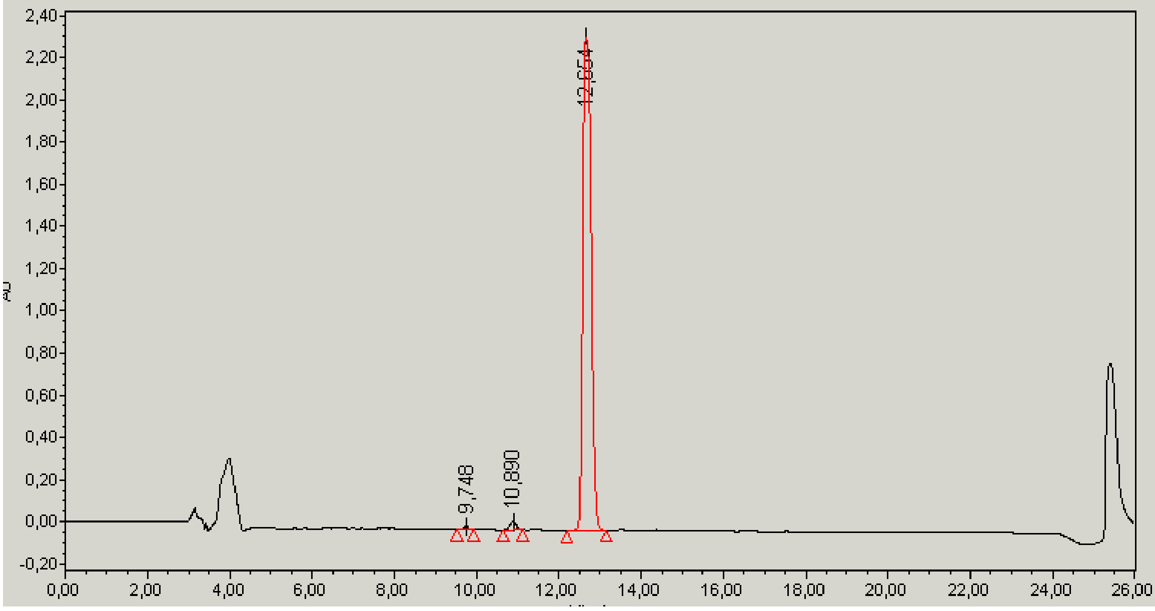

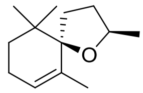

HPLC-chromatogram of theaspiran *trans*-**1**, Waters Sunfire 5 µm 250*4.6 mm, flow: 1 mL/min, UV detection 210 nm, gradient 90–95% MeCN in H_2O (0.05% TFA).

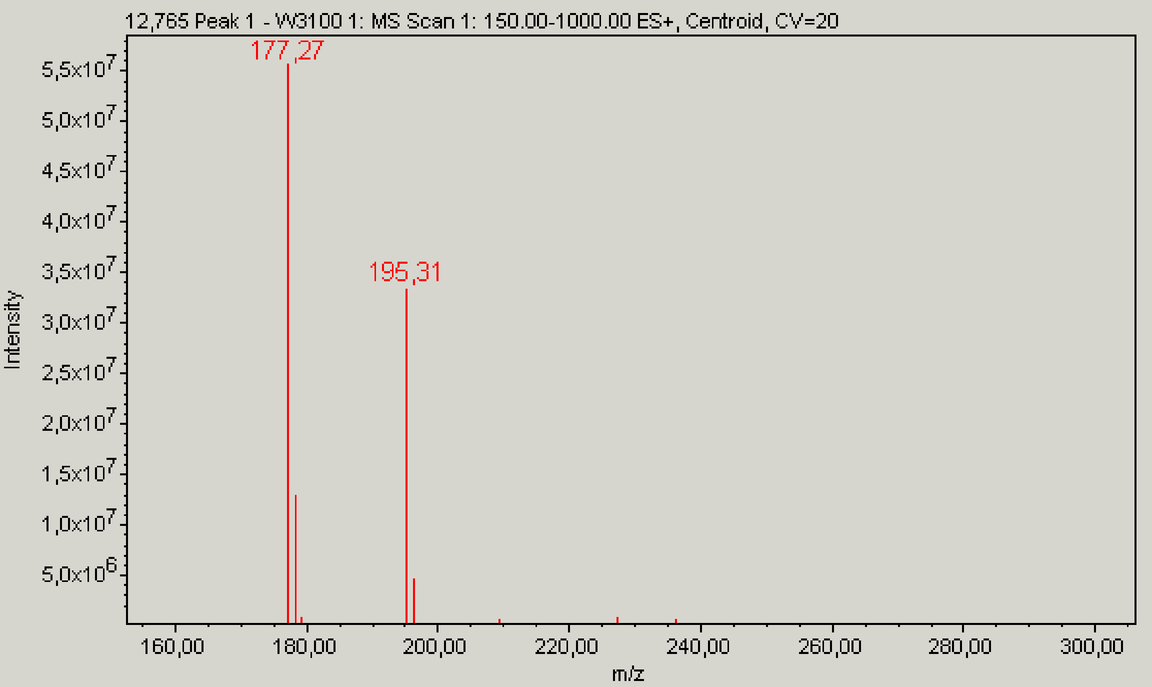

ESI-MS of theaspiran *trans*-**1**.

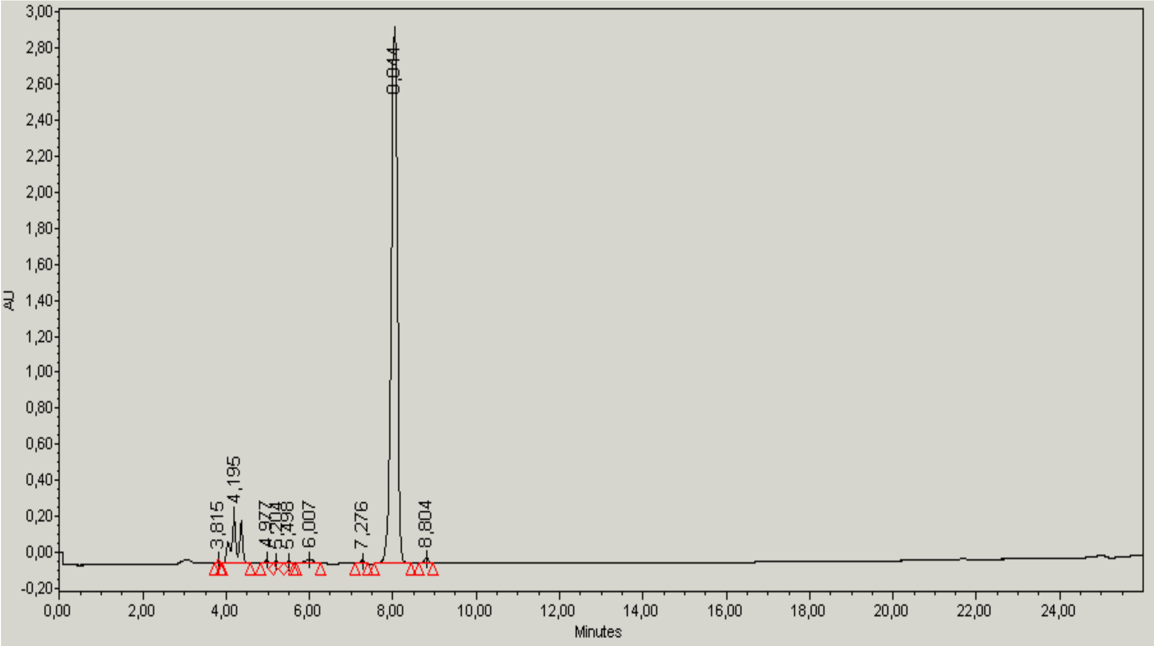

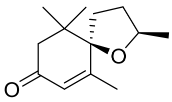

HPLC-chromatogram of enone **2**, Waters Sunfire 5 µm 250*4.6 mm, flow: 1 mL/min, UV detection 239 nm, gradient 50–90% MeCN in H_2O (0.05% TFA).

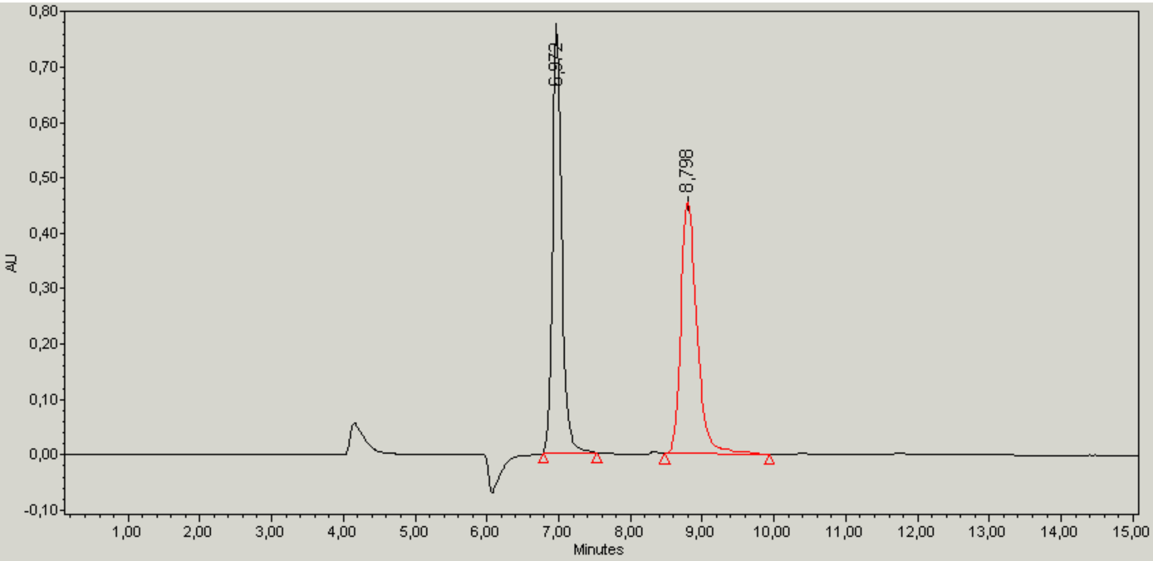

HPLC-chromatogram of enone **2** on a chiral stationary phase, Chiralpak IA 5 µm 250*4.6 mm, flow: 1 mL/min, UV detection 230 nm, isocratic heptane/EtOH/MeOH 50/1/1 + 0.1% TFA.

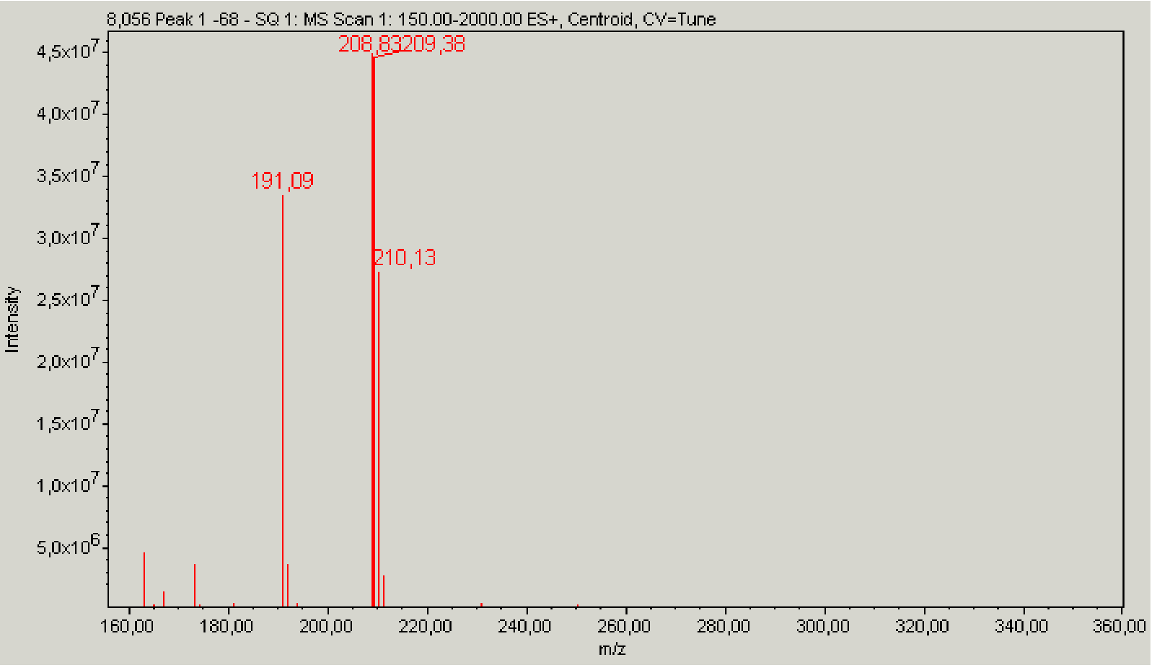

ESI-MS of enone **2**.

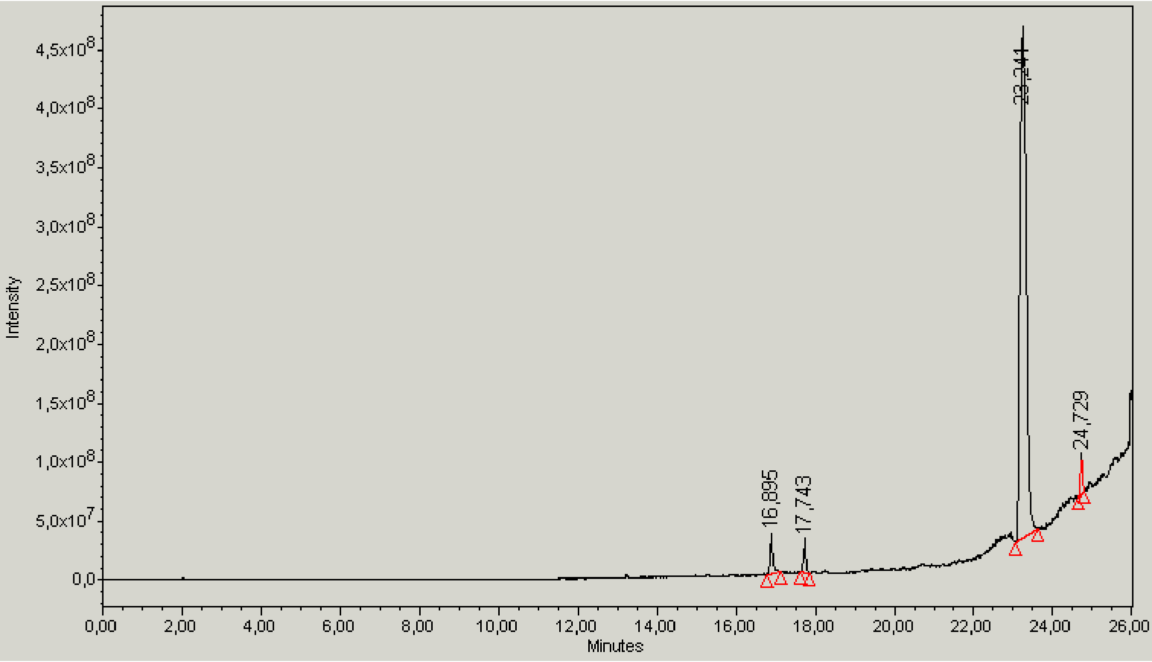

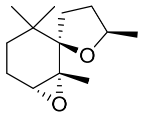

HPLC-chromatogram of epoxide **4a**, Waters Sunfire 5 µm 250*4.6 mm, flow: 1 mL/min, TIC, gradient 10–90% MeCN in H_2O (0.05% TFA).

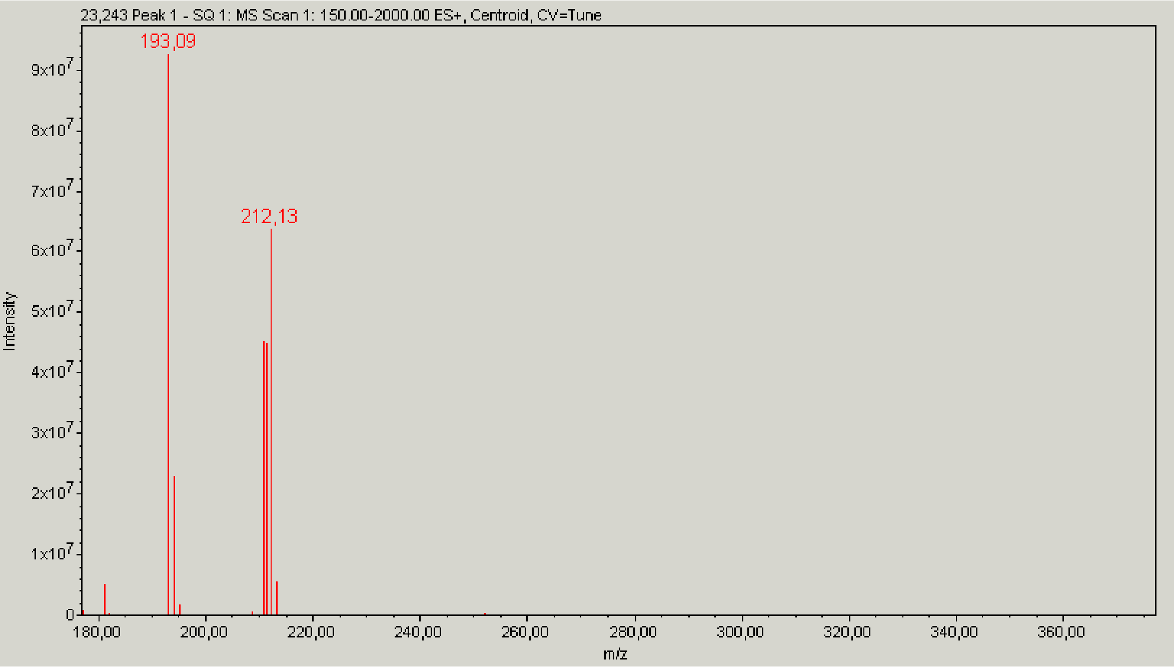

ESI-MS of epoxide **4a.**

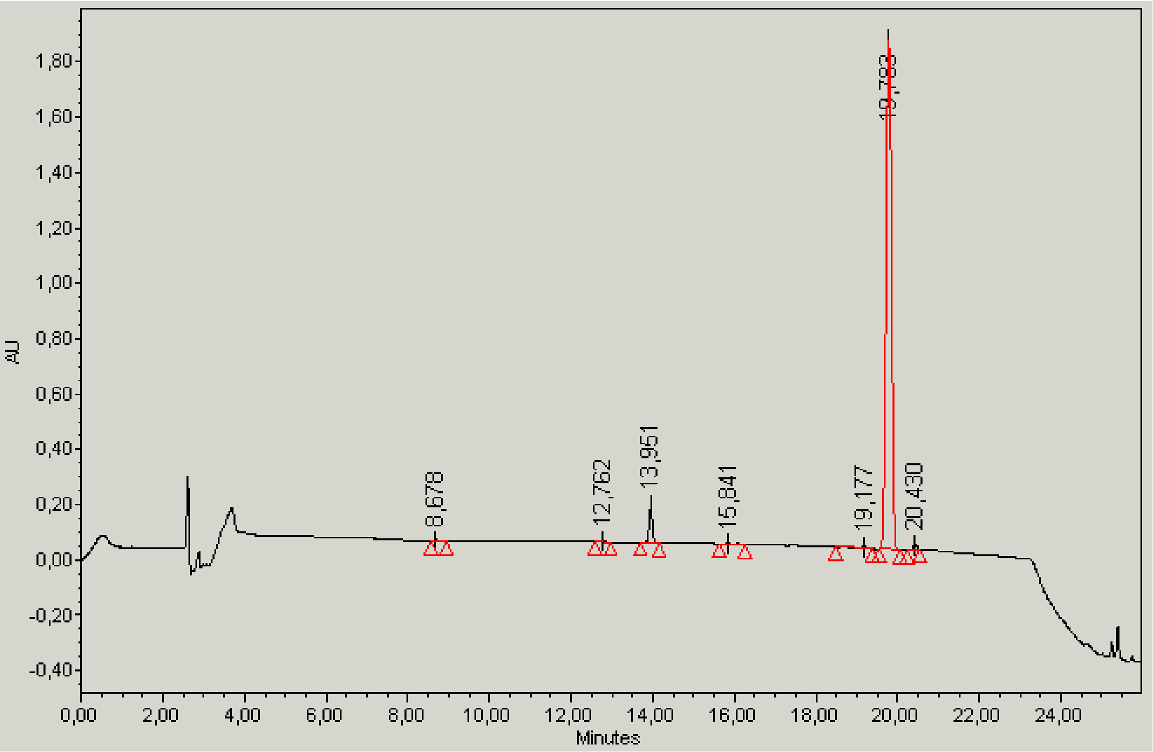

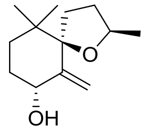

HPLC-chromatogram of allyl alcohol **22**, Waters Sunfire 5 µm 250*4.6 mm, flow: 1 mL/min, UV detection 238 nm, gradient 30–70% MeCN in H_2O (0.05% TFA).

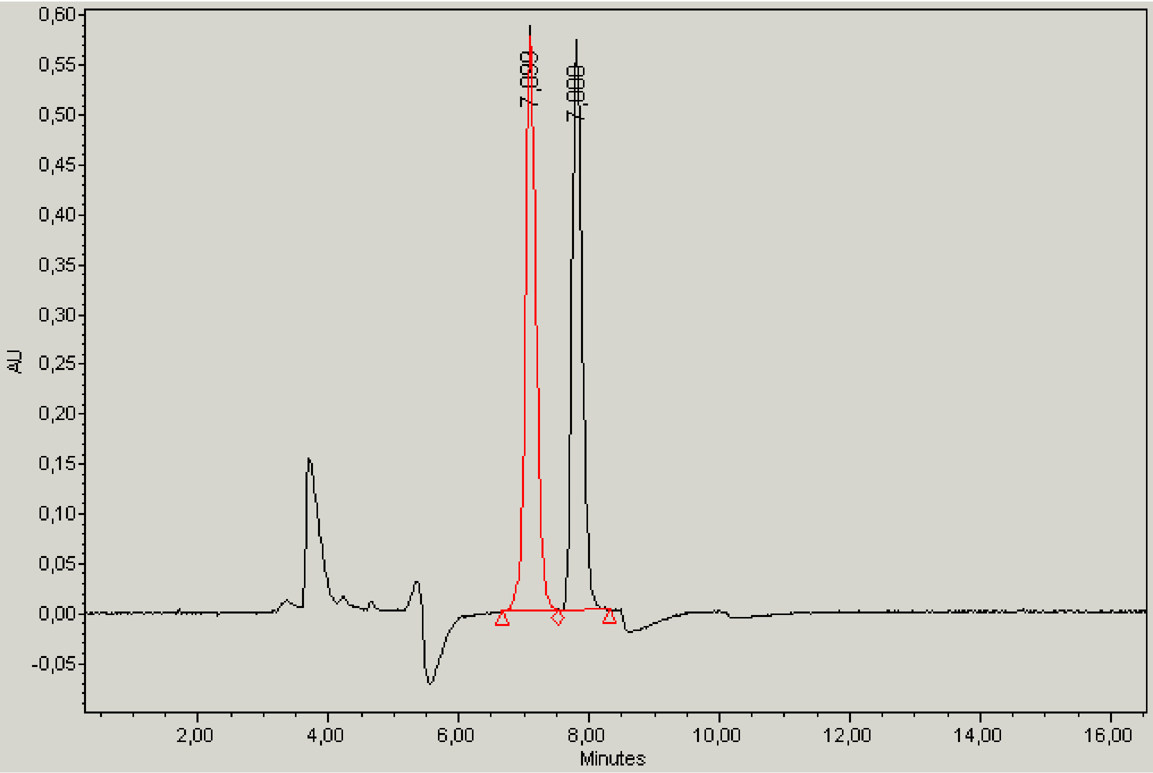

HPLC-chromatogram of allyl alcohol **22** on a chiral stationary phase, Chiralpak OD 5 µm 250*4.6 mm, flow: 1 mL/min, UV detection 202 nm, isocratic heptane/iPrOH 50/1 + 0.1% TFA.

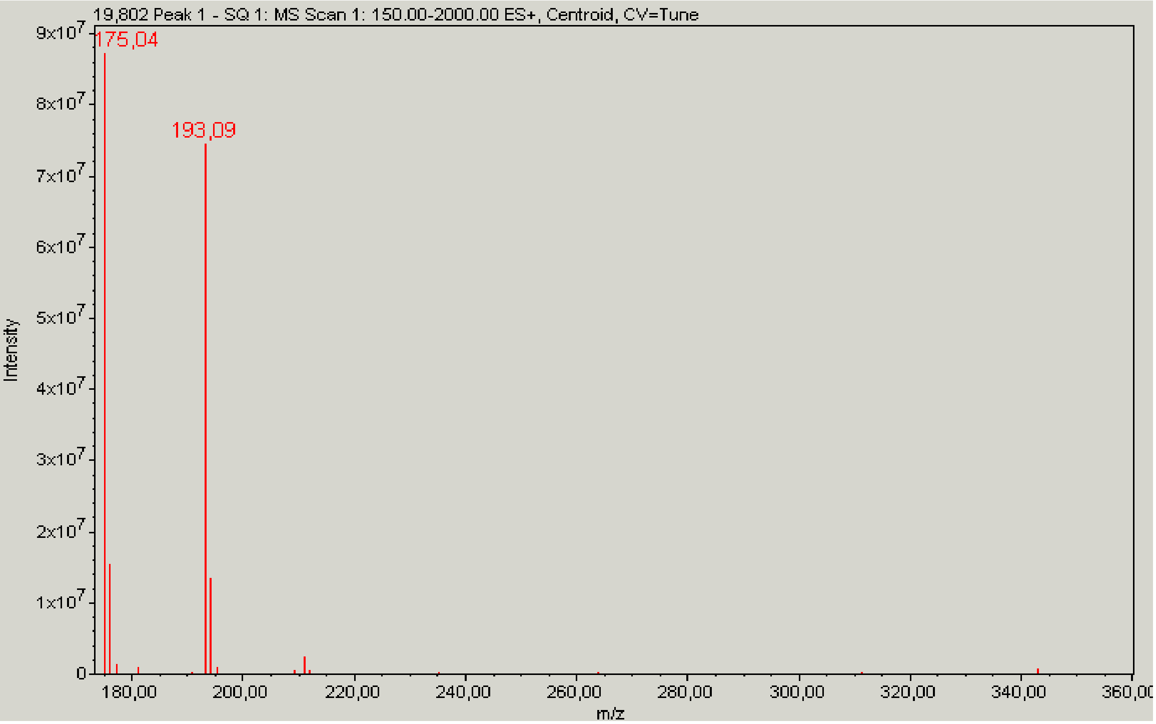

ESI-MS of allyl alcohol **22**.

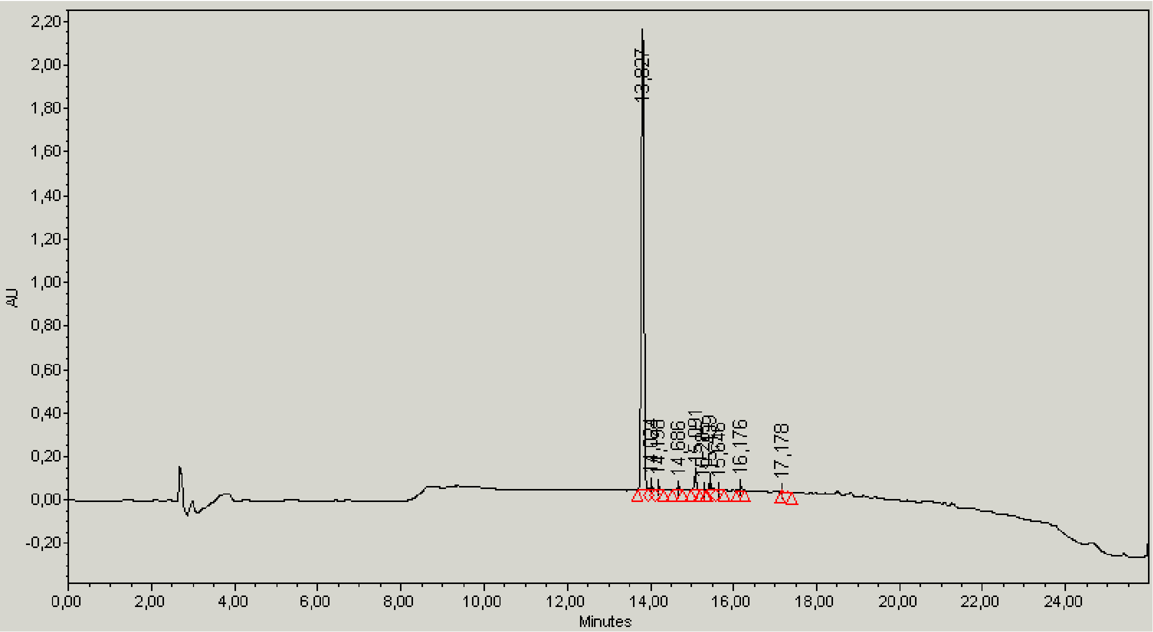

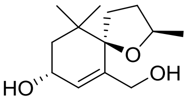

HPLC-chromatogram of diol **26a**, Waters Sunfire 5 µm 250*4.6 mm, flow: 1mL/min, UV detection 208 nm, gradient 10-90% MeCN in H_2O (0.05% TFA).

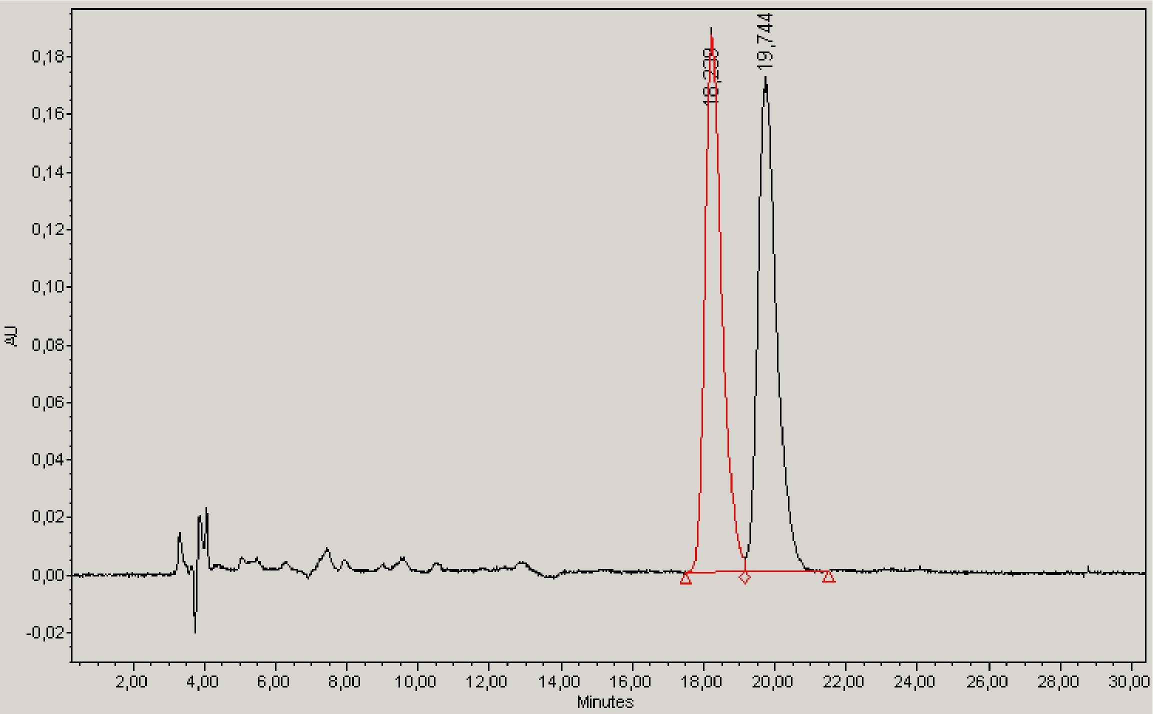

HPLC-chromatogram of diol **26a** on a chiral stationary phase, Chiralpak IC 5 µm 250*4.6 mm, flow: 1 mL/min, UV detection 202 nm, isocratic heptane/EtOH/MeOH 40/1/1 + 0.1% TFA.

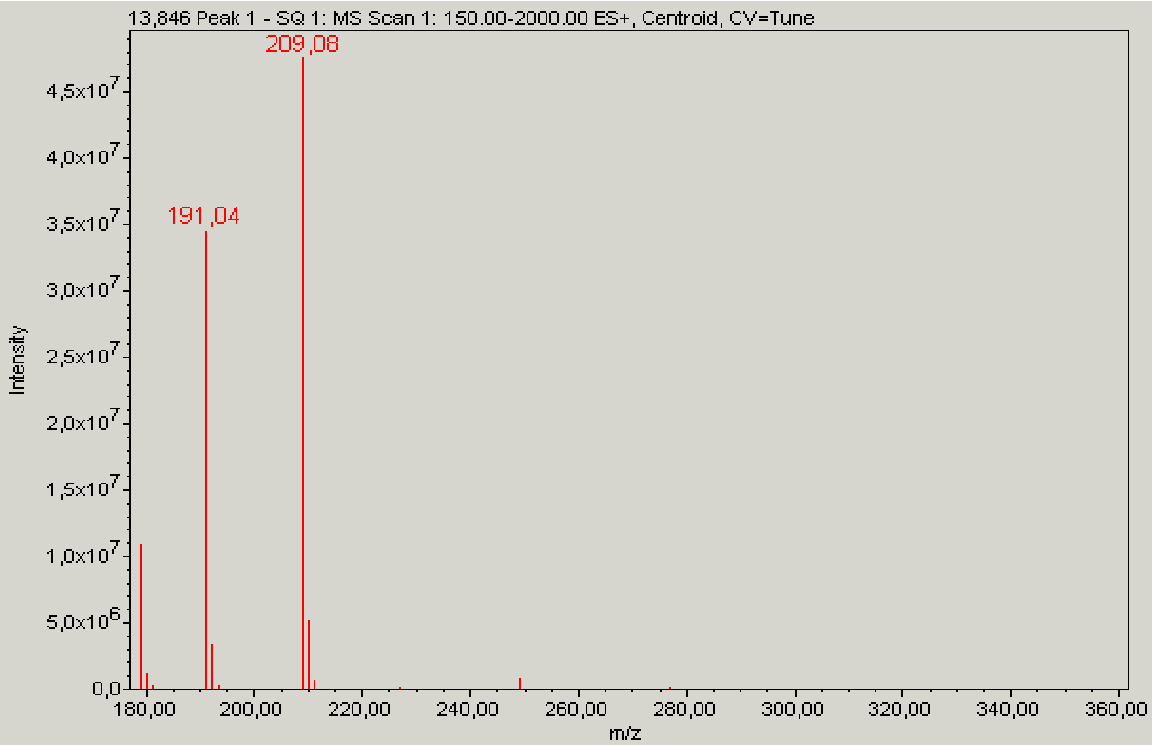

ESI-MS of diol **26a**.

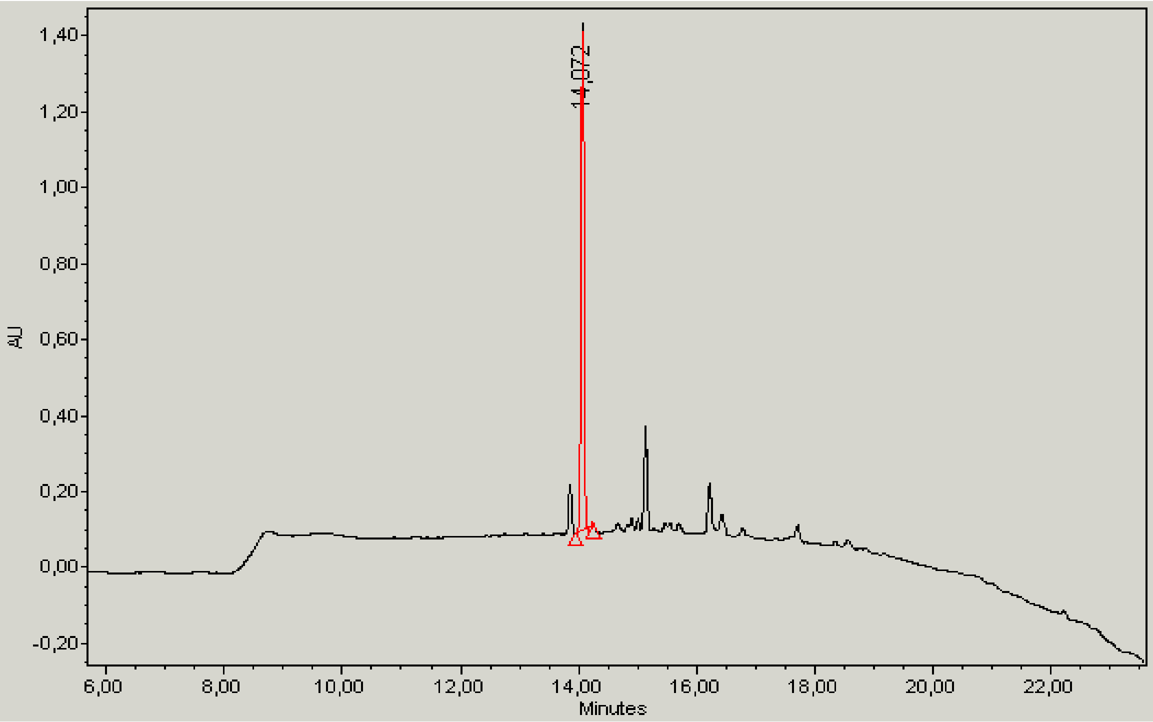

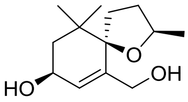

HPLC-chromatogram of diol **26b**, Waters Sunfire 5 µm 250*4.6 mm, flow: 1 mL/min, UV detection 202 nm, gradient 10–90% MeCN in H_2O (0.05% TFA).

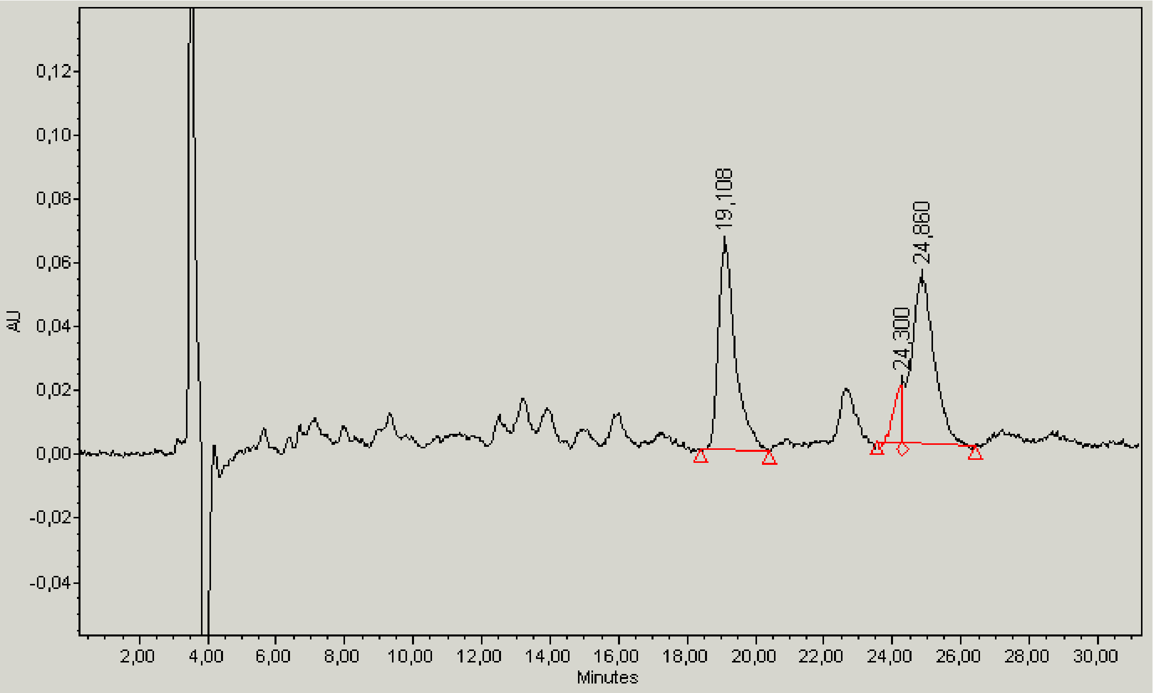

HPLC-chromatogram of diol **26b** on a chiral stationary phase, Chiralpak AS 5 µm 250*4.6 mm, flow: 1 mL/min, UV detection 203 nm, isocratic heptane/EtOH/MeOH 40/1/1 + 0.1% TFA.

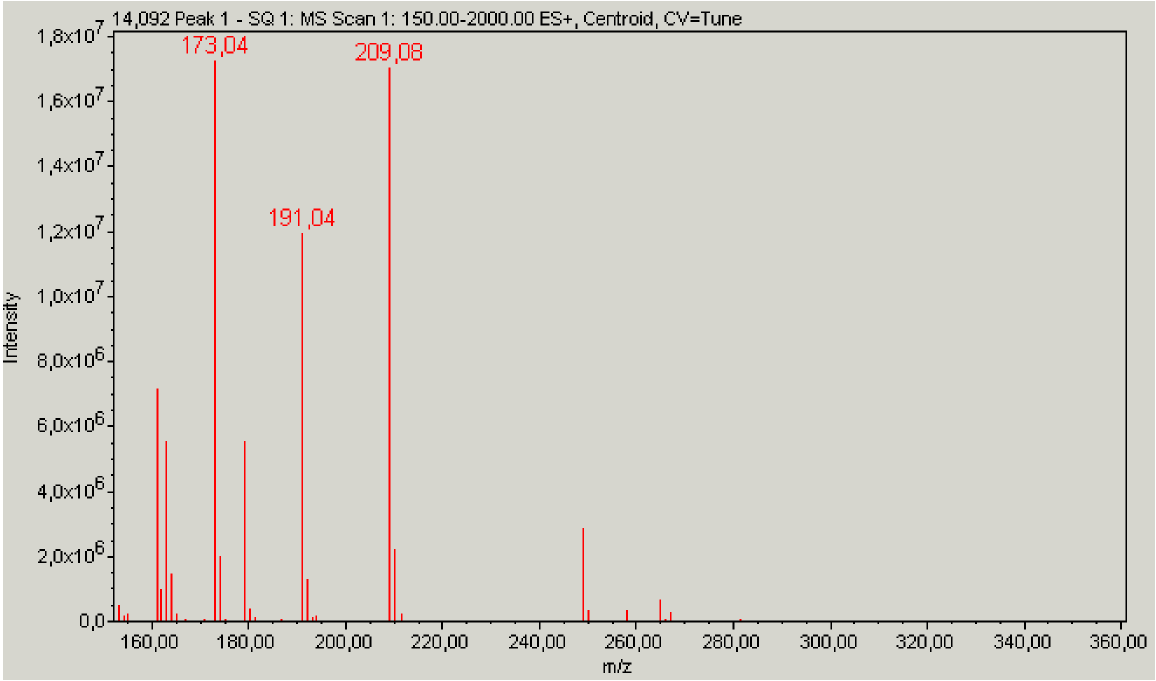

ESI-MS of diol **26b**.

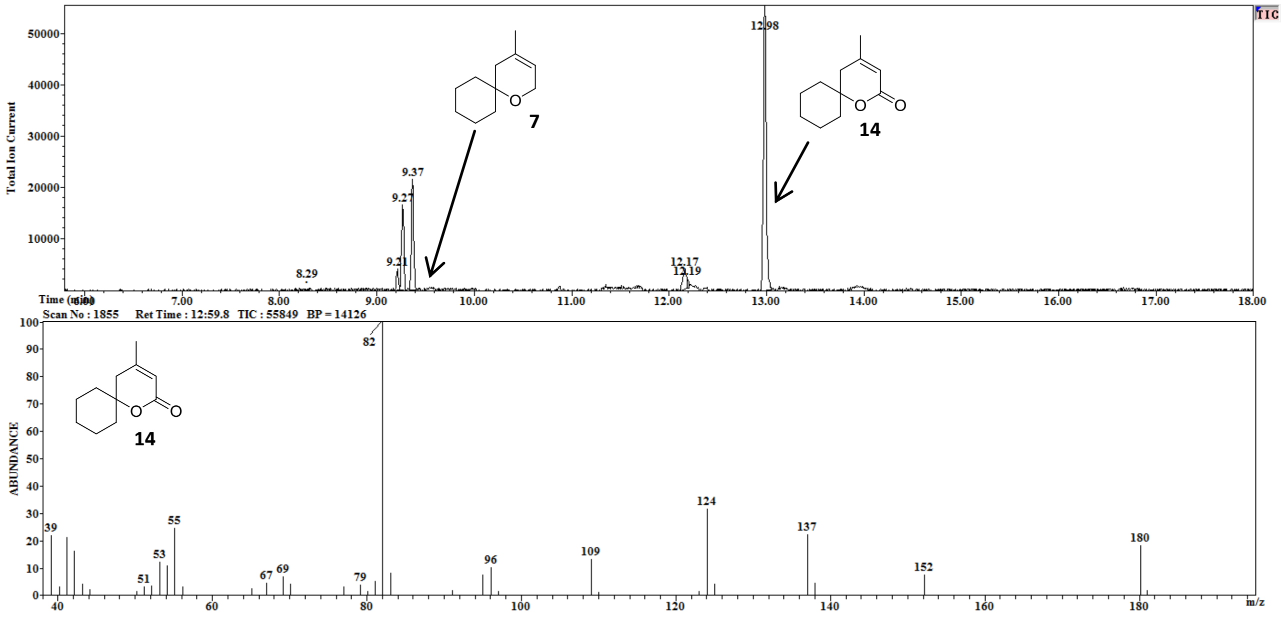

GC–MS of the biocatalytic oxidation of spiroether **7** (crude reaction mixture).

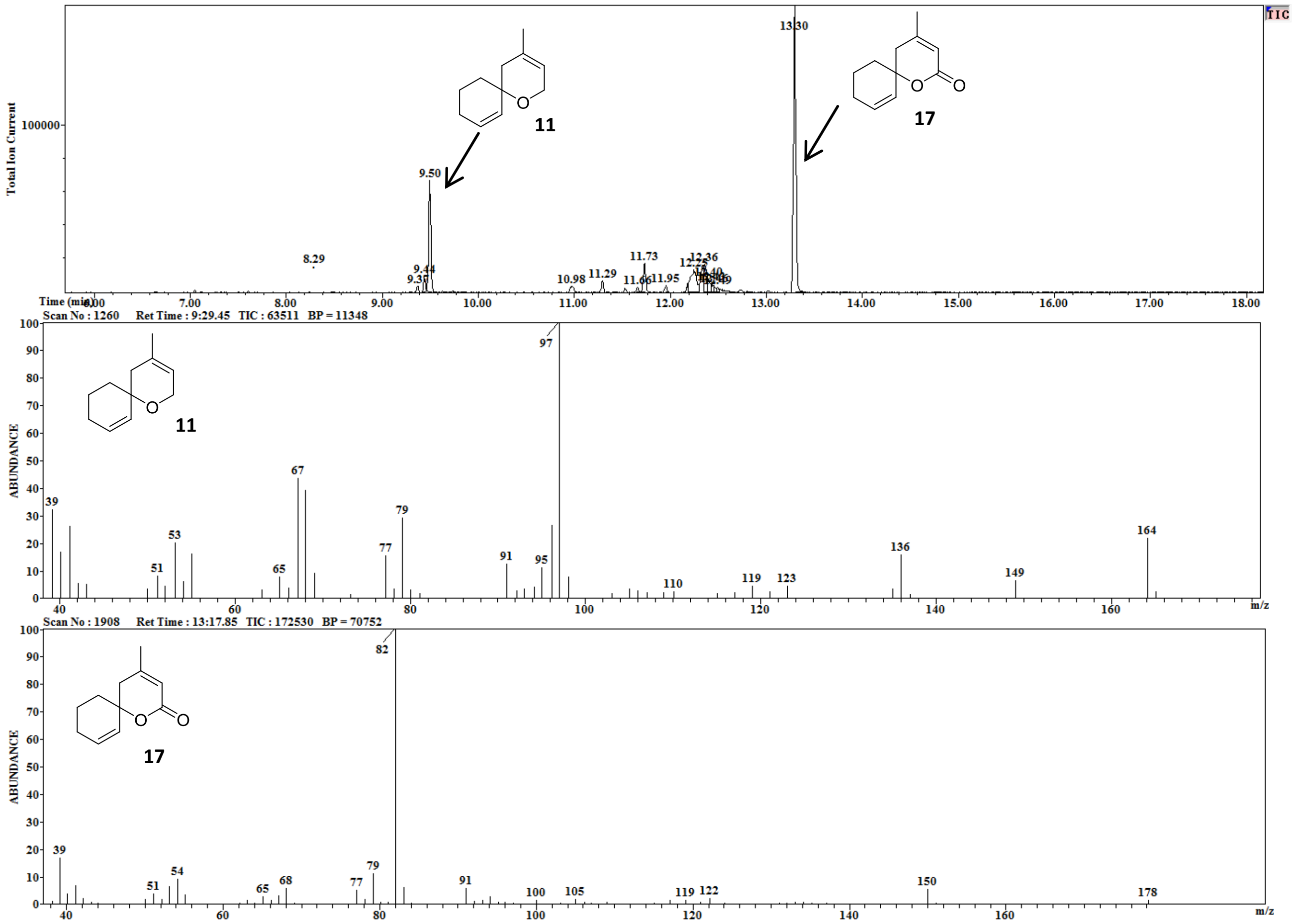

GC–MS of the biocatalytic oxidation of spiroether **11** (crude reaction mixture).

References

1. Frisch, M. J. T., G. W.; Schlegel, H. B.; Scuseria, G. E.; Robb, M. A.; Cheeseman, J. R.; Scalmani, G.; Barone, V.; Mennucci, B.; Petersson, G. A.; Nakatsuji, H.; Caricato, M.; Li, X.; Hratchian, H. P.; Izmaylov, A. F.; Bloino, J.; Zheng, G.; Sonnenberg, J. L.; Hada, M.; Ehara, M.; Toyota, K.; Fukuda, R.; Hasegawa, J.; Ishida, M.; Nakajima, T.; Honda, Y.; Kitao, O.; Nakai, H.; Vreven, T.; Montgomery, Jr., J. A.; Peralta, J. E.; Ogliaro, F.; Bearpark, M.; Heyd, J. J.; Brothers, E.; Kudin, K. N.; Staroverov, V. N.; Kobayashi, R.; Normand, J.; Raghavachari, K.; Rendell, A.; Burant, J. C.; Iyengar, S. S.; Tomasi, J.; Cossi, M.; Rega, N.; Millam, J. M.; Klene, M.; Knox, J. E.; Cross, J. B.; Bakken, V.; Adamo, C.; Jaramillo, J.; Gomperts, R.; Stratmann, R. E.; Yazyev, O.; Austin, A. J.; Cammi, R.; Pomelli, C.; Ochterski, J. W.; Martin, R. L.; Morokuma, K.; Zakrzewski, V. G.; Voth, G. A.; Salvador, P.; Dannenberg, J. J.; Dapprich, S.; Daniels, A. D.; Farkas, Ö.; Foresman, J. B.; Ortiz, J. V.; Cioslowski, J.; Fox, D. J., Gaussian, Inc., Wallingford CT, 2009.
2. Lee, C. T.; Yang, W. T.; Parr, R. G., *Phys. Rev. B* **1988,** *37*, 785-789.
3. Becke, A. D., *J. Chem. Phys.* **1993,** *98*, 5648-5652.
4. Stephens, P. J.; Devlin, F. J.; Chabalowski, C. F.; Frisch, M. J., *J. Phys. Chem.* **1994,** *98*, 11623-11627.
5. Becke, A. D., *Phys. Rev. A* **1988,** *38*, 3098-3100.
6. Hehre, W. J.; Ditchfie.R; Pople, J. A., *J. Chem. Phys.* **1972,** *56*, 2257-2261.
7. Harihara.Pc; Pople, J. A., *Theor. Chim. Acta* **1973,** *28*, 213-222.
8. Nyden, M. R.; Petersson, G. A., *J. Chem. Phys.* **1981,** *75*, 1843-1862.
9. Petersson, G. A.; Bennett, A.; Tensfeldt, T. G.; Allaham, M. A.; Shirley, W. A.; Mantzaris, J., *J. Chem. Phys.* **1988,** *89*, 2193-2218.
10. Petersson, G. A.; Tensfeldt, T. G.; Montgomery, J. A., *J. Chem. Phys.* **1991,** *94*, 6091-6101.
11. Montgomery, J. A.; Ochterski, J. W.; Petersson, G. A., *J. Chem. Phys.* **1994,** *101*, 5900-5909.
12. Montgomery, J. A.; Frisch, M. J.; Ochterski, J. W.; Petersson, G. A., *J. Chem. Phys.* **1999,** *110*, 2822-2827.
13. Montgomery, J. A.; Frisch, M. J.; Ochterski, J. W.; Petersson, G. A., *J. Chem. Phys.* **2000,** *112*, 6532-6542.
14. Cances, E.; Mennucci, B.; Tomasi, J., *J. Chem. Phys.* **1997,** *107*, 3032-3041.
15. Mennucci, B.; Tomasi, J., *J. Chem. Phys.* **1997,** *106*, 5151-5158.
16. Tomasi, J.; Mennucci, B.; Cammi, R., *Chem. Rev.* **2005,** *105*, 2999-3093.
17. McIver, J. W.; Komornic.A, *J. Am. Chem. Soc.* **1972,** *94*, 2625-2633.
18. E. D. Glendening, A. E. R., J. E. Carpenter, and F. Weinhold, *NBO Version 3.1*.
19. Ohta, K., *J. Mol. Struct. (Theochem)* **2002,** *587*, 33-41.
20. Fraatz, M. A.; Riemer, S. J. L.; Stöber, R.; Kaspera, R.; Nimtz, M.; Berger, R. G.; Zorn, H., *J. Mol. Catal. B: Enzym.* **2009,** *61*, 202-207.
21. Schulteelte, K. H.; Gautschi, F.; Renold, W.; Hauser, A.; Frankhauser, P.; Limacher, J.; Ohloff, G. *Helv. Chim. Acta* **1978,** *61,* 1125-1133
22. Marko, I. E.; Mekhalfia, A.; Bayston, D. J.; Adams, H. *J. Org. Chem.* **1992,** *57,* 2211-2213.
